# A Novel Virtual Emergency Medicine Residents-as-Teachers (RAT) Curriculum

**DOI:** 10.21980/J86S71

**Published:** 2021-07-15

**Authors:** Shannon Marie Burke, Thaddeus Schmitt, Corlin Jewell, Benjamin Holden Schnapp

**Affiliations:** * University of Wisconsin BerbeeWalsh, Department of Emergency Medicine, Madison, WI

## Abstract

**Audience:**

The Residents-as-Teachers (RAT) curriculum is designed for emergency medicine (EM) residents of all years (PGY1–4).

**Length of Curriculum:**

The curriculum is divided into three hour-long sessions. The entire curriculum can be run as a single block or can be spread out over multiple days.

**Introduction:**

The Accreditation Council of Graduate Medical Education (ACGME) and the Liaison Committee on Medical Education (LCME) both require residents to receive training in teaching medical students and junior residents. They also require opportunities for residents to participate in teaching and be assessed on their effectiveness in this role.^1^,^2^ However, the ACGME does not provide guidance or require formal curricula on molding residents into effective teachers. Many programs and institutions have incorporated RAT curricula as a solution to provide residents with the skills necessary to create an excellent educational environment for junior learners. These curricula have been embraced by many specialties, including Emergency Medicine (EM).^3^–^6^ The effectiveness of the teaching received during the clinical rotations has important long-term effects on medical students, and may impact their future career choices in medicine.^6^

The COVID-19 pandemic has also required education institutions to vastly alter the delivery of their didactics, including moving to a virtual platform. A completely online format has many benefits that extend beyond the pandemic, such as easier access to participants (including those off-service or at remote sites), no requirement for a physical space, and easier recording of sessions.

**Educational Goals:**

To provide residents with an introduction to teaching techniques that can be utilized on-shift to facilitate an excellent educational experience for junior learners while balancing the resident’s patient care responsibilities.

**Educational Methods:**

The educational strategies used in this curriculum include PowerPoint (Redmond, WA) slideshows given by a live presenter via the telecommunications platform Zoom (San Jose, CA), viewing of videos demonstrating curriculum topics, simulation-based learning through role-play, and small-group discussions including simulation debriefing.

**Research Methods:**

A survey was distributed to residents before and following the completion of the three training sessions to assess resident satisfaction with the delivery of the content and comfort with the teaching tools discussed. Suggestions on potential improvements were also assessed to inform changes to future iterations of the curriculum. Comfort regarding the included teaching tools was assessed using a five-point Likert scale. After completion of the curriculum, rotating medical students were provided with an evaluation form to assess if residents were teaching using the techniques from the course.

**Results:**

Both the pre-curriculum and post-curriculum surveys had a response rate of 61.1%. Student’s t-test showed a statistically significant increase in mean resident comfort level with the teaching strategies post-curriculum (3.05 to 3.83, p < 0.01). Medical student evaluations have shown, overall, that the majority of residents are utilizing the education techniques on-shift. There were no significant differences found in medical student perception of resident use of taught skills between those who had and had not attended the sessions. However, all but one assessed skill showed higher utilization in those who had attended the correlating session.

**Discussion:**

The educational content was effective in improving the residents’ comfort with the teaching strategies presented, and residents are utilizing these techniques on-shift. Through implementation, we discovered that presenting a curriculum over video conferencing required additional administrative support to help ensure efficacy of break-out groups. Based on resident feedback after the first session, multiple changes were made, including providing residents with hand-out references for use during the role-playing sessions. The success of this curriculum demonstrated the feasibility and utility of running a RAT curriculum entirely in a virtual format.

**Topics:**

Residents-as-teachers, distance learning, role-playing, virtual curriculum, video conferencing, One Minute Preceptor, feedback, “What if?” game, Aunt Minnie, SPIT, activated demonstration, self-directed teaching tools, teaching scripts, Post-It Pearls.

## USER GUIDE

[Table t2-jetem-6-3-c8]

**Table t2-jetem-6-3-c8:** 

List of Resources:	
Abstract	9
User Guide	11
Didactics and Hands-On RAT Curriculum Chart	16
Appendix 1a: Pre-Curriculum Survey	17
Appendix 1b: Post-Curriculum Survey	18
Appendix 1c: Medical Student Evaluation of Residents	19
Appendix 2a: OMP Facilitator Guide	21
Appendix 2b: OMP Handout	22
Appendix 2c: RAT Introduction	27
Appendix 2d: OMP Lecture	28
Appendix 2e: OMP Scenario 1: Medical Student Script	29
Appendix 2f: OMP Scenario 1: Resident Script	30
Appendix 2g: OMP Scenario 2: Medical Student Script	31
Appendix 2h: OMP Scenario 2: Resident Script	32
Appendix 2i: OMP Scenario 3: Medical Student Script	33
Appendix 2j: OMP Scenario 3: Resident Script	34
Appendix 3a: Feedback Facilitator Guide	35
Appendix 3b: Feedback Handout	39
Appendix 3c: OMP Recap Slides	41
Appendix 3d: Feedback Lecture	42
Appendix 3e: Feedback Example Scenario	43
Appendix 3f: Feedback Scenario 1: Medical Student Script	45
Appendix 3g: Feedback Scenario 1: Resident Script	46
Appendix 3h: Feedback Scenario 2: Medical Student Script	47
Appendix 3i: Feedback Scenario 2: Resident Script	48
Appendix 3j: Feedback Scenario 3: Medical Student Script	49
Appendix 3k: Feedback Scenario 3: Resident Script	50
Appendix 4a: T&T Facilitator Guide	51
Appendix 4b: T&T Handout	54
Appendix 4c: Feedback Recap Slides	56
Appendix 4d: T&T Lecture	57
Appendix 4e: T&T Scenario 1: Medical Student Script	58
Appendix 4f: T&T Scenario 1: Resident Script	59
Appendix 4g: T&T Scenario 2: Medical Student Script	60
Appendix 4h: T&T Scenario 2: Resident Script	61
Appendix 4i: T&T Scenario 3: Medical Student Script	62
Appendix 4j: T&T Scenario 3: Resident Script	62

**Learner Audience:**

Interns, Junior Residents, Senior Residents

**Length of Curriculum:**

This curriculum consists of three one-hour sessions. These sessions were spaced out over three months, according to availability in the pre-scheduled conference time for residents.

**Topics:**

Residents-as-teachers, distance learning, role-playing, virtual curriculum, video conferencing, One Minute Preceptor, feedback, “What if?” game, Aunt Minnie, SPIT, activated demonstration, self-directed teaching tools, teaching scripts, Post-It Pearls.

**Objectives:**

By the end of this curriculum, learners will:

1. Review the One Minute Preceptor methods of teaching and be able to implement this technique on-shift.
2. Demonstrate the ability to provide effective, actionable end-of-shift feedback.
3. Understand and use a variety of on-shift teaching methods.

### Brief introduction

The Accreditation Council for Graduate Medical Education (ACGME) requires that residents acquire the skills necessary to teach and assess learners.^1^,^2^ Teaching courses for residents are shown to improve their confidence as teachers as well as improve student evaluations of residents on-shift.^7^,^8^ In a previous study, medical students perceived that the majority of the clerkship’s on-shift educational objectives teaching was from the residents as opposed to the attendings.^9^ Even though not all residents will ultimately pursue a career in academics, effective teaching skills are still important in the community because these physicians are often responsible for instructing advanced practice providers, local prehospital agencies, and nursing staff at their institutions. Therefore, it is critical to provide residents with formal instruction on how to effectively teach while on-shift.

The Emergency Department (ED) is a rich source of educational opportunities, but also presents many obstacles to educators, including time limitations, frequent interruptions, and lack of physical space for teaching.^10^ Therefore, it is important to prepare emergency medicine (EM) residents to teach effectively while still directing efficient patient care. We present a virtual curriculum that utilizes lecture, role-playing, and debriefing to educate residents on the One Minute Preceptor, providing effective feedback and other on-shift teaching tools (eg, Aunt Minnie, Post-it Pearls, SPIT) for teaching effectively in the ED.

COVID-19 has led to many innovations in medical education due to the need for social-distancing, such as the implementation of remote curricula via online video-conferencing.^11^ Sessions in which procedures and other skills are provided over a video-conferencing platform have been successful previously.^12^ Therefore, due to the inability of residents to gather in person, our curriculum was designed so that it could be presented entirely over video conferencing software, such as Zoom (San Jose, CA). In light of the current global pandemic, and the increasingly important role of online educational delivery, this curriculum represents a novel way to ensure residents are receiving instruction on a critical topic in a safe and effective manner.

We believe that even after pandemic-related restrictions are lessened, the online format of this curriculum will continue to be beneficial. First, it decreases the need for a large physical space to conduct the sessions (which is increased by the need to conduct the breakout role-playing sessions). Second, it can allow individuals who are unable to attend in person, such as those on off-service or remote rotations, to participate. Finally, the online format facilitates easy recording of the didactic portions of the sessions so that they can be reviewed by those who could not attend.

### Problem identification, general and targeted needs assessment

The impetus behind our decision to devise and implement our virtual RAT curriculum was based on a restructuring of our institution’s medical student curriculum to emphasize earlier exposure to clinical environments, including the ED, during the M2 year. This resulted in the expansion of teaching interactions between residents and junior medical students on-shift. There has also been a departmental focus on improving the quality of the educational experience to medical students during their EM clerkship. This prompted discussions between program leadership and residents which revealed resident concerns about their ability to provide effective and efficient teaching on-shift while continuing to manage patient care.

RAT curricula have been created across different specialties and use a variety of techniques such as lecture, role-playing, simulation, and objective structured teaching exercises (OSTE).^7^ Efficacy of published curricula have also been evaluated according to different levels of the Kirkpatrick’s training evaluation model.^13^ In a 2017 survey, 80% of programs had a RAT curriculum; 90% of these curricula included lectures and approximately half included role-playing.^14^ While RAT curricula have been studied extensively, the educational clearinghouses of MedEdPORTAL and the Journal of Education and Teaching in Emergency Medicine (JETem) have very few published RAT curricula that can be rapidly adopted by other programs.^14^–^21^ Only one of these curricula is aimed specifically at EM residents.^14^ The Emergency Department is a unique teaching environment, with variable pace, acuity, and patient volume, and therefore it is important to have curricula available that address the needs of residents specifically teaching in the ED.

Unlike existing curricula, this RAT curriculum was designed to be implemented in a completely virtual format, made necessary due to medical students returning to the clinical environment while educational conferences were still required to be virtual. We felt that it was essential to maintain an interactive component, and therefore role-playing was utilized to allow residents to practice skills acquired in the lectures.

To create the ideal content for our curriculum, we reviewed the literature for high yield topics for a resident-focused intervention. Following the literature search, potential topics most important to EM residents were decided upon by the author group, which consisted of senior EM residents and residency program leadership at a single academic institution. Based on the results of this discussion, the residents were then surveyed to select the three topics that they felt would be most valuable. The chosen sessions were the One Minute Preceptor, Providing Effective Feedback, and Tips and Tricks for Teaching on Shift. The Tips and Tricks sessions included a variety of on-shift teaching tools (eg, Aunt Minnie, Post-it Pearls, SPIT).

This curriculum was intended to enhance the medical student experience by changing resident behavior while teaching on-shift. It was also intended to improve resident confidence in on shift teaching and their ability to balance their time between clinical duties and teaching responsibilities.

### Goals of the curriculum

The Residents as Teachers curriculum aimed to provide residents with tools needed to facilitate effective on-shift medical student education via an entirely virtual format. Individual sessions focused on the One Minute Preceptor teaching method, provision of high-quality feedback, and effective integration of teaching into busy clinical shifts.

### Objectives of the curriculum

By the end of this curriculum, learners will:

1. Review the One Minute Preceptor methods of teaching and be able to implement this technique on-shift.
2. Demonstrate the ability to provide effective, actionable end-of-shift feedback.
3. Understand and use a variety of on-shift teaching methods.

### Educational Strategies

See Curriculum Chart

### Results and tips for successful implementation

The curriculum was implemented through a series of three virtual, video-based sessions held through October–December of 2020, with approximately 8–12 resident learners available per session. 22 out of 36 residents were able to attend at least one session, with 5 residents being able to attend two sessions. Only two residents were able to attend all three sessions. The relatively low number of residents that were able to participate completely was primarily due to residents’ clinical duties interfering with their ability to attend weekly didactic conference. Programs desiring to expose a greater portion of their residents to the curriculum could seek to create a small amount of protected time for their residents and/or condense the sessions into a single day. Each session consisted of an introductory lecture followed by several small group break-out sessions for role-playing scenarios with facilitated debriefings. These role-playing scenarios and debriefing sessions allowed residents to practice the concepts introduced in the lecture. The first session included a brief introduction to the overall curriculum. Subsequent sessions began with a brief overview of the preceding session to facilitate retention of knowledge through spaced repetition. The sessions were presented in the following order: One-Minute Preceptor, Feedback, and Tips and Tricks.

**Figure 1:**
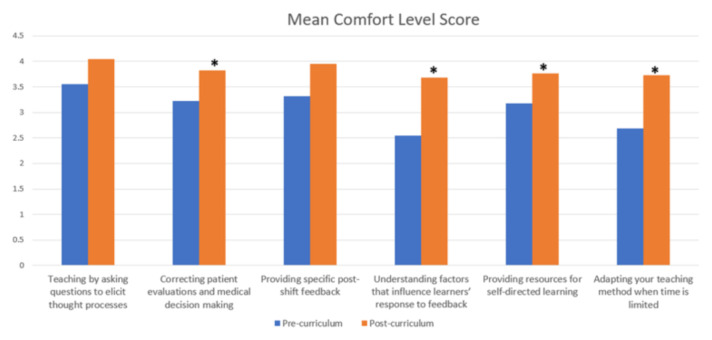
Resident mean comfort levels with queried topics pre- and post-curriculum (*p <0.05).

Evaluation of the sessions was completed using both pre- and post-curriculum surveys, which included a series of 6 questions targeting the teaching techniques emphasized by the curriculum (Figure 1). In total, residents reported a statistically significant increase in comfort with the teaching strategies when considered as a whole (3.05 to 3.83, p < 0.01). Mean comfort was increased in every question assessed, with residents reporting the most significant increase in comfort in understanding learners’ response to feedback (2.55 to 3.68, p < 0.001).

Resident behavior following the session was assessed through post-shift surveys distributed to second year medical students during their 2-week required EM rotations. Students were asked to respond to five questions regarding whether residents used skills taught in each of the individual sessions (discussing expectations and goals, teaching principles, providing post-shift feedback, eliciting student input on feedback, and utilizing a variety of teaching techniques) after every shift. Resident attendance of individual sessions was determined using an attendance log, and answers were compared between residents who did and did not attend the sessions using a Chi-square test for all questions except the question regarding teaching principles, which was analyzed using Fisher’s Exact Test (Table 1).

No significant differences were seen in medical student perception of resident use of taught skills between those who had and had not attended the sessions, although all but one assessed skill (utilizing a variety of teaching techniques) showed higher skill utilization in those who had attended the correlating session. These results were limited by a small *n* of individual residents who were able to attend each individual teaching session, and the high amount of “yes” responses by students to each question.

### Evaluation and Feedback

After completion of the first session, both instructors and learners reported issues with the break-out groups, with instructors noting difficulties in managing splitting groups through the software while trying to lead discussions and guide the breakout groups. To address these difficulties, we decided to add an outside facilitator (in this case, our administrative assistant who was experienced with managing Zoom breakout rooms) to separate residents into groups and keep track of timing. Ideally, this individual should not be one of the session facilitators to allow them to travel between small groups unhindered to observe the role-playing activities and to stimulate discussion.

Learners further reported uncertainty about expectations and best practices during the role-playing sessions following the first session. Future sessions were adapted with the addition of a feedback video and acted scenario by facilitators for the feedback session. Hand-out materials were also provided during sessions to supplement slide-based presentations so that residents could reference the high-yield information from the lecture while completing the role-playing scenarios. The handouts were created on Google Drive (Mountain View, CA) and included links to each of the role-playing scenario scripts at the end. We initially encountered permissions issues with some of these files when residents tried to access them unsuccessfully; appropriately broad sharing settings for all files should be confirmed prior to the start of the exercise. Once this was resolved, we found Google Drive to be a platform where the residents were easily able to access all the tools they needed for the role-playing scenarios.

### Associated Content

Curriculum Chart

Appendix 1: Research Tools

Appendix 1a: Pre-curriculum survey

Appendix 1b: Post-curriculum survey

Appendix 1c: Medical student evaluation of residents

Appendix 2: One Minute Preceptor (OMP) Session

Appendix 2a : OMP Facilitator Guide

Appendix 2b : OMP Hand-out

Appendix 2c : RAT introduction

Appendix 2d : OMP Lecture

Appendix 2e: OMP, Scenario 1, Medical Student Script

Appendix 2f: OMP, Scenario 1, Resident Script

Appendix 2g: OMP, Scenario 2, Medical Student Script

Appendix 2h: OMP, Scenario 2, Resident Script

Appendix 2i: OMP, Scenario 2, Medical Student Script

Appendix 2j: OMP, Scenario 3, Resident Script

Appendix 3: Feedback Session

Appendix 3a: Feedback Facilitator Guide

Appendix 3b: Feedback Hand-out

Appendix 3c: OMP Recap Slides

Appendix 3d: Feedback Lecture

Appendix 3e: Feedback Example Scenario

Appendix 3f: Feedback, Scenario 1, Medical Student Script

Appendix 3g: Feedback, Scenario 1, Resident Script

Appendix 3h: Feedback, Scenario 2, Medical Student Script

Appendix 3i: Feedback, Scenario 2, Resident Script

Appendix 3j: Feedback, Scenario 2, Medical Student Script

Appendix 3k: Feedback, Scenario 3, Resident Script

Appendix 4: Tips and Tricks (T&T) Session

Appendix 4a: T&T Facilitator Guide

Appendix 4b: T&T Hand-out

Appendix 4c: Feedback Recap Slides

Appendix 4d: T&T Lecture

Appendix 4e: T&T, Scenario 1, Medical Student Script

Appendix 4f: T&T, Scenario 1, Resident Script

Appendix 4g: T&T, Scenario 2, Medical Student Script

Appendix 4h: T&T, Scenario 2, Resident Script

Appendix 4i: T&T, Scenario 2, Medical Student Script

Appendix 4j: T&T, Scenario 3, Resident Script

## Figures and Tables

**Table t3-jetem-6-3-c8:** 

Topic	Recommended Educational Strategy	Educational Content	Objectives	Learners	Timing, Resources Needed (Space, Instructors, Equipment, Citations of JETem pubs or other literature)	Recommended Assessment, Milestones Addressed
One-Minute-Preceptor	Introduction to overall RAT curriculum. Lecture on microskills. Role-playing sessions in virtual small groups with debriefs after each session.	-Overall review of One-Minute-Preceptor teaching technique. - Setting learner-specific goals and expectations. - Assessing medical students’ thought processes and how to probe them for additional information. - Guidance for providing mid-shift feedback that can guide further learning.	The resident will be able to develop expectation s with students at the start of every shift based on student’s level. The resident will be able to define the key elements of the One-Minute Preceptor model and implement these on-shift.	PGY1-PGY3 EM Residents	25 minutes (lecture) Instructors: 1 Equipment: Computer, projector/screen. 35 minutes (role playing sessions) Instructors: 1 per 2–3 resident learners. Equipment: individual laptops with web-cam and microphone capabilities.	Assessment: demonstration of proficiency in virtual role-playing scenarios. Milestones: Team Management (ICS2) Leads patient-centered care teams, ensuring effective communication and mutual respect among members.
Feedback	Review of prior session. Lecture on providing feedback. Virtual roleplaying scenarios and debriefs.	-Defining elements of effective feedback. - Determining factors that influence medical student response to feedback. - Identifying techniques for delivering effective feedback. - Deconstructing examples of common ineffective feedback.	The resident will be able to recognize and define aspects of effective feedback. The resident will be able to apply aspects of effective feedback in role-playing scenarios and on-shift. The resident will be able to identify and define common pitfalls of feedback.	PGY1-PGY3 EM Residents	25 minutes (lecture). Instructors: 1 Equipment: Computer, projector/screen. 35 minutes (role playing sessions, debriefs). Instructors: 1 per 2–3 resident learners. Equipment: individual laptops with web-cam and microphone capabilities.	Assessment: demonstration of proficiency in virtual roleplaying scenarios. Milestones: Team Management (ICS2) Leads patient-centered care teams, ensuring effective communication and mutual respect among members.
Tips and Tricks	Review of prior session. Lecture on different on-shift teaching techniques. Virtual roleplaying scenarios with debriefs.	-Illustrating examples of various teaching techniques (“What If?” game, Aunt Minnie, SPIT, mini-lectures, activated demonstrations). - Comparing and contrasting a variety of tools based on available on-shift time. - Identifying resources to guide further student learning.	Residents will be able to describe and implement multiple on-shift learning tools based on available time. Residents will be able to design and provide short, topic-focused lectures for on-shift teaching.	PGY1-PGY3 EM Residents	25 minutes (lecture). Instructors: 1 Equipment: Computer, projector/screen. 35 minutes (role playing sessions, debriefs). Instructors: 1 per 2–3 resident learners. Equipment: individual laptops with web-cam and microphone capabilities.	Assessment: demonstration of proficiency in virtual roleplaying scenarios. Milestones: Team Management (ICS2) Leads patient-centered care teams, ensuring effective communication and mutual respect among members.

**Table 1:** Medical student “Yes” responses when asked if a resident used specific skills on-shift.

	Attended Session	Did Not Attend Session	p-value
Discussed Expectations and Goals (One-Minute Preceptor)	95.2% (n = 21)	88.2% (n = 34)	0.38
Taught General Principles (One-Minute Preceptor)	100% (n = 21)	94.1% (n = 34)	0.52
Provided Post-Shift Feedback (Feedback)	90.0% (n = 20)	82.9% (n = 35)	0.47
Asked for Student Input on Feedback (Feedback)	90.0% (n = 20)	85.7% (n = 35)	0.64
Utilized Variety of Teaching Techniques (Tips and Tricks)	92.3% (n = 13)	97.6% (n = 42)	0.37

## DIDACTICS AND HANDS-ON CURRICULUM

[Table t3-jetem-6-3-c8]

## Appendix 1a Pre-Curriculum Survey

Anonymous identifier questions:

1. What are the first two letters of your mother’s name? _____________
2. What city were you born in? _____________

Please rate your level of comfort with:[Table t4-jetem-6-3-c8]

**Table t4-jetem-6-3-c8:**

	Very uncomfortable	Somewhat uncomfortable	Neither comfortable nor uncomfortable	Somewhat comfortable	Very comfortable
Providing specific feedback regarding behaviors or actions of a learner on shift.					
Leading a medical student through a patient encounter by asking questions to understand his or her thought processes.					
Adapting your teaching method when time is limited.					
Correcting a student’s patient evaluation and medical decision making.					
Providing medical students with resources for self-directed on-shift learning.					
Understanding factors that influence learners’ response to feedback.					

## Appendix 1b Post-Curriculum Survey

Anonymous identifier questions:

1. What are the first two letters of your mother’s name? _____________
2. What city were you born in? _____________

Please rate your level of comfort with:[Table t5-jetem-6-3-c8]

**Table t5-jetem-6-3-c8:**

	Very uncomfortable	Somewhat uncomfortable	Neither comfortable nor uncomfortable	Somewhat comfortable	Very comfortable
Providing specific feedback regarding behaviors or actions of a learner on shift.					
Leading a medical student through a patient encounter by asking questions to understand his or her thought processes.					
Adapting your teaching method when time is limited.					
Correcting a student’s patient evaluation and medical decision making.					
Providing medical students with resources for self-directed on-shift learning.					
Understanding factors that influence learners’ response to feedback.					

Additional questions:

1. Which Residents-as-Teachers sessions did you attend? (select all that apply)
  - One Minute Preceptor
  - Feedback
  - Tips and Tricks for Teaching On-Shift
2. What aspect(s) did you feel were effective for learning the Residents-as-Teachers concepts? (select all that apply)
  - Lectures
  - Role-playing scenarios
  - 5-minute recaps
  - None
3. What improvements would you make to the Residents-as-Teachers sessions
4. Do you plan to implement what you learned during the Residents-as-Teachers sessions to your clinical shift experience when working with learners? Why or why not?
5. Please provide any additional comments you have regarding the One Minute Preceptor session.
6. Please provide any additional comments you have regarding the Feedback session.
7. Please provide any additional comments you have regarding the Tips and Tricks for Teaching On-shift session.

## Appendix 1c Medical Student Evaluation of Residents

[Table t6-jetem-6-3-c8]

**Table t6-jetem-6-3-c8:**

Resident as Teacher Questionnaire								
Please complete this questionnaire following each shift and place it in the post-shift evaluation lock box. Ask the attending where the box is located if you do not know. Please do not review this questionnaire with your resident.								
1.	Which resident were you paired with today? ________________________________________							
2.	This is the _______________ shift I have worked with this resident (eg, 1^st^, 2^nd^, 3^rd^….).							
3.	Did the resident discuss expectations or goals at the beginning of your shift?						Yes	No

4.	Did the resident provide you with specific post-shift feedback?						Yes	No

5.	Did the resident ask for your input on what you wanted feedback on at the end of shift?						Yes	No

6	Did the resident utilize a variety of techniques to teach throughout the shift? (eg, provided mini-lectures on specific topics, asked how changing facts of the case might change your differential and management, discussed a topic they asked you to research, discussed differential diagnoses in different ways)						Yes	No

7.	Did resident teach principles (eg, approach to abdominal pain in cirrhosis, follow up instructions for simple lacerations) that could be helpful for future patients with similar complaints?						Yes	No
8.	Please rate the overall quality of your learning experience with the resident today when compared to resident teaching experiences on other clinical rotations.							
		a.	Poor	Fair	Good	Excellent		
9.	Please provide any further comments you may have regarding your educational experience with the resident on shift today (continue on back of card if needed):							

## Appendix 2a One Minute Preceptor Session Facilitator Guide

### Objectives

Following completion of this session, residents will be adept at:

- Developing expectations with learner at the start of each of their shifts based on the level of the learner
- Identifying and defining the different aspects of the One-Minute Preceptor model.
- Implementing the One-Minute Preceptor model during each patient encounter with medical students on shift.

### Pre-Session Preparation

- It is strongly recommended that the session handouts and the scenario scripts are loaded into a GoogleDrive folder. These can then be linked to the scenario scripts into the bottom of the handouts for ease of access.
- Ensure that sharing permissions are correct (eg, in Google Drive, the option “everyone with the link can view” is selected under the “Share” options).

### Roles

- *Lecturer*: Will provide the main lecture for the session and can also provide the recap on the previous session. Alternatively, you can choose a second lecturer to provide the recap from the previous session.
- *Video Conferencing Facilitator:* Recommend that these persons be separate from the facilitators for the small groups. They will assign participants to the break out rooms, move Small Group Facilitators between the breakout rooms, and keep time for the breakout sessions. Also recommend that these persons have access to the handout and scenario script links to send out in the video conferencing chat.
- *Small Group Facilitators*: Will also likely include the lecturer. Depending on the number of small groups, these facilitators can stay with one group or be moved through different groups by the Video Conferencing Facilitator. These facilitators should be experienced educators who are prepared to answer questions regarding the topics presented in the lecture so that they can efficiently guide the scenarios and participate in the debriefs.

### Timing Outline

0000–0005: Curriculum introduction PowerPoint

0005–0025: Lecture on Feedback

0025: Separate into small groups

0025–0030: 1st role-playing scenario

0030: Return to large group

0030–0035: Debrief scenario 1

0035: Separate into small groups

0035–0040: 2nd role-playing scenario

0040: Return to large group

0040–0045: Debrief role-playing 2

0045: Separate into small groups

0045–0050: Role-playing scenario 3

0050: Return to large group

0050–0055: Debrief role-playing scenario 3

0055: Final comments and end session

The timing once the role-playing scenarios start is more fluid and is also dependent on how the learners work through the scenarios. Recommend communicating back to the Video Conferencing Facilitator to end the role-playing scenarios as groups finish to start the debriefing discussions.

### Content Outline

#### Lecture

- Discuss importance of setting expectations with junior learners (medical students, off-service residents, interns) and orienting them to the ED (and how it is different from other specialties’ environments)
- How to identify the level of the learner (briefly so you know what expectations to set for them)
- Define the different steps of the one-minute preceptor
- Note that the content provided here adds the additional two steps proposed by Kansas University Medical Center for a total of seven
- Provide examples of the different steps of the One-Minute-Preceptor model

#### Role-playing scenarios

- Groups can be 2 or 3 residents, with one playing the student, one playing the resident, and an optional observer (in the case of groups of 3)
- The third case can be considered optional if the discussion of the first two scenarios runs long
- Ideally, there should be enough scenarios to allow each participant to play each role at least once
- Ensure that all participants have access to the handout to reference prior to the start of the role-playing scenarios
- Provide scripts for the student and resident portions of each scenario via links
  - Recommend including these in the handout reference
- If desired, can direct the observer (if present) to look at specific aspects of the interaction (eg, one thing that was done really well and one thing that could be improved).

#### Debrief

- Questions/plan based on the following stages of debriefing:
  - Emotional response: elicit resident’s emotions about the case
    - Example: Did the scenario remind the resident of prior interactions with medical students on-shift?
- Solicit examples from residents about what they thought went well in their interaction that others could emulate
- Determine what steps of the One Minute Preceptor model were most difficult to implement and discuss ways to overcome barriers to using the model on shift
- Re-emphasize the importance of discussing expectations and goals with the student early so that the clinical experience is learner-focused
- Address any knowledge gaps and correct assumptions
- Summarize 1–2 important take-aways from the case and discussion

### Resources

- One Minute Preceptor. Kansas University Medical Center. Sept 28, 2018. http://www.kumc.edu/school-of-medicine/som-faculty-affairs-and-development-(fad)/preceptor-resources/one-minute-preceptor.html
- Furney SL, Orsini AN, Orsetti KE, Stern DT, Gruppen LD, Irby DM. Teaching the One-Minute Preceptor: A Randomized Control Trial. *J Gen Intern Med*. 2001; 16(9): 620–624. doi:10.1046/j.1525-1497.2001.016009620.x
- The One Minute Preceptor [Video]. Youtube. Published December 17, 2012. Accessed August 21, 2020. At: https://www.youtube.com/watch?v=P0XgABFzcgE

## Appendix 2b One Minute Preceptor Handout

### What is it?

- Originally a five-step teaching model developed by Neher et al. in 1992
  - Two steps, steps 1 and 7, later added by Kansas University
- Not necessarily meant to be used in a strict order, though the first and last steps are designed to be used at the beginning and end, respectively, of the teaching encounter

### Step 1: Set Goals and Expectations

- Determine the level of the learners you are working with and goals they may have
- Set clear expectations for the learners that are appropriate for their level and goals
- Provide a clear timeline for activities with the learners
  - “Take 15 minutes to gather the history from this patient and then meet me here.”

### Step 2: Get a Commitment

- Ensure the learners are providing their own thoughts and ideas
  - Avoid feeding them answers to questions too early
- Ask “What” Questions
  - “What do you think is going on?”
  - “What antibiotics would you use to treat this infection?”
- If present, try to determine the reason for reluctance displayed by the learner
  - Reluctance may be multifactorial (feel intimidated, passive learning style, etc.)

### Step 3: Probe for Supporting Evidence

- Ask “Why” Questions
  - “Why would you order a complete metabolic panel on this patient?”
  - Ensures understanding of the medical topics being discussed

### Step 4: Reinforce what was Done Well

- Provide SPECIFIC positive feedback
  - “You did a great job talking with that patient’s family!”

### Step 5: Correct Mistakes

- Don’t be afraid to point out mistakes so that a learner may correct them in the future
- Provide SPECIFIC and ACTIONABLE feedback to the learner
  - “Next time, I would try rehearsing the consult call before paging surgery.”
- In many circumstances, it is most appropriate to correct mistakes away from public view

### Step 6: Teach General Rules

- Provide details on how learned medical pearls can be used in future patient encounters
- Learners like generalizability
- Point them towards appropriate literature (including online blogs) to build upon what was learned

### Step 7: Encourage Reflection and Integration

- Allow a set time for reflection to facilitate integration into prior knowledge
- Debriefing should occur immediately after teaching encounter (or after important patient care tasks) to provide greatest impact
- Should elicit topics of discussion from the learner
- Guides their studies after shift

### Role-Playing Scenarios

- Scenario 1:
  - Resident Script: *insert link here*
  - Medical Student Script: *insert link here*
- Scenario 2:
  - Resident Script: *insert link here*
  - Medical Student Script: *insert link here*
- Scenario 3:
  - Resident Script: *insert link here*
  - Medical Student Script: *insert link here*

## Appendix 2c Residents as Teachers Introduction Lecture

Please see associated PowerPoint file

## Appendix 2d One Minute Preceptor Lecture

Please see associated PowerPoint file

## Appendix 2e One Minute Preceptor Scenario 1: Medical Student Script

Setting: You are a nervous third-year medical student in the ED on one of your first rotations. Please begin with presenting the assessment and plan.

Patient: 57-year-old Male with Chest Pain

HPI: Pain began while cleaning out his garage 2 hours ago. 7/10 in severity. No similar episodes in the past. He thinks it is his GERD.

Past medical history: hypertension (HTN), diabetes mellitus (DM)

Medications: Lisinopril, Metformin

Family history: coronary artery disease (CAD)

Social history: Daily Smoker

Physical exam: Unremarkable

**Your Assessment & Plan:** This is a 57-year-old male with chest pain starting 2 hours ago. Could be his GERD so he should take a proton-pump inhibitors (PPI). I guess it could be acute coronary syndrome (ACS), we should get an EKG and chest X-ray.

## Appendix 2f One Minute Preceptor Scenario 1: Resident Script

Setting: You are an EM resident paired with a medical student in the ED. The scenario should begin with the learner presenting their assessment and plan.

Patient: 57-year-old Male with Chest Pain

HPI: Pain began while cleaning out his garage 2 hours ago. Rates it a 7/10 in severity. Radiates into the left jaw/arm. Described as a tightness. No aspirin (ASA) or nitro before. Patient believes it is gastroesophageal reflux disease (GERD).

Past medical history: hypertension (HTN), diabetes mellitus (DM)

Medications: Lisinopril, Metformin

Family history: coronary artery disease (CAD)

Social history: Daily Smoker

Physical exam: Unremarkable

## Appendix 2g One Minute Preceptor Scenario 2: Medical Student Script

Setting: You are an overconfident fourth-year medical student on your EM rotation. Please begin with presenting the assessment and plan.

Patient: 25-year-old female with right-sided abdominal pain

HPI: 3 days of worsening right lower quadrant (RLQ) pain that is now severe. Associated with fever, nausea and vomiting. Spotty birth control compliance and is having vaginal bleeding. Past medical history: Previously healthy

Physical exam: Febrile uncomfortable appearing female. Significant right lower abdominal tenderness to palpation, but non-distended. Tachycardic.

**Your Assessment & Plan:** This is a 25-year-old female with right sided abdominal pain. Differential includes kidney stone vs. obstruction vs. biliary disease vs. appendicitis. We should probably treat her pain, get her some fluids and labs, and probably get some imaging.

## Appendix 2h One Minute Preceptor Scenario 2: Resident Script

Setting: You are an EM resident paired with a medical student in the ED. The scenario should begin with the learner presenting their assessment and plan.

Patient: 25-year-old female with right-sided abdominal pain

HPI: 3 days of worsening right lower quadrant (RLQ) pain that is now severe. Associated with fever, nausea and vomiting. Spotty birth control compliance and is having vaginal bleeding. Past medical history: Previously healthy

Physical exam: Febrile uncomfortable appearing female. Significant right lower abdominal tenderness to palpation, but non-distended. Tachycardic.

## Appendix 2i One Minute Preceptor Scenario 3: Medical Student Script

Setting: You are a third-year medical student on your first EM shift, and you aren’t really sure what to do. Please begin with presenting the assessment and plan.

Patient: 14-year-old male with dyspnea

HPI: Symptoms began acutely while in gym class. Coughing fits are nonproductive. Scant response with inhaler. He denies any other accompanying or preceding symptoms.

Past medical history: Asthma

Medications: Albuterol Inhaler

Physical exam: Tachycardic. Labored breathing with obvious wheeze. Lung fields are significantly diminished bilaterally. He can only speak 2–3 words at a time.

**Your Assessment & Plan:** This is a 14-year-old male presenting with dyspnea. He is having significant respiratory distress. I will treat this with albuterol and plan on getting a Chest X-ray and a full set of labs.

## Appendix 2j One Minute Preceptor Scenario 3: Resident Script

Setting: You are an EM resident paired with a medical student in the ED. The scenario should begin with the learner presenting their assessment and plan.

Patient: 14-year-old male with dyspnea

HPI: Symptoms began acutely while in gym class. Coughing fits are nonproductive. Scant response with inhaler. He denies any other accompanying or preceding symptoms.

Past medical history: Asthma

Medications: Albuterol Inhaler

Physical exam: Tachycardic. Labored breathing with obvious wheeze. Lung fields are significantly diminished bilaterally. He can only speak 2–3 words at a time.

## Appendix 3a Feedback Session Facilitator Guide

### Objectives

Following completion of this session, residents will be able to:

- Recognize and define the aspects of effective feedback.
- Apply the aspects of effective feedback on shift while providing feedback to the medical students.
- Describe the common pitfalls of feedback.

### Pre-Session Preparation

- It is strongly recommended that the session handouts and the scenario scripts are loaded into a GoogleDrive folder. These can then be linked to the scenario scripts into the bottom of the handouts for ease of access.
- Ensure that sharing permissions are correct (eg, in Google Drive, the option, “everyone with the link can view,” is selected under the “Share” options).

### Roles

- *Lecturer*: Will provide the main lecture for the session and can also provide the recap on the previous session. Alternatively, you can choose a second lecturer to provide the recap from the previous session.
- *Video Conferencing Facilitator:* Recommend that this person be separate from the facilitators for the small groups. They will assign participants to the break out rooms, move Small Group Facilitators between the breakout rooms, and keep time for the breakout sessions. Also recommend that this person have access to the handout and scenario script links to send out in the video conferencing chat.
- *Small Group Facilitators*: Will also likely include the lecturer. Depending on the number of small groups, these facilitators can stay with one group or be moved through different groups by the Video Conferencing Facilitator. These facilitators should be experienced educators who are prepared to answer questions regarding the topics presented in the lecture so that they can efficiently guide the scenarios and participate in the debriefs.

### Timing Outline

0000–0005: Recap previous session

0005–0025: Lecture on Feedback

0025: Separate into small groups

0025–0030: 1st role-playing scenario

0030: Return to large group

0030–0035: Debrief scenario 1

0035: Separate into small groups

0035–0040: 2nd role-playing scenario

0040: Return to large group

0040–0045: Debrief role-playing 2

0045: Separate into small groups

0045–0050: Role-playing scenario 3

0050: Return to large group

0050–0055: Debrief role-playing scenario 3

0055: Final comments and end session

The timing once the role-playing scenarios start is more fluid and is also dependent on how the learners work through the scenarios. Recommend communicating back to the Video Conferencing Facilitator to end the role-playing scenarios as groups finish to start the debriefing discussions.

### Content Outline

#### Lecture (20 min)

- Examples of bad feedback - can solicit more of these from the audience or substitute in local examples if desired.
- Bad feedback video - audience will have no trouble coming up with why the feedback in this video is bad. Consider asking them to also think of examples of what is *good* about this feedback session.
- Characteristics of effective feedback - feel free to pepper with personal examples that emphasize these points.
- Coaching model - can also expound on important aspects of the coach-player metaphor that may be relevant to feedback - mutual respect, helping the learner “perfect their craft,” “achieve peak performance,” improving on one thing per day.
- If R2C2 model is confusing, can offer another example.

#### Role-playing scenarios

- Groups can be 2 or 3 residents, with one playing the student, one playing the resident, and an optional observer (in the case of groups of 3).
- The third case can be considered optional if the discussion of the first two scenarios runs long.
- Ideally, there should be enough scenarios to allow each participant to play each role at least once.
- Ensure that all participants have access to the handout to reference prior to the start of the role-playing scenarios.
- Provide scripts for the student and resident portions of each scenario via links.
- Recommend including these in the handout reference.
- If desired, can direct the observer (if present) to look at specific aspects of the interaction (eg, one thing that was done really well and one thing that could be improved).

#### Debrief

- Elicit emotional response to the case:
- Example: Did it remind them of difficult interactions in the past?
- Solicit examples from “learners” about what they thought went well in their interaction that others could emulate.
- Ask those providing feedback what was difficult and solicit ideas from the group for how to overcome.
- Look for and emphasize examples of coaching, soliciting perspectives from those in the medical student role.
- Address any knowledge gaps and correct assumptions.
- Summarize 1–2 important take-aways from the case and discussion.

### References

- Ende J. Feedback in Clinical Medical Education. *JAMA* 1983;250(6):777–81.
- Weinholz D. *Teaching During Attending Rounds: A Manual for Attending Physicians*. Iowa City: University of Iowa Office of Consultation and Research in Medical Education, 1987.
- Brinko KT. The Practice of Giving Feedback to Improve Teaching: What is Effective? *J Higher Education* 1993; 64(5):574–93.
- Kaprielian VS, Gradison M. Effective Use of Feedback. *Family Medicine* 1998;30(6):405–6.
- Thurgur L, Bandiera G, Lee S, Tiberius R. What do emergency medicine learners want from their teachers? A multicenter focus group analysis. *Acad Emerg Med* 2005; 12(9):856–861.
- Sargeant J, Lockyer J, Mann K, et al. Facilitated Reflective Performance Feedback: Developing an Evidence- and Theory-Based Model That Builds Relationship, Explores Reactions and Content, and Coaches for Performance Change (R2C2). *Acad Med*. 2015;90(12):1698–1706. doi:10.1097/ACM.0000000000000809

## Appendix 3b Providing Effective Feedback Handout

### Elements of *effective* feedback

- TIMELY
- SPECIFIC
- EMOTIONALLY NEUTRAL
- DESCRIPTIVE
- ACTIONABLE

#### How to prepare learners for feedback

- **Learners need to have a goal:** Ask learners what they want to improve on. If learners don’t have a goal, help them brainstorm and select one.
- **Set a specific place & time:** Often learners don’t realize they are getting feedback unless you specifically tell them, “I have some feedback on your clinical performance today.” Although not always practical, it may help to relocate to a non-clinical area (eg, breakroom) which allows the learners to “shift gears” and prepare mentally to give/receive feedback.
- **Avoid humiliation:** Simply put, it promotes a culture of shame. If you have sensitive feedback that you think may trigger an emotional response, relocate to a private area.
- **Start with a self-assessment:** “What went well? What would you change for next time?”
- **There is no positive & negative feedback:** Rather there is effective and ineffective feedback. Effective feedback is specific, non-judgmental and actionable. Remember, the goal is to help the learner improve and grow. Ineffective feedback is vague (eg, “great job”) or judgmental (eg, “you really messed up that procedure”).

### Other considerations

- **Gender bias exists:** Women are more likely to receive subjective feedback as opposed to their male colleagues, especially in regards to their: **physical appearance, confidence and leadership ability**.
  ○ The successes of women are more often attributed to luck or hard work rather than innate ability/skills
  ○ **Examples of gendered vocabulary:**
    ▪ “Men are assertive, women are bossy/abrasive”
    ▪ “Men pay attention to detail, women are control freaks”
    ▪ “Men lose their tempers, women are emotional”

### Role-Playing Scenarios

- Scenario 1:
  - Resident Script: *insert link here*
  - Medical Student Script: *insert link here*
- Scenario 2:
  - Resident Script: *insert link here*
  - Medical Student Script: *insert link here*
- Scenario 3:
  - Resident Script: *insert link here*
  - Medical Student Script: *insert link here*

## Appendix 3c One Minute Preceptor Recap Lecture

Please see associated PowerPoint file

## Appendix 3d Feedback Lecture

Please see associated PowerPoint file

## Appendix 3e Feedback Example Scenario

This scenario was created to demonstrate quality feedback prior to the breakout role-playing sessions. Two small-group facilitators would be good options to act out these parts. We recommend giving them this script prior to the Feedback Session so that they can have a chance to review the content.

R - Resident, S - Medical Student

R: Now that we’re in a quiet space, I wanted to give you some feedback like I promised at the beginning of the shift.

S: Sounds good to me. How did I do?

R: Well, how do *you* think the shift went?

S: I think I did a pretty good job seeing a lot of patients today.

R: I agree. You saw a good volume and had some interesting cases. I think you did a great job of keeping a broad, but reasonable differential. It’s important not to anchor on a patient’s chief complaint in EM. Was there anything you felt like you could improve?

S: On the appendicitis patient, it took me a while to realize that we hadn’t put in pain medication orders for them until the nurse came and talked to me about it.

R: Yeah, it can be frustrating when we forget things. When things get busy in the ED, we have to rely on good communication with our team so nothing gets missed. I always try and circle back with the patient’s nurse after I’ve seen the patient to give them an idea of what we need to do next and what orders they may need. Maybe you could try that next shift and see if you feel like it makes things more efficient.

S: Yeah, that’s a good idea. I’ll try and work with the nurses more next time.

R: I think overall you are doing exactly what you need to do as a fourth-year medical student. I do want to challenge you to be more direct with your plans. Feel free to say what you think needs to be ordered, and don’t worry if it's right or wrong.

S: Aw man, I thought I did really well with my assessment and plan for my patients today.

R: I think you did a good job for where you are in your training, but there’s always room for improvement. For example, when I asked if the appendicitis patient needed imaging, make sure to defend your argument that they needed a scan. In this case, they certainly did!

S: I see, so be more direct and confident in my own plans. Got it!

R: Sounds great. I really enjoyed working with you. Make sure to finish your notes soon so I can cosign them. I’ll provide feedback on those too. See you next time!

S: Thank you so much!

## Appendix 3f Feedback Scenario 1: Medical Student Script

You have come to the end of a stretch of 5 shifts in a row, so you are feeling ready to leave the department and relax tomorrow. It was a busy day and by the time sign-out finished and you helped with dispositioning your last patient, it is already an hour after the shift was supposed to end. While this shift was certainly not your best work ever, you did a nice job taking care of a complex lac repair, and feel that it was a solid performance overall, and there were no major issues. Mostly, you’re not looking for any specific feedback and just want to get home.

## Appendix 3g Feedback Scenario 1: Resident Script

This is your last shift working with this student and you are concerned. Based on today’s shift, you think the student has some significant deficits with patients with neurologic complaints. You are not sure if the student has ever been told this before and want to make sure that the student knows additional studying is needed. Specifically, you thought that the student lacked some compassion dealing with the patient with suspected psychogenic nonepileptic seizures, as well as a systemic approach to the history taking and you would like to offer some advice about how to improve on the next shift.

## Appendix 3h Feedback Scenario 2: Medical Student Script

You are feeling good about your performance after a tough overnight shift. You are attempting to push yourself to see more patients, and though there were a few times when you felt overwhelmed by the volume, you think that overall, you did well – you nearly saw a personal best number of patients! Although you were a little unclear on the finer points of the management of diabetic ketoacidosis when asked by your resident, you felt that you were a little scattered due to the number of patients that you were carrying, and you promised yourself that you would read up on it more later tonight. You’d like to hear what your resident thought about your ability to see a good volume of patients.

## Appendix 3i Feedback Scenario 1: Resident Script

You are fairly pleased with your student’s performance on their overnight shift – the student seemed interested and engaged with the many interesting cases that came through and was helpful with procedures. However, you noticed that a few of your patients seemed confused about their disposition – they told you that their student said they would come back and talk to them but never did. Additionally, you caught that one of the student’s patients was told incorrect information about being transferred to another hospital – nothing major, but the patient felt confused. Otherwise, you thought your student did a nice job with patient care, swiftly recognizing a patient with diabetic ketoacidosis and demonstrating knowledge easily when you asked them follow-up questions. You are curious what the student’s perception of their shift was.

## Appendix 3j Feedback Scenario 3: Medical Student Script

You are very interested in pursuing a career in emergency medicine, so you were trying very hard on your busy Monday afternoon shift. Previously, you have been told that your presentations are too long, so you are trying to work on shortening them up to impress your resident. You feel that you have made strong progress in this area during this shift, completing at least 2 presentations that you felt were high quality - concise but complete, while managing to stay on top of your other patient care responsibilities. You are curious as to what your resident thinks, since the resident still seemed somewhat dissatisfied when interacting with you during the shift, but did not give you any specific feedback thus far. You are hoping that your perception is wrong, however, since you want to make sure your reputation in the emergency department is stellar.

## Appendix 3k Feedback Scenario 3: Resident Script

You are feeling drained after the end of a very busy shift. Several complex resuscitations took up a great deal of your time and you just finished a challenging interaction with a consultant. You were also quite annoyed by the performance of your medical student, who seemed to disappear for hours at a time during the shift, missing several interesting resuscitations that a student supposedly interested in emergency medicine should have been present for. Additionally, when he was around, he seemed overly casual with the residents and often off task – pulling up YouTube videos on the work computers and Snapchat pictures of his most recent vacation. While you feel his presentations have improved somewhat from the beginning of your string of shifts together, you feel they are still overly detailed and show very little evidence that he is thinking about dangerous diagnoses and how to work them up. However, you feel that these concerns are far outweighed by the student’s overall lack of professionalism on shift.

## Appendix 4a Tips and Tricks Session Facilitator Guide

### Objectives

Following completion of this session, residents will be able to:

- Describe multiple on-shift learning tools to utilize during their paired shifts.
- Incorporate into performance multiple tips and tricks taught in this lecture.
- Design two brief lectures to give to medical students on shift.

### Pre-Session Preparation

- It is strongly recommended that the session handouts and the scenario scripts are loaded into a GoogleDrive folder. These can then be linked to the scenario scripts into the bottom of the handouts for ease of access.
- Ensure that sharing permissions are correct (eg, in Google Drive, the option, “everyone with the link can view,” is selected under the “Share” options).

### Roles

- *Lecturer*: Will provide the main lecture for the session and can also provide the recap on the previous session. Alternatively, you can choose a second lecturer to provide the recap from the previous session.
- *Video Conferencing Facilitator:* Recommend that this person be separate from the facilitators for the small groups. They will assign participants to the break out rooms, move Small Group Facilitators between the breakout rooms, and keep time for the breakout sessions. Also recommend that this person have access to the handout and scenario script links to send out in the video conferencing chat.
- *Small Group Facilitators*: Will also likely include the lecturer. Depending on the number of small groups, these facilitators can stay with one group or be moved through different groups by the Video Conferencing Facilitator. These facilitators should be experienced educators who are prepared to answer questions regarding the topics presented in the lecture so that they can efficiently guide the scenarios and participate in the debriefs.

### Timing Outline

0000–0005: Recap previous session

0005–0025: Lecture on Feedback

0025: Separate into small groups

0025–0030: 1st role-playing scenario

0030: Return to large group

0030–0035: Debrief scenario 1

0035: Separate into small groups

0035–0040: 2nd role-playing scenario

0040: Return to large group

0040–0045: Debrief role-playing 2

0045: Separate into small groups

0045–0050: Role-playing scenario 3

0050: Return to large group

0050–0055: Debrief role-playing scenario 3

0055: Final comments and end session

The timing once the role-playing scenarios start is more fluid and is also dependent on how the learners work through the scenarios. Recommend communicating back to the Video Conferencing Facilitator to end the role-playing scenarios as groups finish to start the debriefing discussions.

### Content Outline

#### Lecture

- Reviews the following teaching tools
  - “What if?” Game
  - Aunt Minnie
  - SPIT
  - Activated Demonstration
  - Self-directed teaching tools
  - Teaching scripts/mini lectures
  - Post-it Pearls

#### Role-playing scenarios

- Groups can be 2 or 3 residents, with one playing the student, one playing the resident, and an optional observer (in the case of groups of 3).
- The third case can be considered optional if the discussion of the first two scenarios runs long.
- Ideally, there should be enough scenarios to allow each participant to play each role at least once.
- Ensure that all participants have access to the handout to reference prior to the start of the role-playing scenarios.
- Provide scripts for the student and resident portions of each scenario via links.
  - Recommend including these in the handout reference.
- If desired, can direct the observer (if present) to look at specific aspects of the interaction (eg, one thing that was done really well and one thing that could be improved).

#### Debrief

- Elicit emotional response from the case.
  - Examples: What was difficult about choosing a teaching strategy on the spot? How does it feel to correct the “medical student” if they made mistakes?
- Discuss which teaching strategy the resident chose and why.
- Discuss any difficulties that may have occurred with the chosen teaching strategy.
- Discuss how the scenario felt from the “medical student’s” perspective.
  - Examples: Did the strategy feel like an effective way to learn? If you required correction, how did it feel?
- Address any knowledge gaps and correct assumptions.

### References

- Chinai SA, Guth T, Lovell E, Epter M. Taking Advantage of the Teachable Moment: A Review of Learner-Centered Clinical Teaching Models. *West J Emerg Med*. 2018;19(1):28–34. doi:10.5811/westjem.2017.8.35277
- Irby DM, Wilkerson L. Teaching when time is limited. *BMJ*. 2008;336(7640):384–387. doi:10.1136/bmj.39456.727199.AD
- Kaminsky A, Gallegos M, Liu J. Teaching on the Fly. *EMRA*Cast*. 2019. Accessed August 20, 2020. Available at: https://www.emra.org/about-emra/publications/emra-cast/teaching-on-the-fly/
- Swaminathan A. Post It Pearls 10.0. Core EM Website. August 18, 2017. Accessed September 27, 2020. https://coreem.net/blog/post-it-pearls/post-it-pearls-10-0/

## Appendix 4b Tips and Tricks Handout

### Self-directed Teaching Tool

- Create a worksheet-type form for the student to fill out
- Give them a specific question to look up to answer

### “What if” game

- New laboratory or imaging results
- New sign on physical exam
- New Symptom
- Change their home medications
- Give the patient a new previous medical problem
- Change the patient’s vital signs
- Give them a medication allergy

### Activated Demonstration

- A way to involve the medical student with more critical patients or procedures that are not appropriate for them to be performing
- Provide them with specific things to observe
- Discuss what they observed or questions they have afterwards

### Aunt Minnie Model

- Helps with rapid pattern recognition that we use in the ED
- Learner sees the patient and then presents only the chief complaint and the presumptive diagnosis
- You would then see the patient on your own and discuss the case with the student

### SPIT Model

- Model to discuss the patient with the medical student
- Serious, Probable, Interesting, Treatable

### Teaching Scripts

- Mini lectures to have on hand if things are slow

### Post-It Pearls

- Good for when you are busy because medical students can write down questions as they have them that you can revisit when you have time
- You can also pass on the information by posting the notes where people can see them

### Role-Playing Scenarios

- Scenario 1:
  - Resident Script: *insert link here*
  - Medical Student Script: *insert link here*
- Scenario 2:
  - Resident Script: *insert link here*
  - Medical Student Script: *insert link here*
- Scenario 3:
  - Resident Script: *insert link here*
  - Medical Student Script: *insert link here*

## Appendix 4c Feedback Recap Lecture

Please see associated PowerPoint file

## Appendix 4d Tips and Tricks for Teaching On-Shift Lecture

Please see associated PowerPoint file

## Appendix 4e Tips and Tricks Scenario 1: Medical Student Script

You are a third-year medical student on your second clinical rotation, and this is your fourth ED shift. The resident has sent you into a room to evaluate a 25-year-old female with a chief complaint of headache. You have seen a few patients on your shifts so far with similar presentations.

As you present this patient, feel free to express that you have seen a lot of patients like this before and you feel confident in treating this as a primary headache. As the resident leads you through the patient encounter discussion, please feel free to answer some questions incorrectly to give them a chance to practice correcting mistakes.

## Appendix 4f Tips and Tricks Scenario 1: Resident Script

You are paired with a third-year medical student who is early on in clinical rotations. The student worked three shifts so far on this rotation, but this is the student’s first rotation in an emergency department. You have sent the student to a room to examine a patient who is a 25-year-old female with a chief complaint of headache.

The medical student has returned and would like to present the patient to you. Please lead the medical student through the encounter utilizing one or more of the tips and tricks you were taught today.

## Appendix 4g Tips and Tricks Scenario 2: Medical Student Script

You are a third-year medical student who is about halfway through your rotations. This is your first shift in the emergency department and things appear to be going well.

Your resident has sent you in to see a 54-year-old female complaining of chest pain that started about 1 hour ago and worsened with exertion.

Please present this patient to your resident. As the resident leads you through the discussion, feel free to get some answers wrong to allow the resident the chance to practice correcting mistakes.

## Appendix 4h Tips and Tricks Scenario 2: Resident Script

You are paired with a third-year medical student who is about halfway through their rotations. You have sent the student in to see a 54-year-old female with chest pain for 1 hour that is worsened with exertion. You have already seen her EKG and it is not a STEMI.

Please lead your medical student through the discussion of this patient using one or more of the tips and tricks that you learned today.

## Appendix 4i Tips and Tricks Scenario 3: Medical Student Script

You are a third-year medical student on your emergency medicine rotation. This is your last clinical rotation. You were sent in to see a 55-year-old male presenting with left lower quadrant abdominal pain and constipation.

Please present this patient to the resident and allow the resident to lead you through the discussion of the patient. If the resident asks questions, feel free to answer some incorrectly to give the resident a chance to practice correcting mistakes.

## Appendix 4j Tips and Tricks Scenario 3: Resident Script

You are a resident working with a third-year medical student who is on the resident’s last clinical rotation of the year. The medical student has been in to see a patient with a chief complaint of abdominal pain.

The student will return to present the patient to you. Please lead the discussion around the patient encounter utilizing one or more of the tips and tricks that were discussed during this session.
