# Supplementary material for: A Novel Virtual Emergency Medicine Residents-as-Teachers (RAT) Curriculum
Source: J Educ Teach Emerg Med. 2021 Jul 15;6(3):C9–C63. doi: 10.21980/J86S71 (PMC10332683; doi:10.21980/J86S71)
Supplement: Supplementary file 1 — Please see associated PowerPoint file [file jetem-6-3-c8-Appendix2c.pptx]

## Slide 1
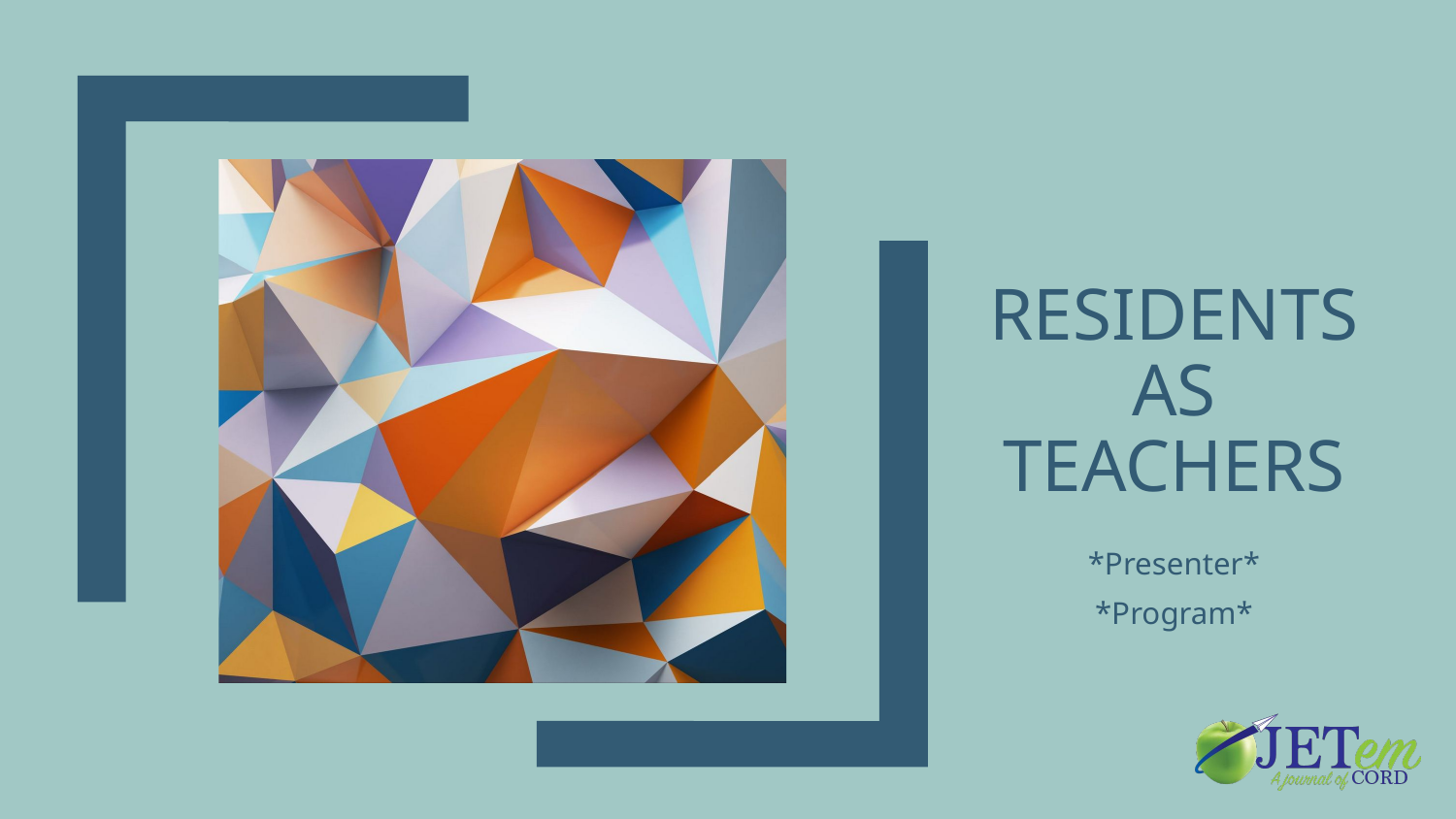

# Residents as Teachers
*Presenter*
*Program*

## Slide 2
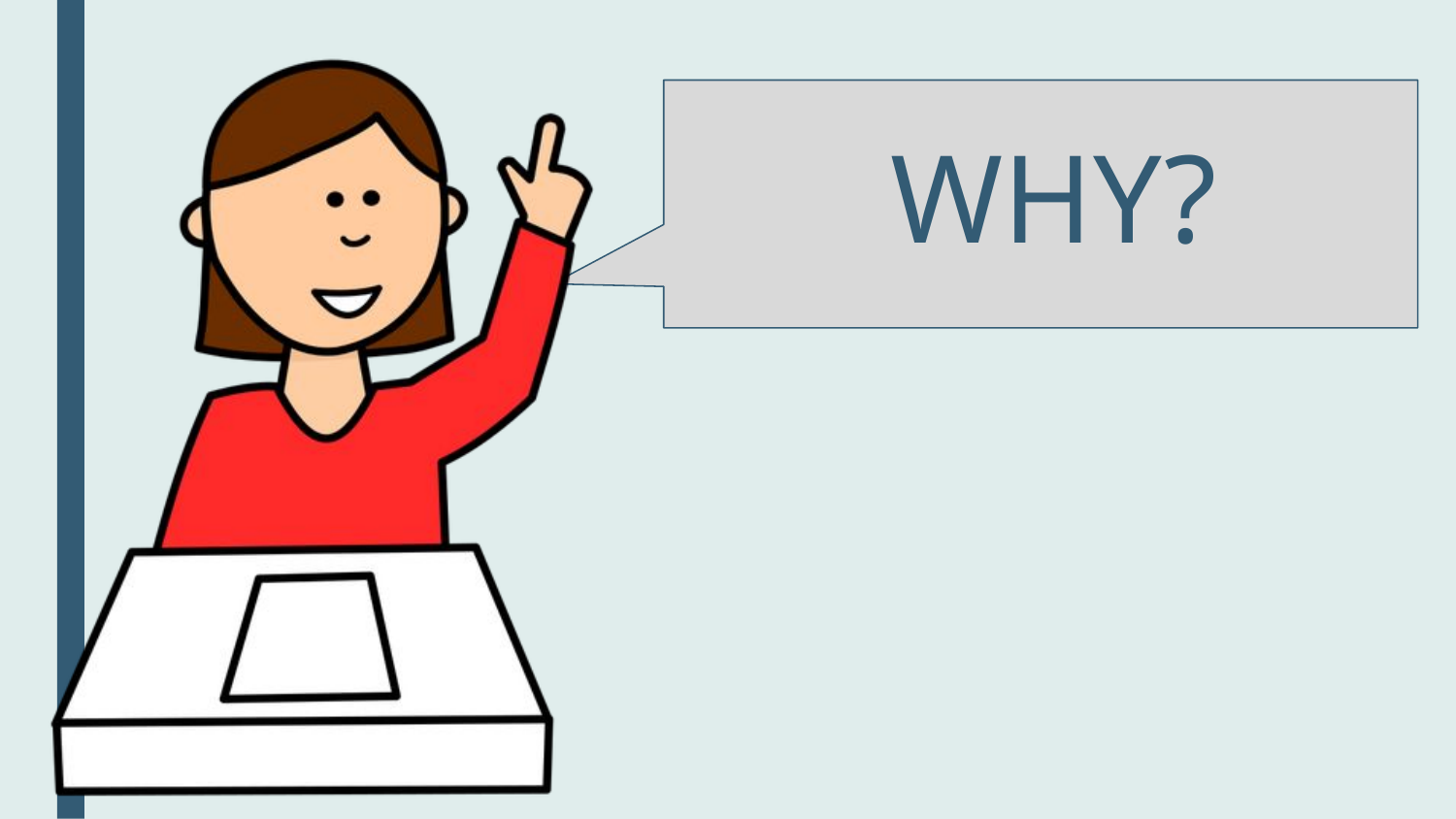

# WHY?

## Slide 3
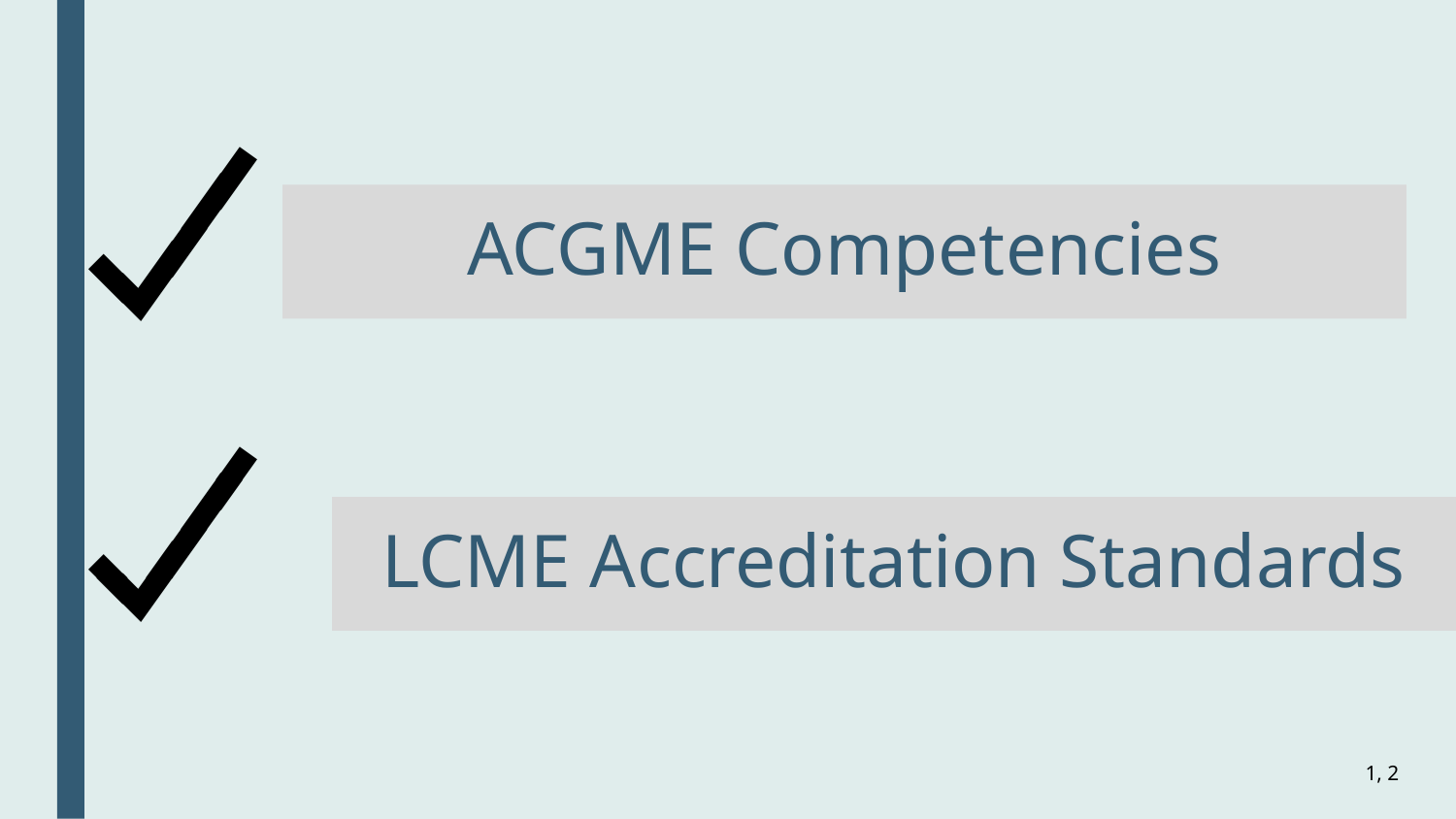

# ACGME Competencies
LCME Accreditation Standards
1, 2

## Slide 4
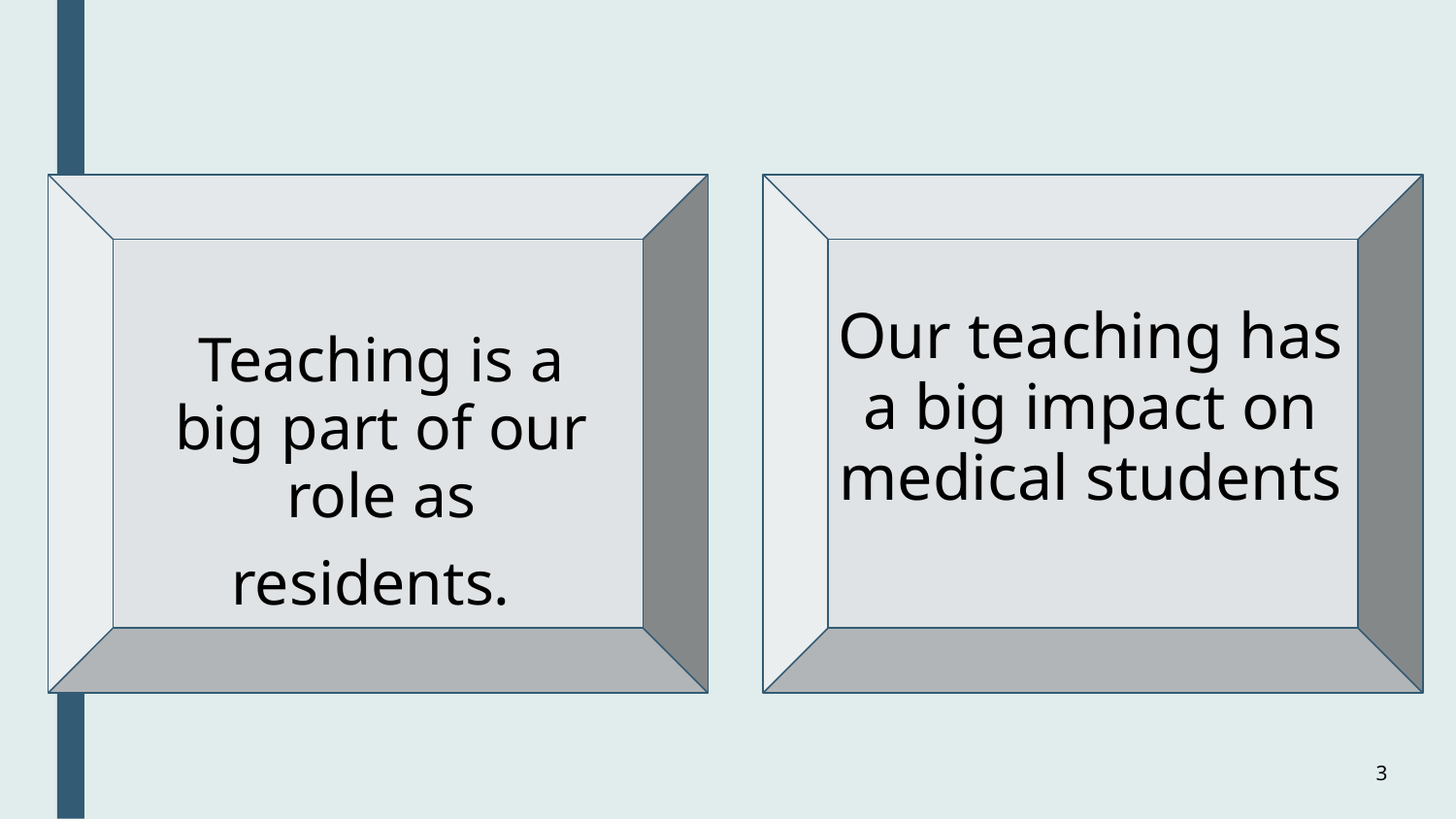

Our teaching has a big impact on medical students
Teaching is a big part of our role as residents.
 3

## Slide 5
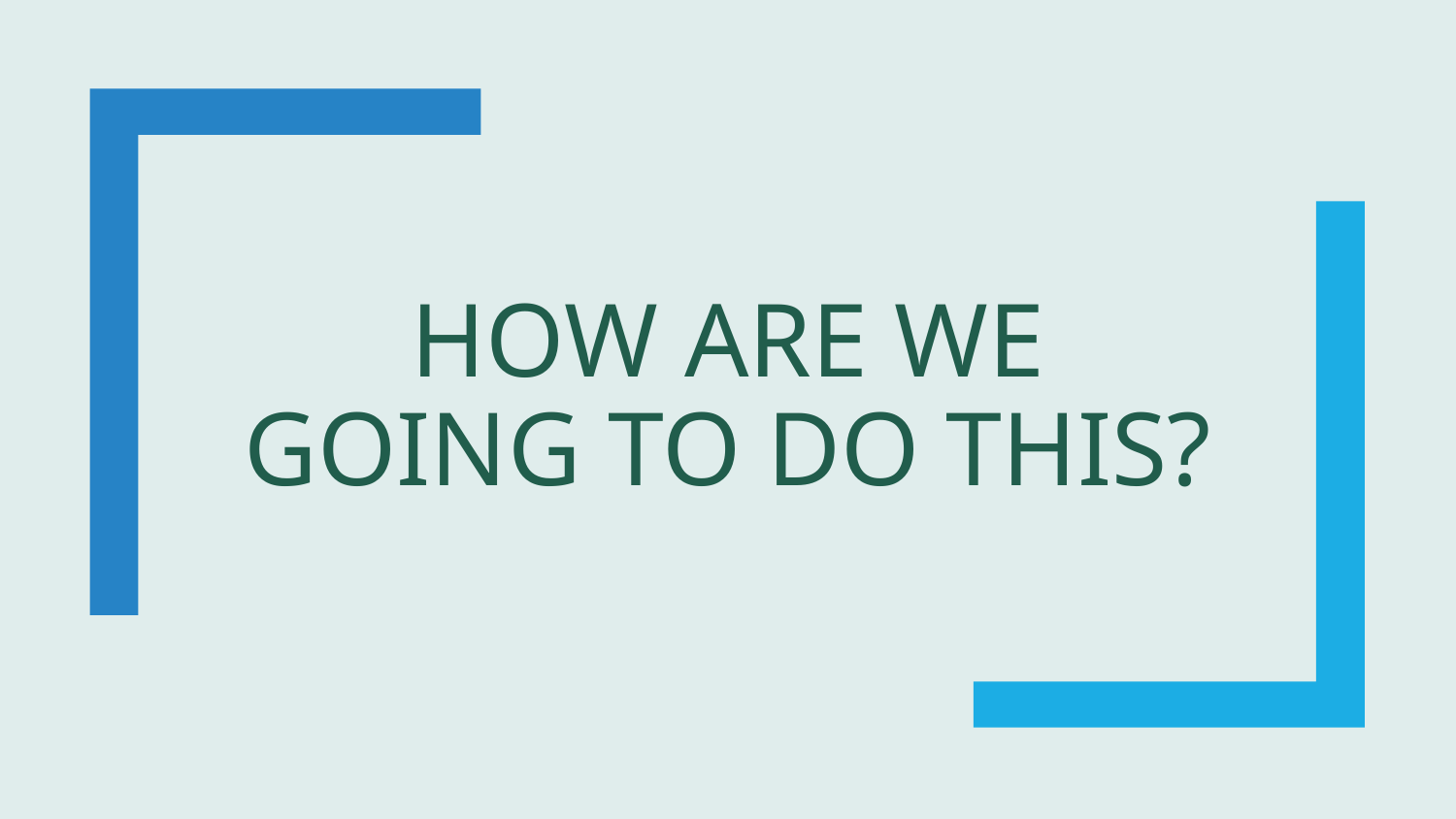

# How are we going to do this?

## Slide 6
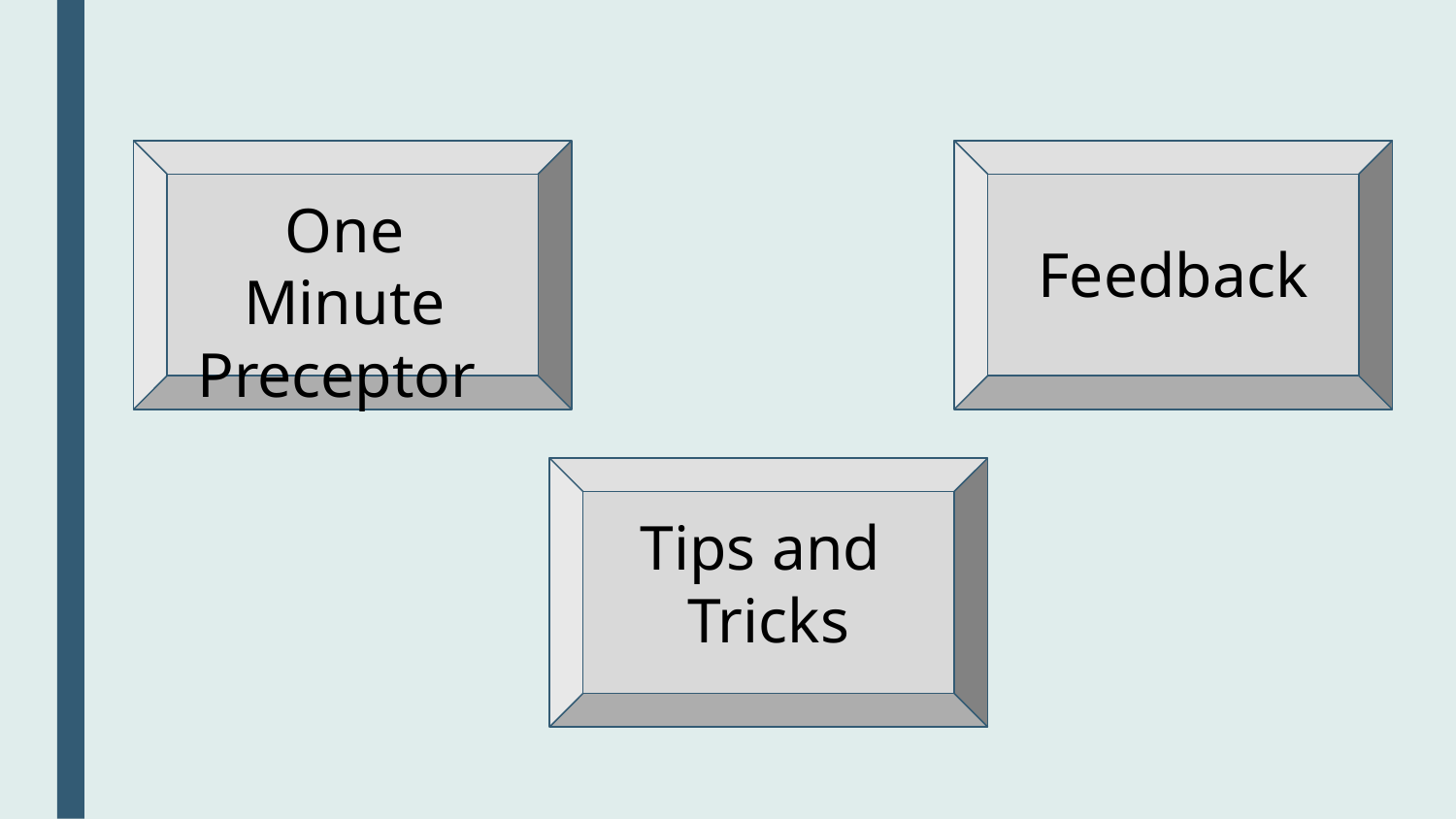

One Minute Preceptor
Feedback
Tips and
Tricks

## Slide 7
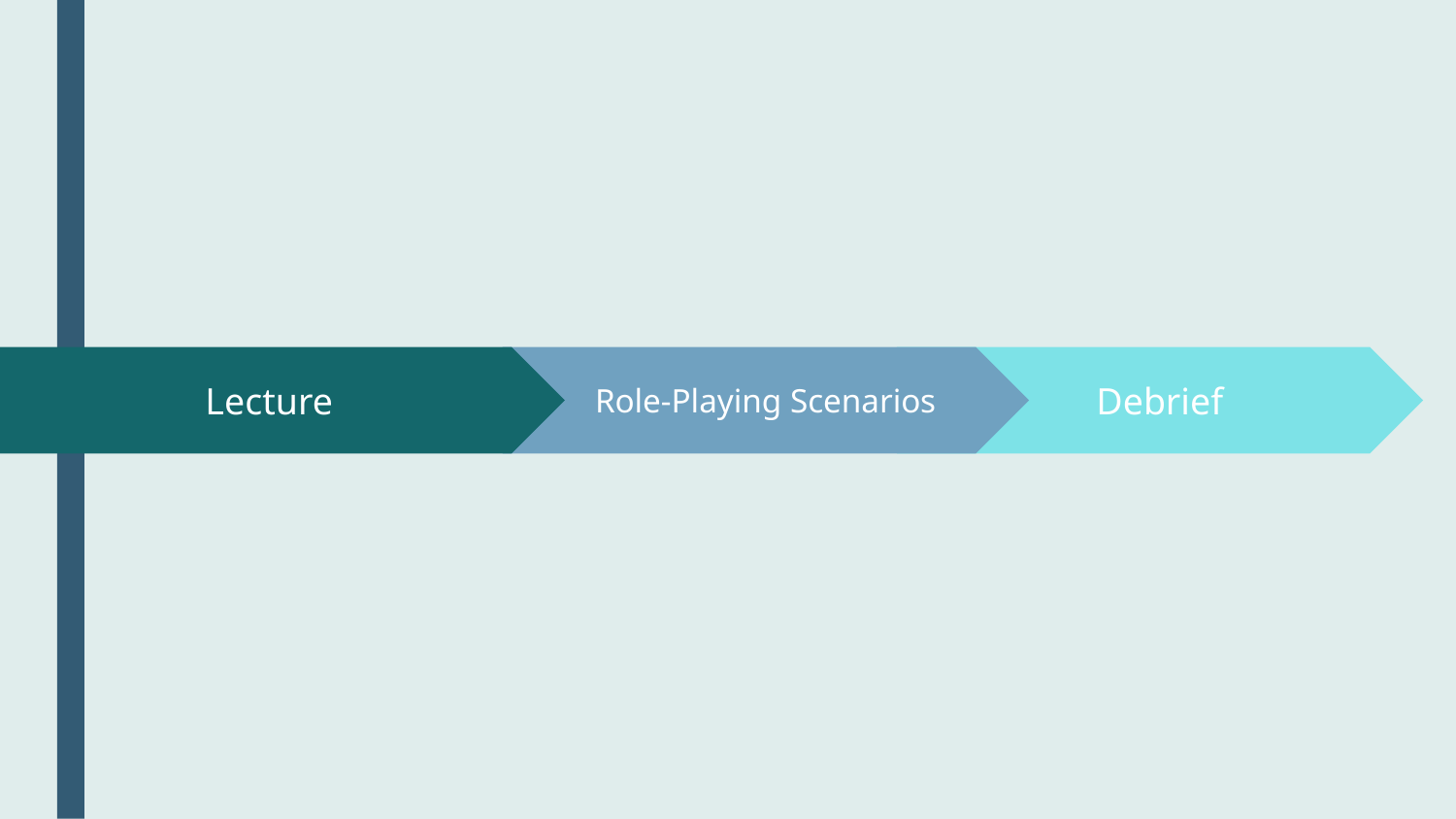

Role-Playing Scenarios
Debrief
Lecture

## Slide 8
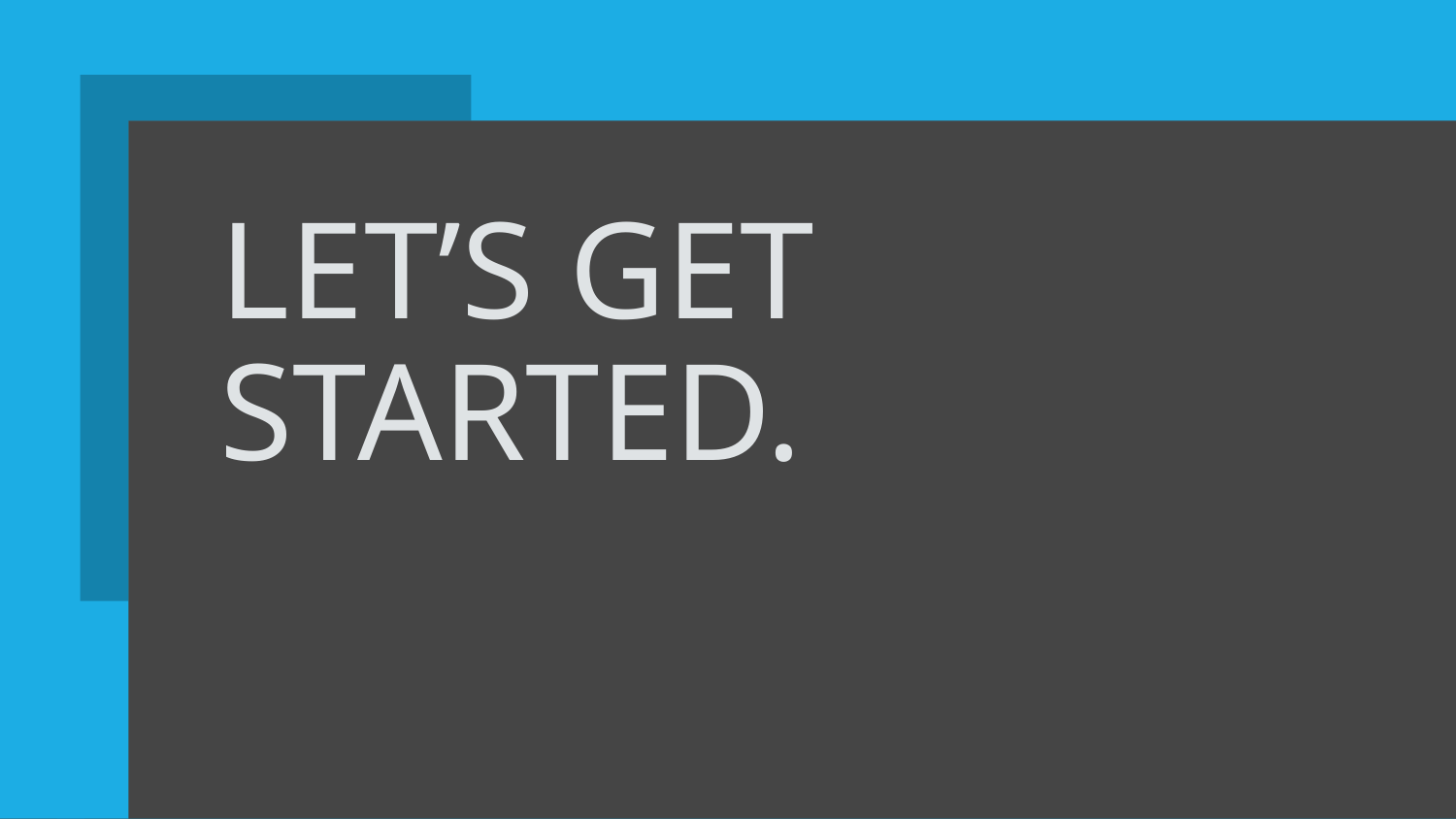

# Let’s get started.

## Slide 9
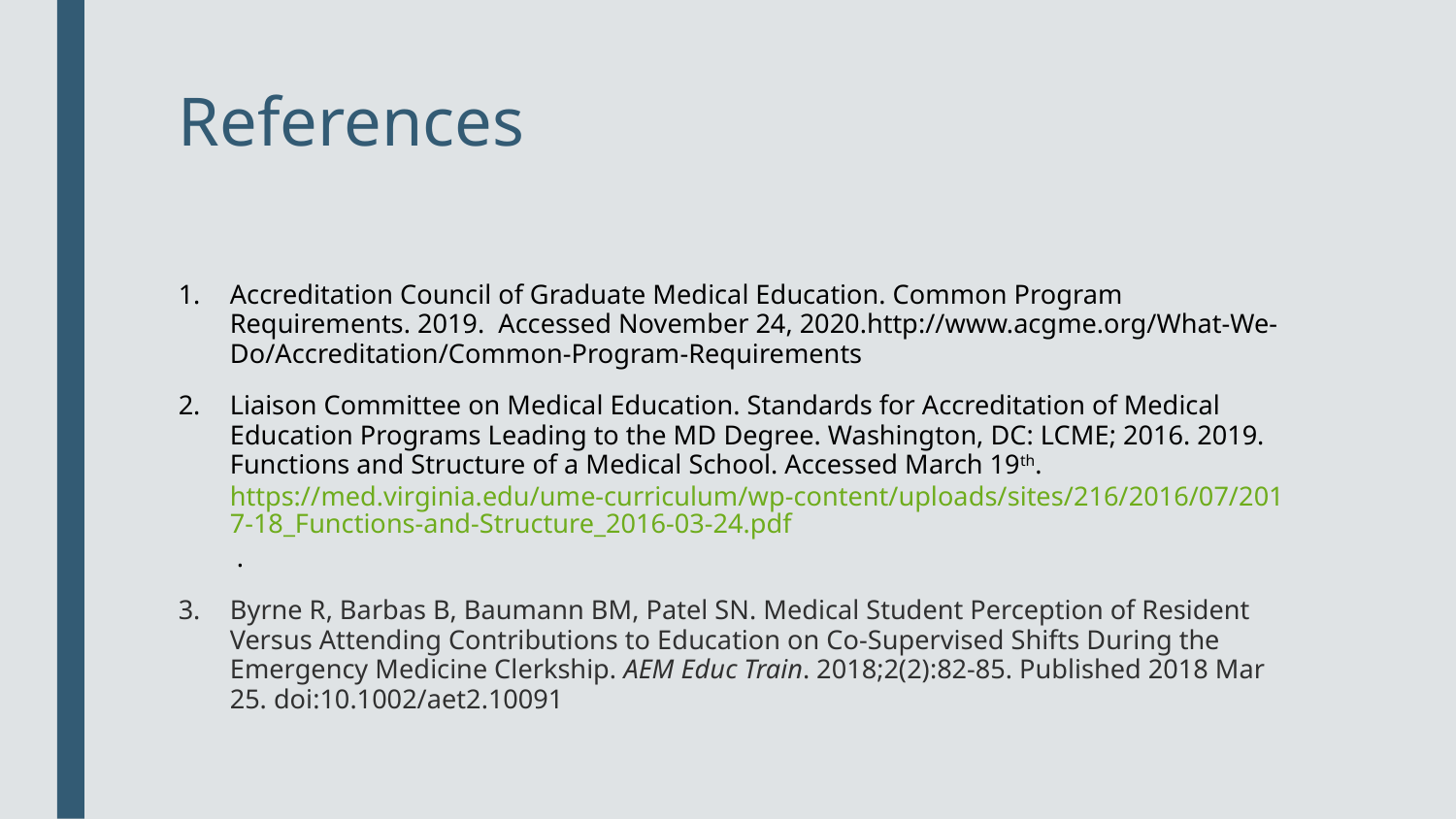

# References
Accreditation Council of Graduate Medical Education. Common Program Requirements. 2019. Accessed November 24, 2020.http://www.acgme.org/What-We-Do/Accreditation/Common-Program-Requirements
Liaison Committee on Medical Education. Standards for Accreditation of Medical Education Programs Leading to the MD Degree. Washington, DC: LCME; 2016. 2019. Functions and Structure of a Medical School. Accessed March 19th. https://med.virginia.edu/ume-curriculum/wp-content/uploads/sites/216/2016/07/2017-18_Functions-and-Structure_2016-03-24.pdf .
Byrne R, Barbas B, Baumann BM, Patel SN. Medical Student Perception of Resident Versus Attending Contributions to Education on Co-Supervised Shifts During the Emergency Medicine Clerkship. AEM Educ Train. 2018;2(2):82-85. Published 2018 Mar 25. doi:10.1002/aet2.10091
