# Supplementary material for: A Novel Virtual Emergency Medicine Residents-as-Teachers (RAT) Curriculum
Source: J Educ Teach Emerg Med. 2021 Jul 15;6(3):C9–C63. doi: 10.21980/J86S71 (PMC10332683; doi:10.21980/J86S71)
Supplement: Supplementary file 2 — Please see associated PowerPoint file [file jetem-6-3-c8-appendix2d.pptx]

## Slide 1
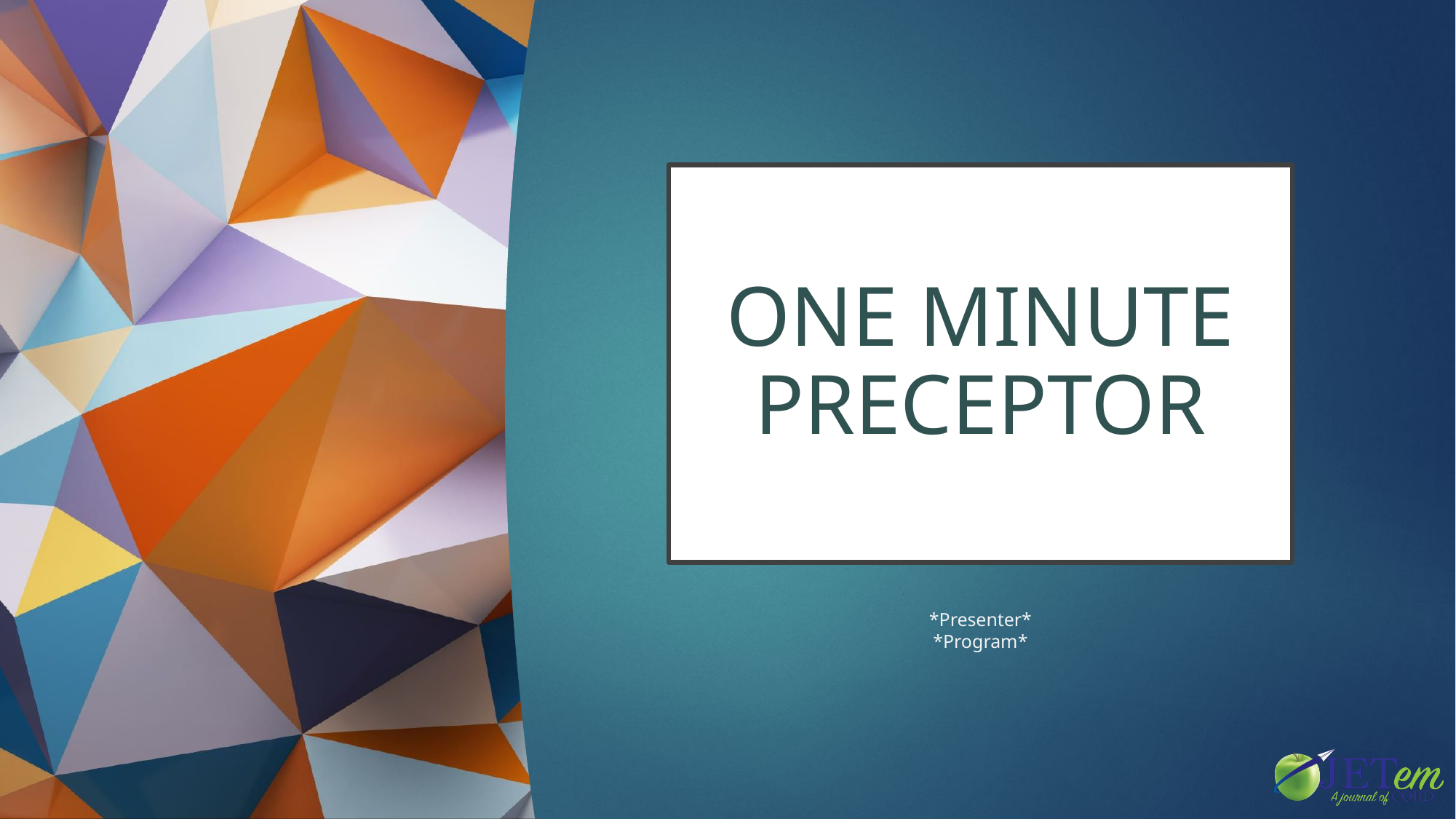

# ONE MINUTE PRECEPTOR
*Presenter*
*Program*

## Slide 2
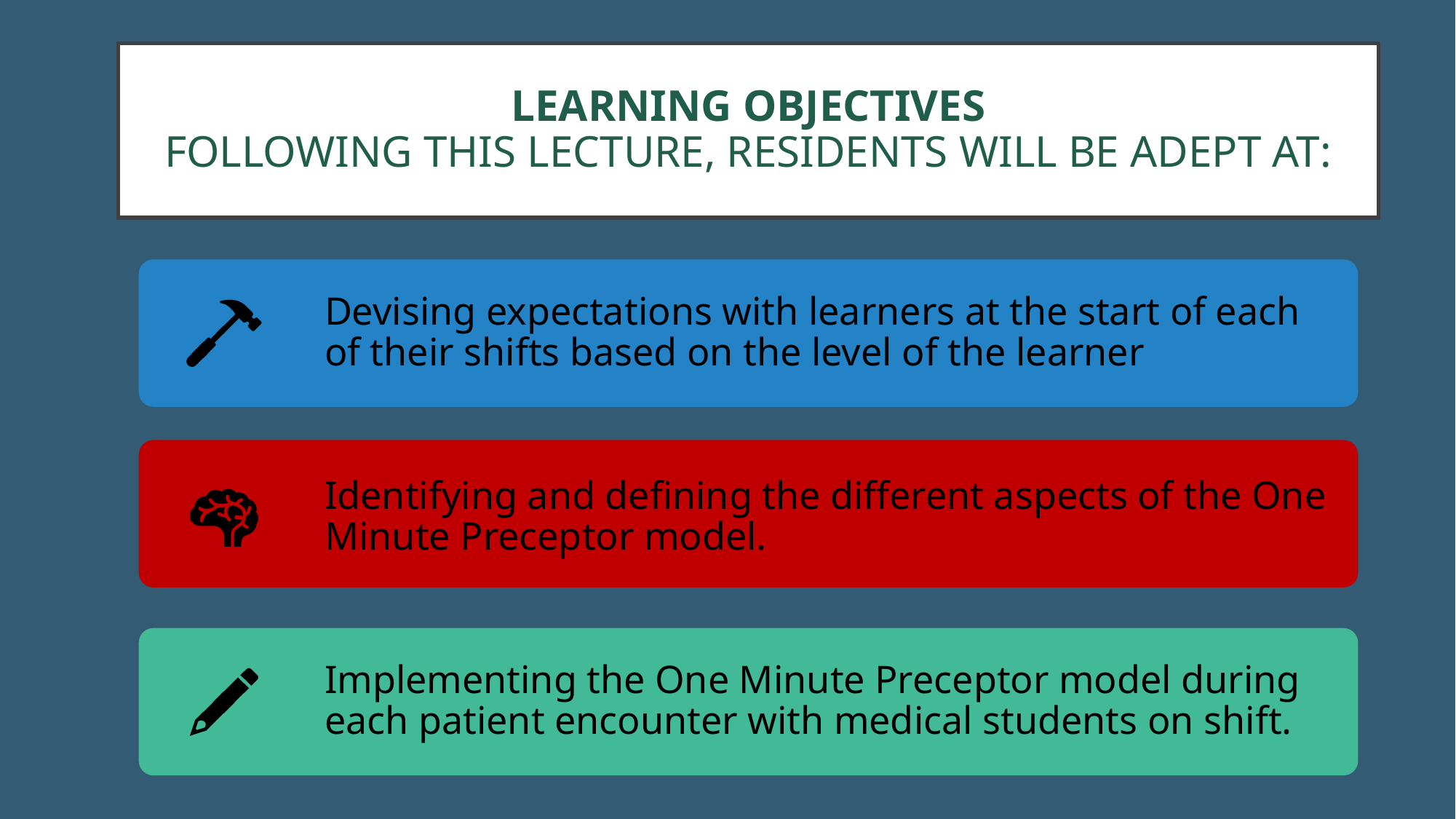

# LEARNING OBJECTIVES FOLLOWING THIS LECTURE, RESIDENTS WILL BE ADEPT AT:
Devising expectations with learners at the start of each of their shifts based on the level of the learner
Identifying and defining the different aspects of the One Minute Preceptor model.
Implementing the One Minute Preceptor model during each patient encounter with medical students on shift.

## Slide 3
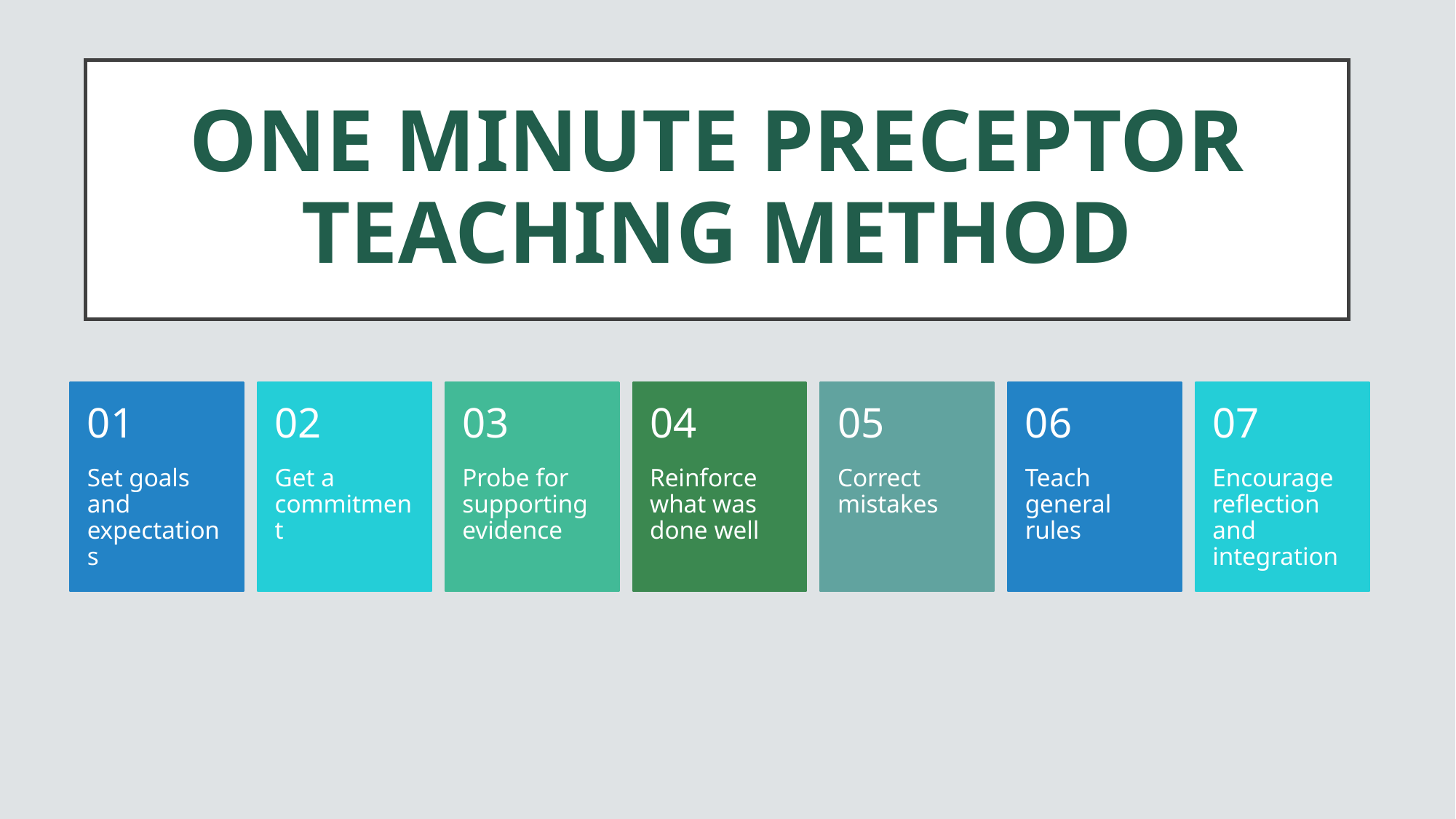

# ONE MINUTE PRECEPTOR TEACHING METHOD
01
02
03
04
05
06
07
Set goals and expectations
Get a commitment
Probe for supporting evidence
Reinforce what was done well
Correct mistakes
Teach general rules
Encourage reflection and integration

## Slide 4
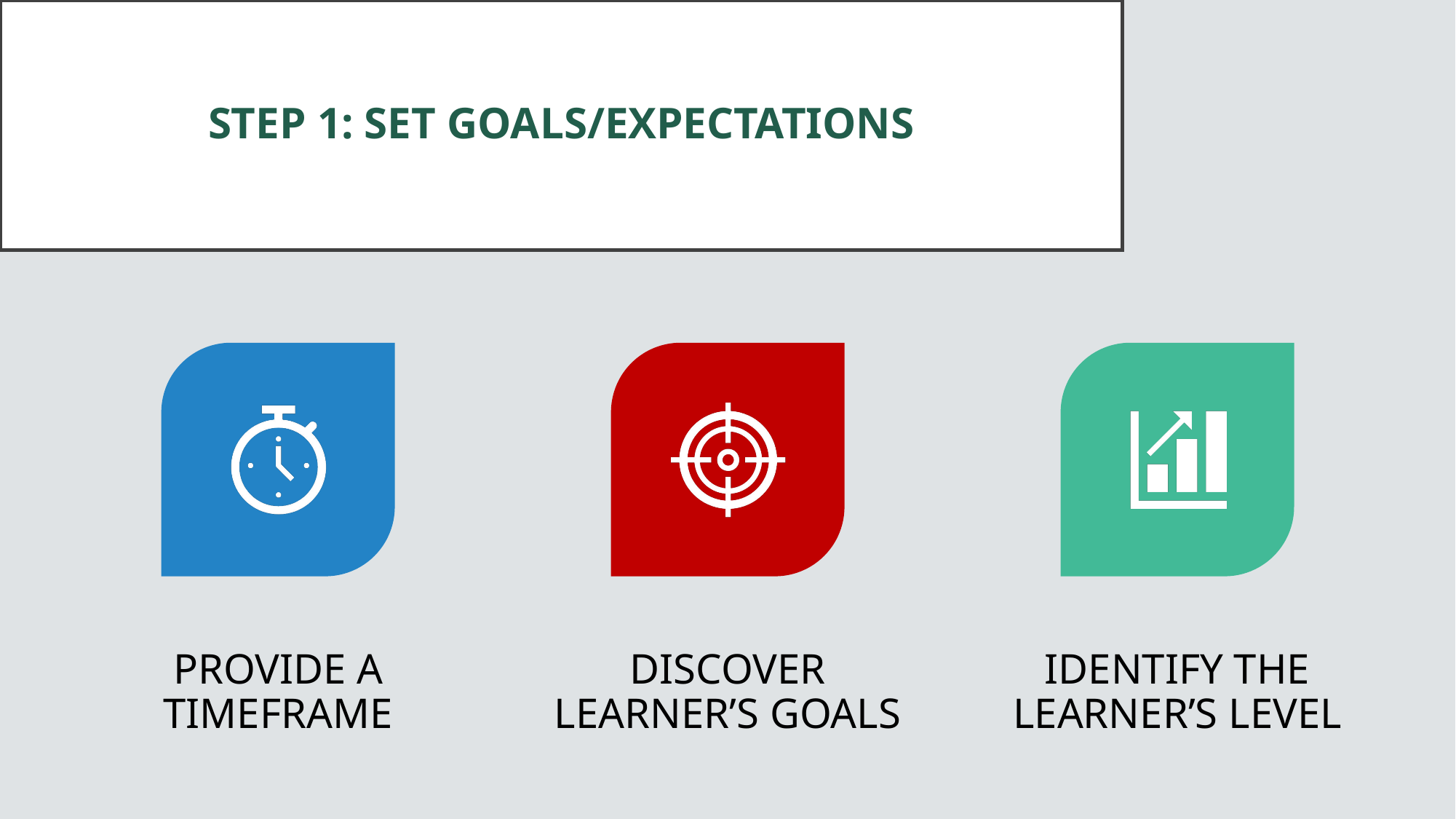

# STEP 1: SET GOALS/EXPECTATIONS
PROVIDE A TIMEFRAME
DISCOVER LEARNER’S GOALS
IDENTIFY THE LEARNER’S LEVEL

## Slide 5
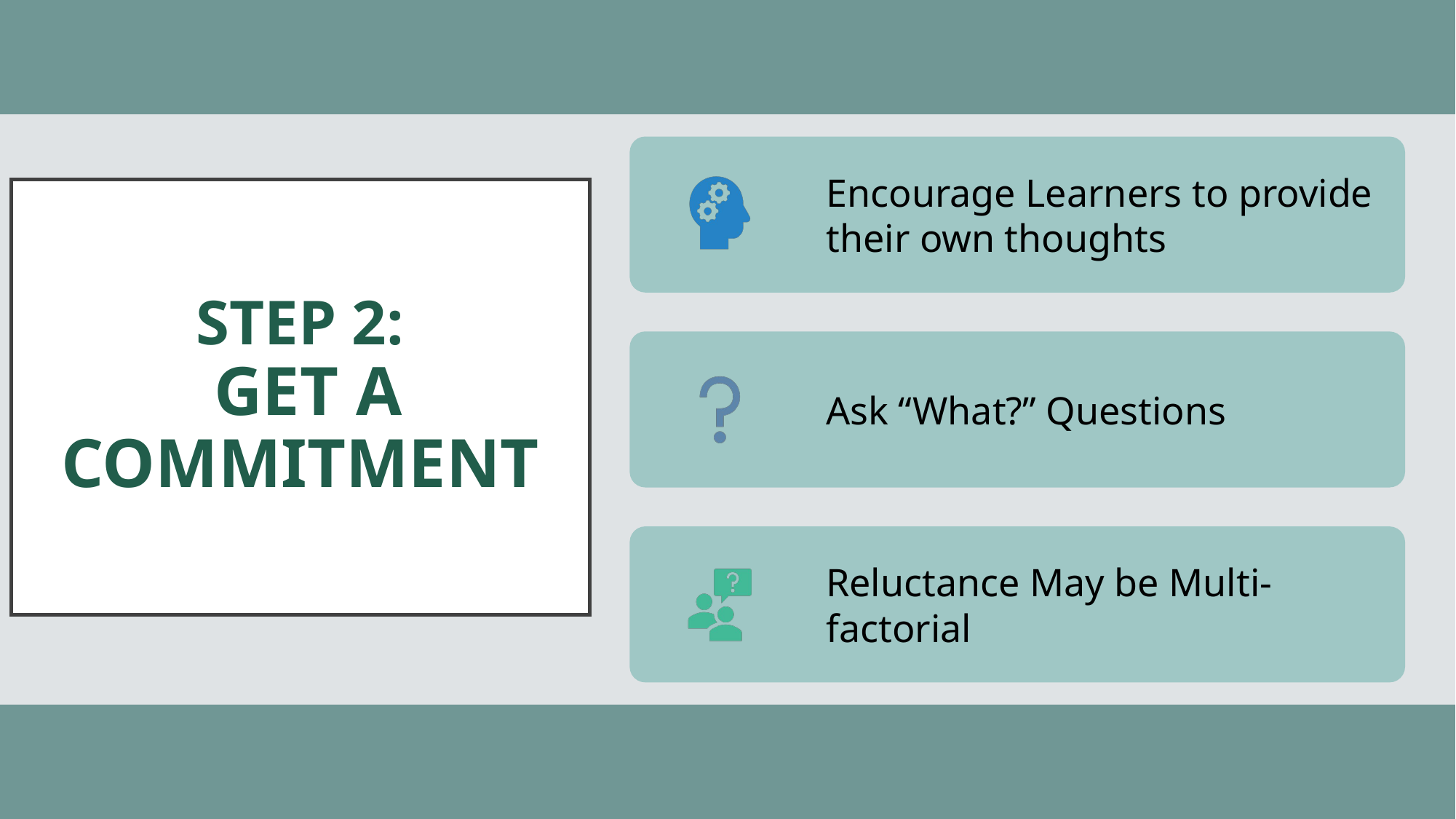

Encourage Learners to provide their own thoughts
Ask “What?” Questions
Reluctance May be Multi-factorial
# STEP 2: GET A COMMITMENT

## Slide 6
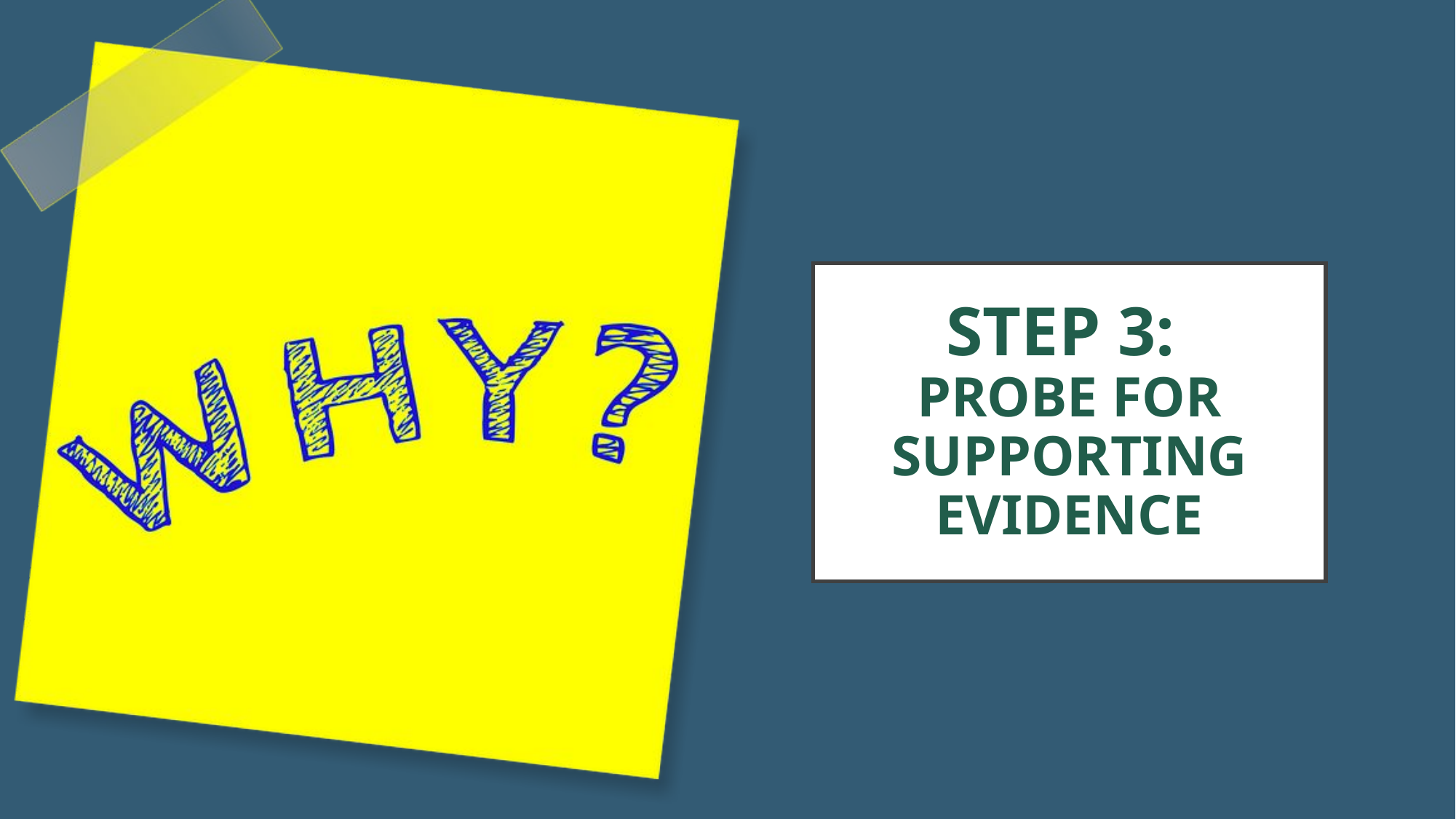

# STEP 3: PROBE FOR SUPPORTING EVIDENCE

## Slide 7
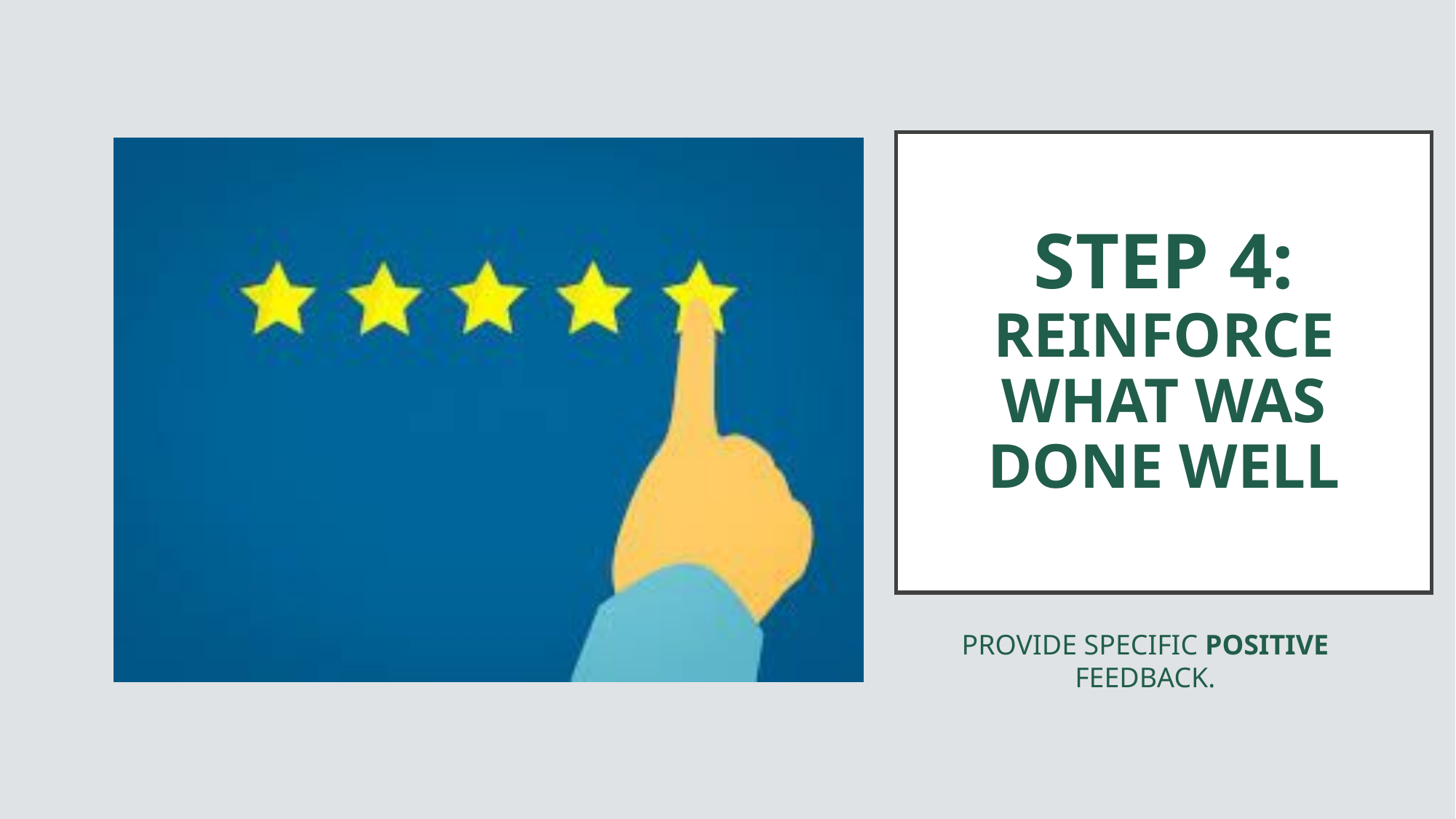

# STEP 4: REINFORCE WHAT WAS DONE WELL
PROVIDE SPECIFIC POSITIVE FEEDBACK.

## Slide 8
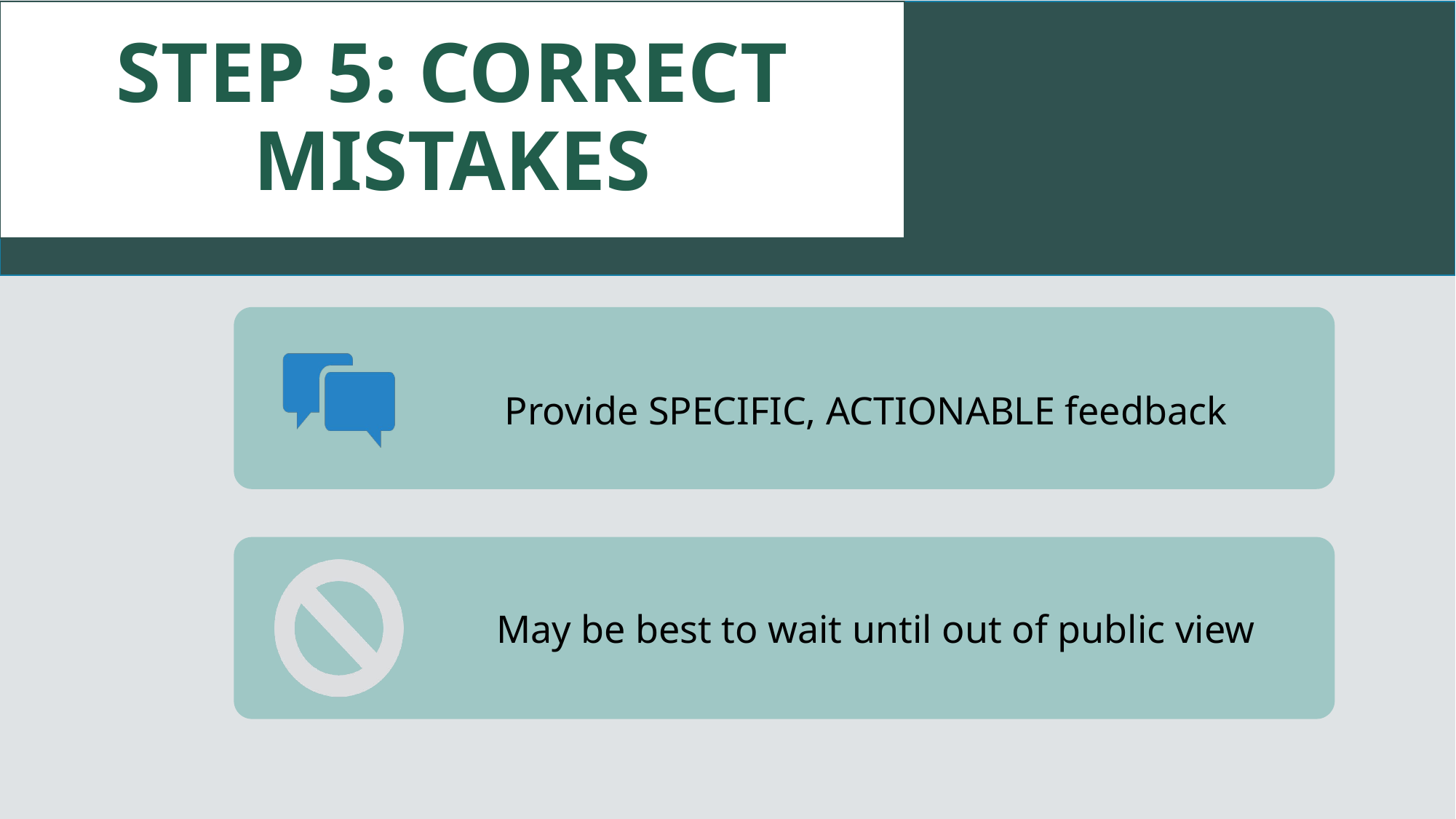

# STEP 5: CORRECT MISTAKES
Provide SPECIFIC, ACTIONABLE feedback
May be best to wait until out of public view

## Slide 9
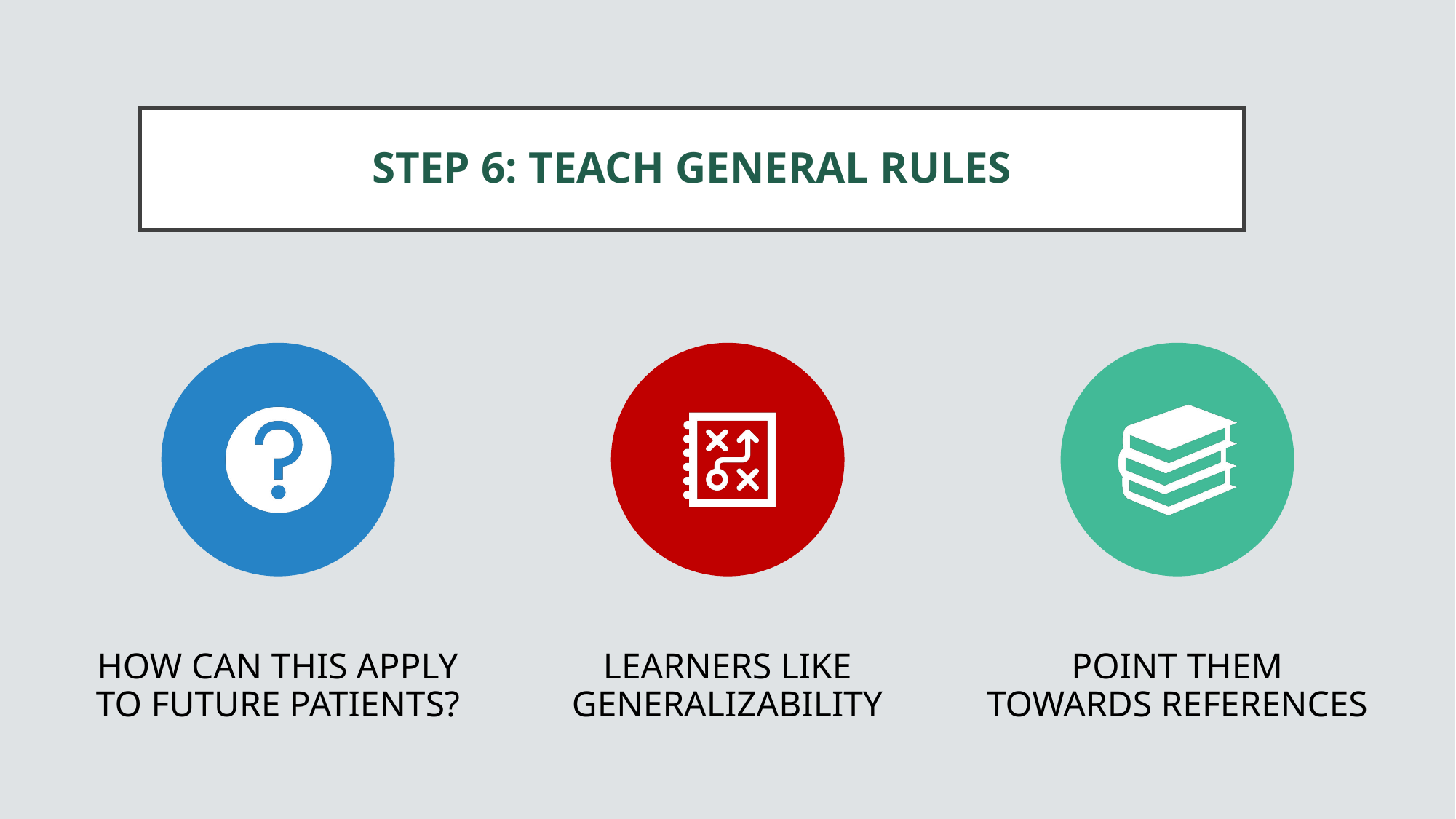

# STEP 6: TEACH GENERAL RULES
HOW CAN THIS APPLY TO FUTURE PATIENTS?
LEARNERS LIKE GENERALIZABILITY
POINT THEM TOWARDS REFERENCES

## Slide 10
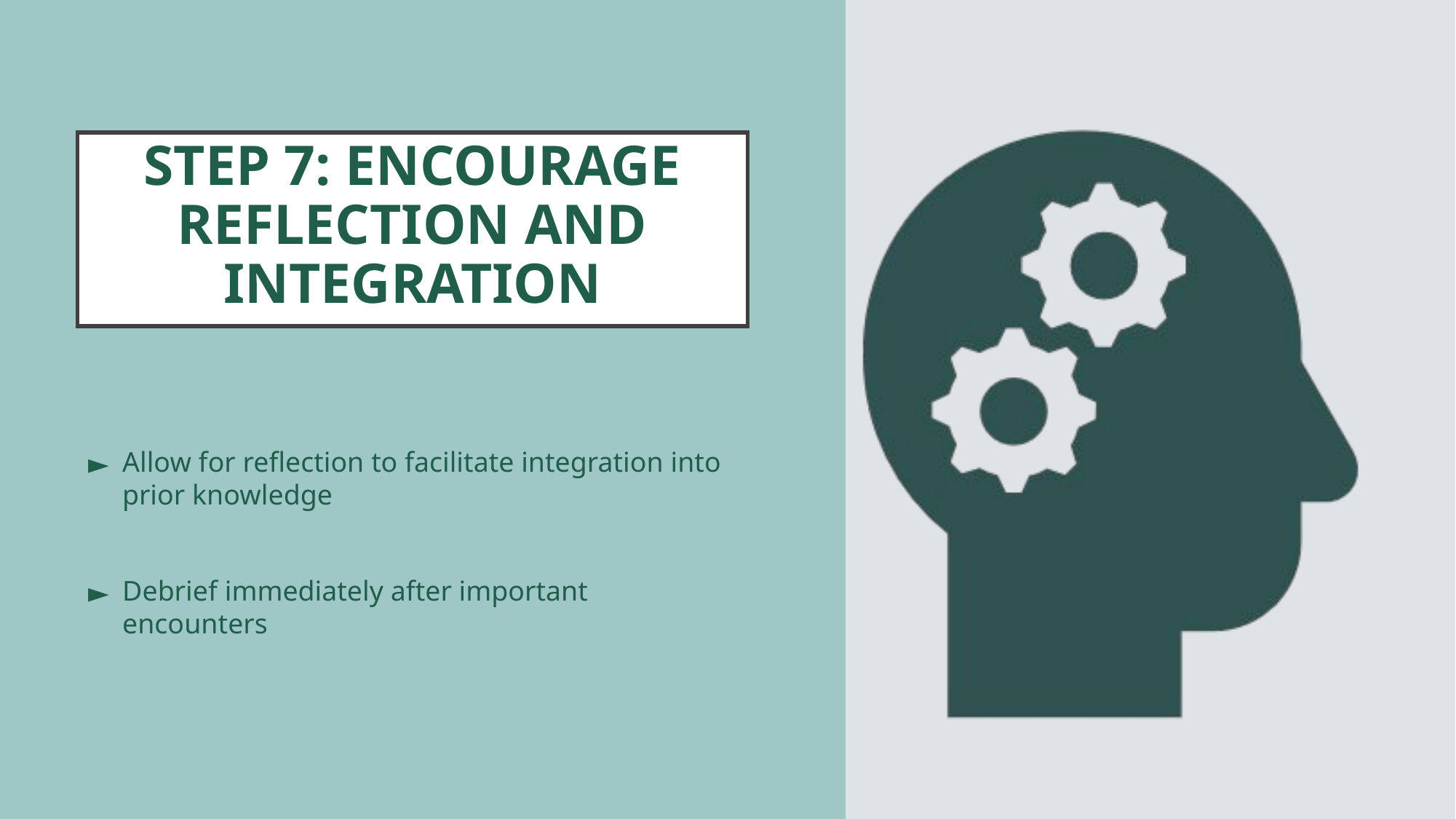

# STEP 7: ENCOURAGE REFLECTION AND INTEGRATION
Allow for reflection to facilitate integration into prior knowledge
Debrief immediately after important encounters

## Slide 11
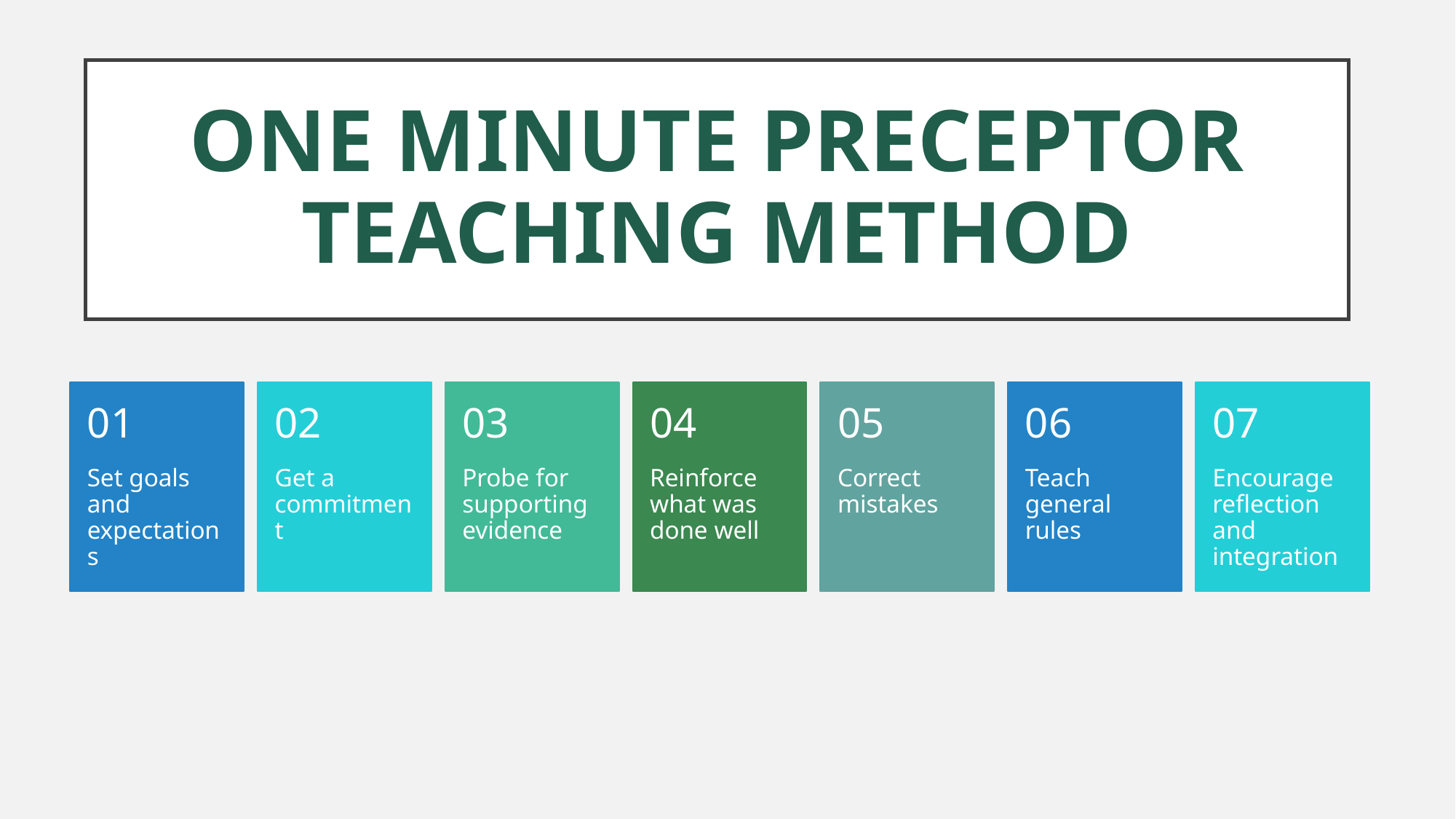

# ONE MINUTE PRECEPTOR TEACHING METHOD
01
02
03
04
05
06
07
Set goals and expectations
Get a commitment
Probe for supporting evidence
Reinforce what was done well
Correct mistakes
Teach general rules
Encourage reflection and integration

## Slide 12
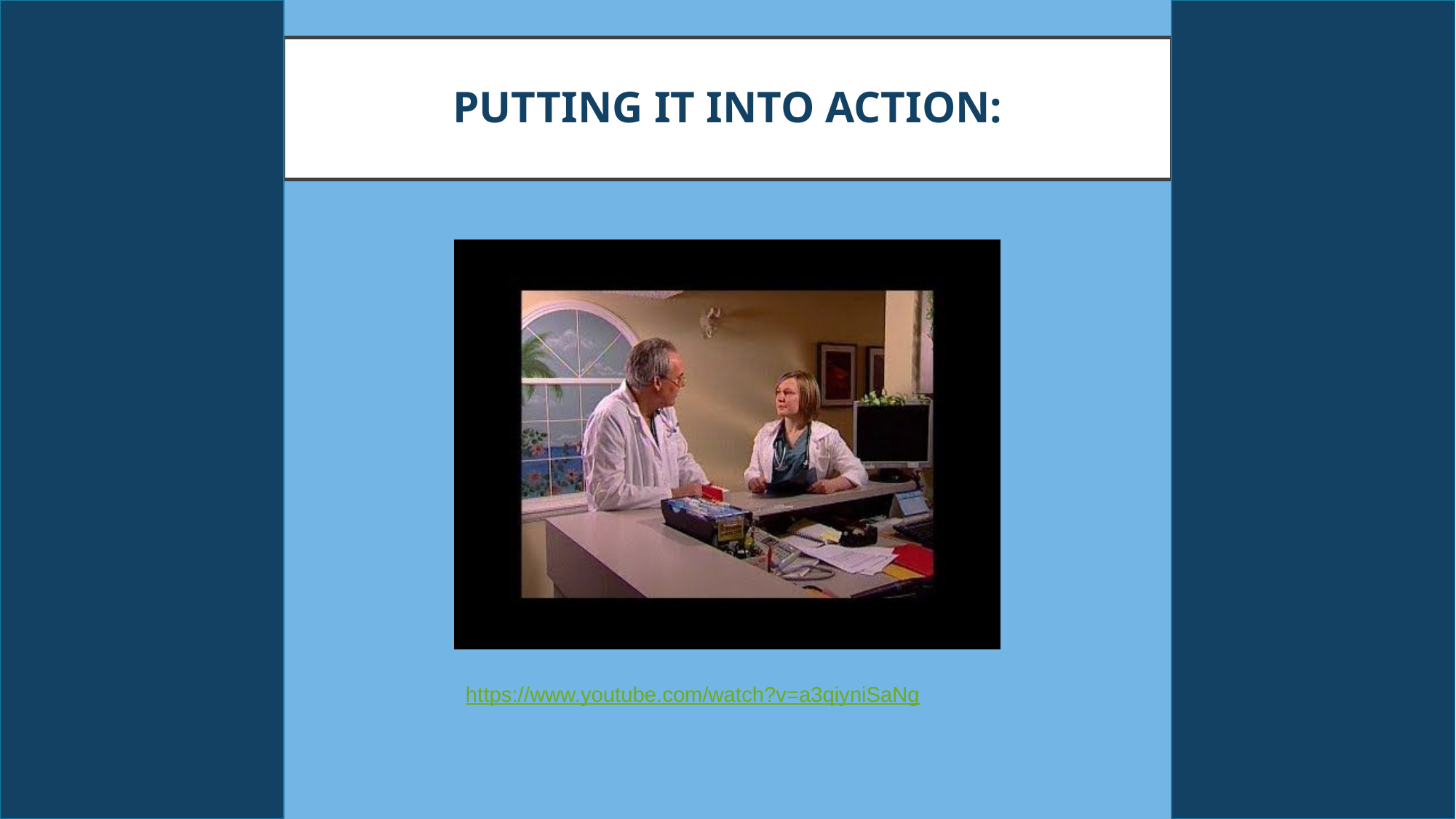

# PUTTING IT INTO ACTION:
https://www.youtube.com/watch?v=a3qiyniSaNg

## Slide 13
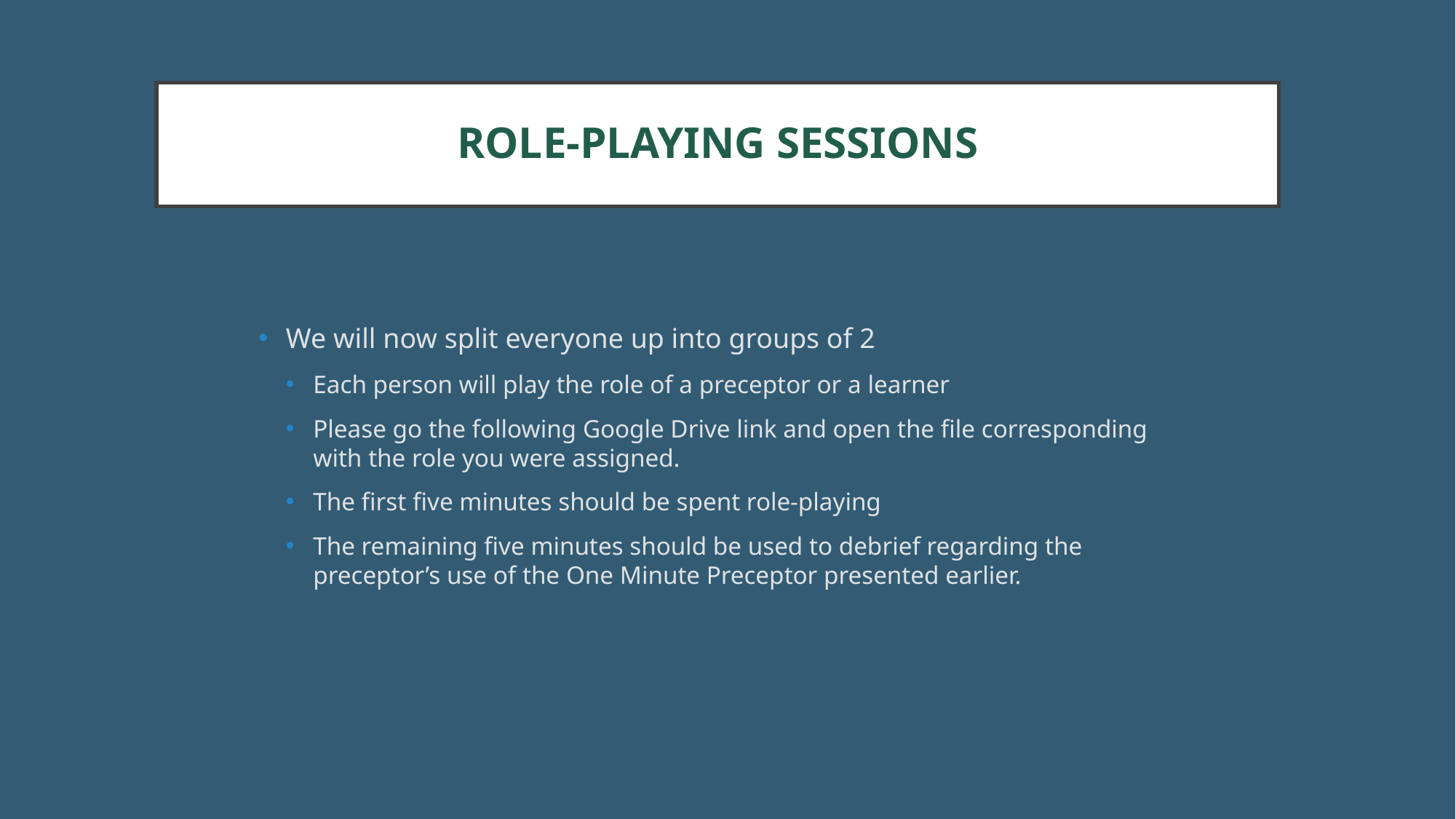

# ROLE-PLAYING SESSIONS
We will now split everyone up into groups of 2
Each person will play the role of a preceptor or a learner
Please go the following Google Drive link and open the file corresponding with the role you were assigned.
The first five minutes should be spent role-playing
The remaining five minutes should be used to debrief regarding the preceptor’s use of the One Minute Preceptor presented earlier.

## Slide 14
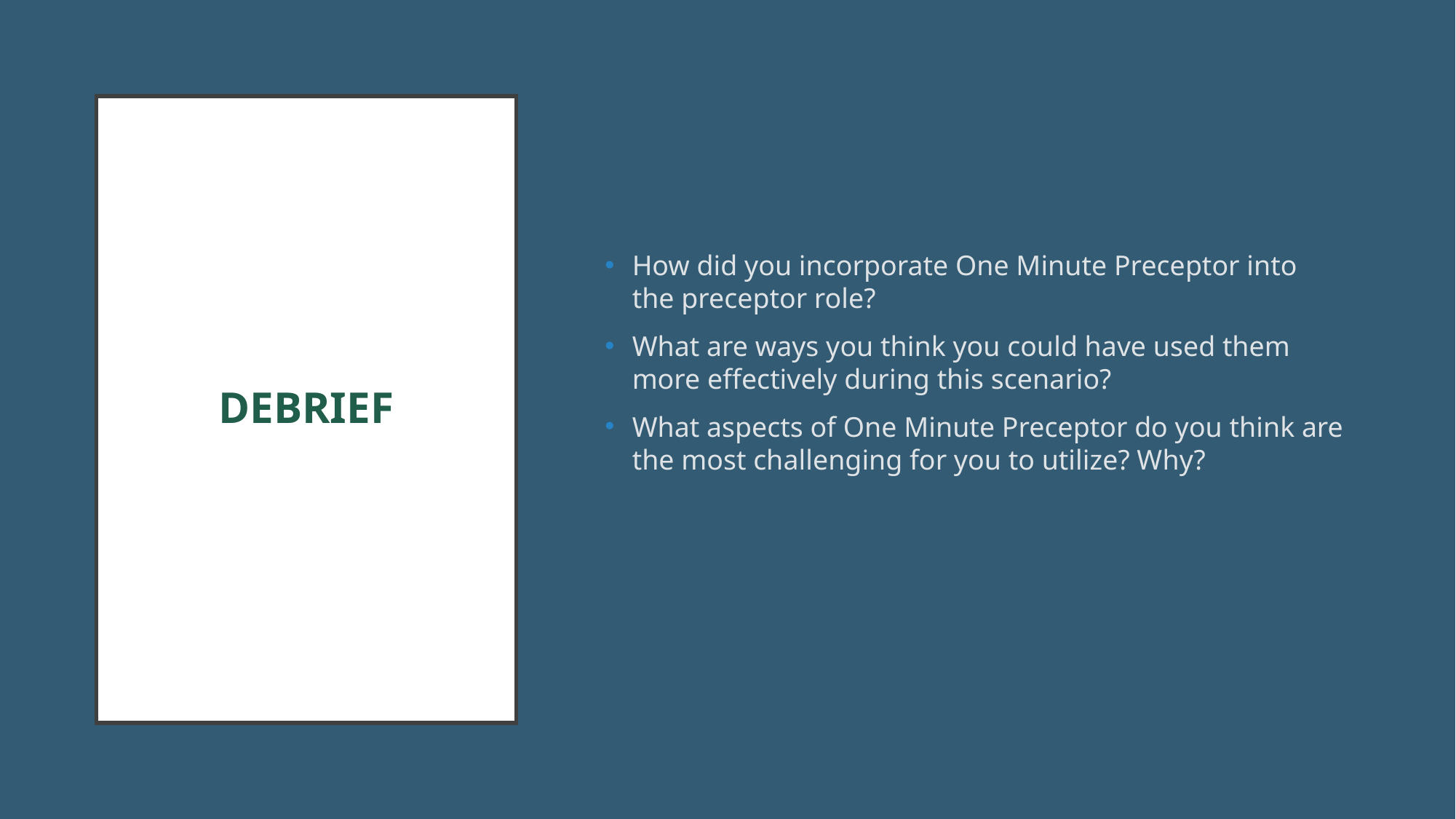

How did you incorporate One Minute Preceptor into the preceptor role?
What are ways you think you could have used them more effectively during this scenario?
What aspects of One Minute Preceptor do you think are the most challenging for you to utilize? Why?
# DEBRIEF

## Slide 15
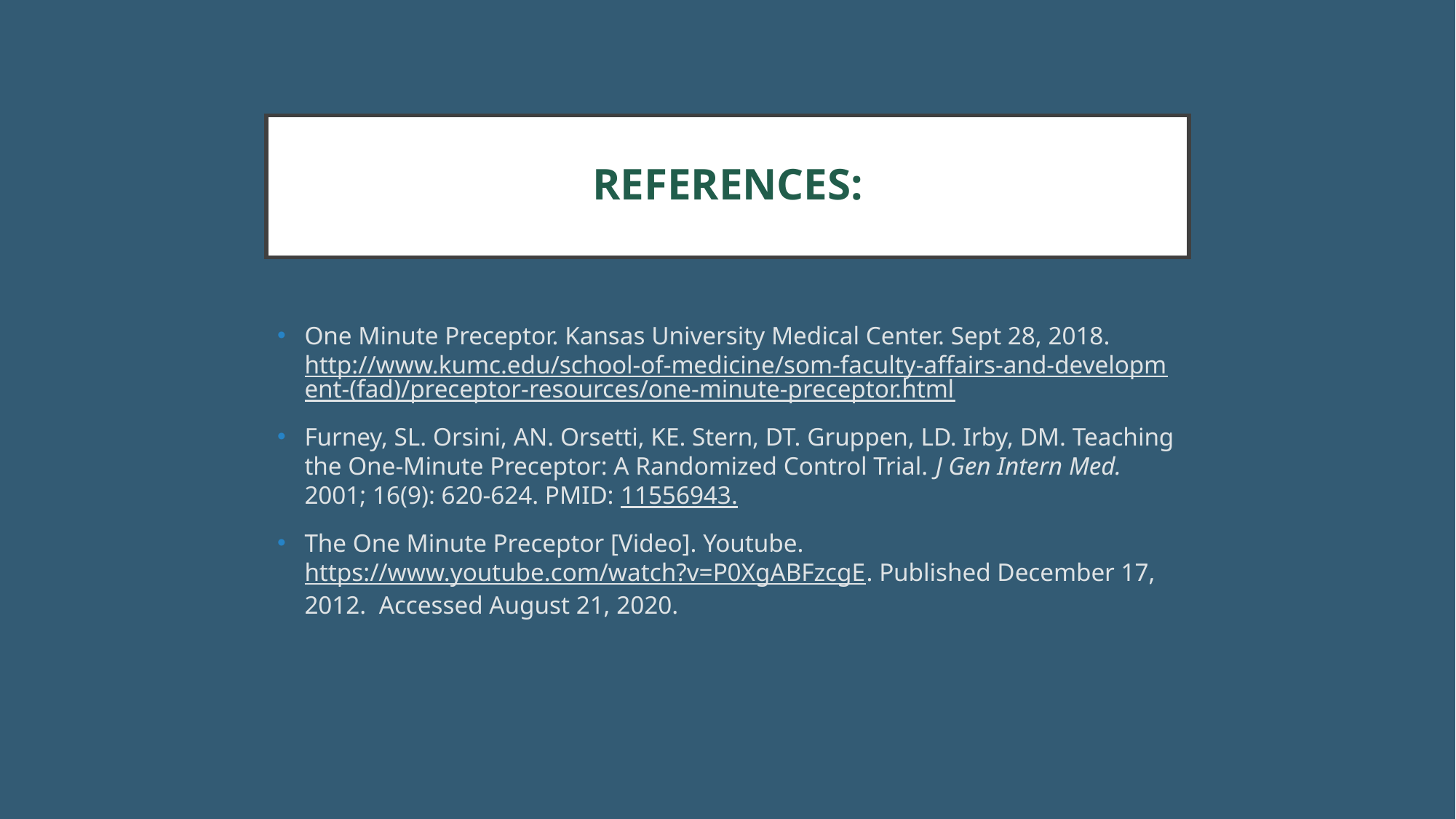

# REFERENCES:
One Minute Preceptor. Kansas University Medical Center. Sept 28, 2018. http://www.kumc.edu/school-of-medicine/som-faculty-affairs-and-development-(fad)/preceptor-resources/one-minute-preceptor.html
Furney, SL. Orsini, AN. Orsetti, KE. Stern, DT. Gruppen, LD. Irby, DM. Teaching the One-Minute Preceptor: A Randomized Control Trial. J Gen Intern Med. 2001; 16(9): 620-624. PMID: 11556943.
The One Minute Preceptor [Video]. Youtube. https://www.youtube.com/watch?v=P0XgABFzcgE. Published December 17, 2012. Accessed August 21, 2020.
