# Supplementary material for: A Novel Virtual Emergency Medicine Residents-as-Teachers (RAT) Curriculum
Source: J Educ Teach Emerg Med. 2021 Jul 15;6(3):C9–C63. doi: 10.21980/J86S71 (PMC10332683; doi:10.21980/J86S71)
Supplement: Supplementary file 3 — Please see associated PowerPoint file [file jetem-6-3-c8-Appendix-3c.pptx]

## Slide 1
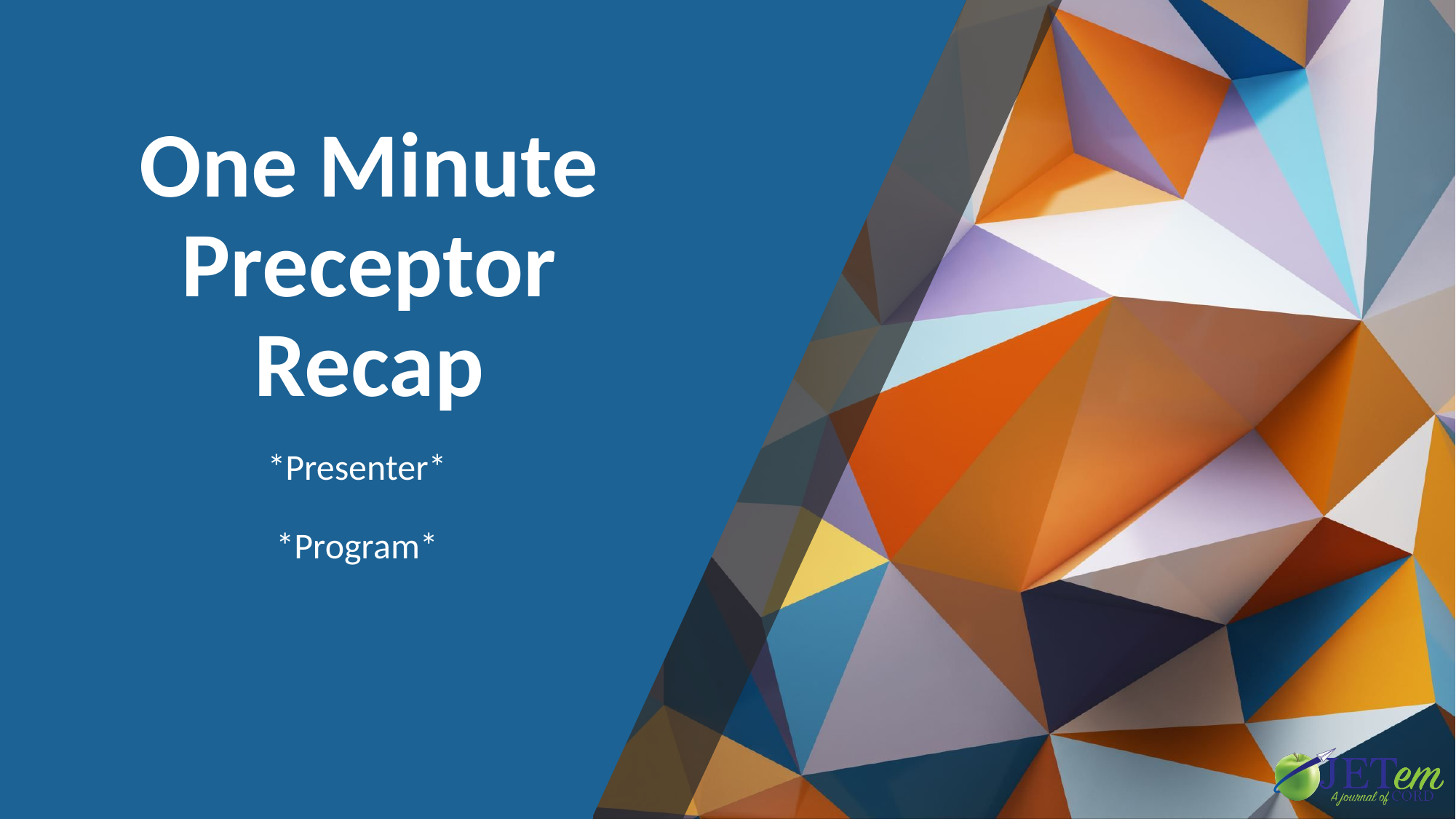

# One Minute Preceptor Recap
*Presenter*
*Program*

## Slide 2
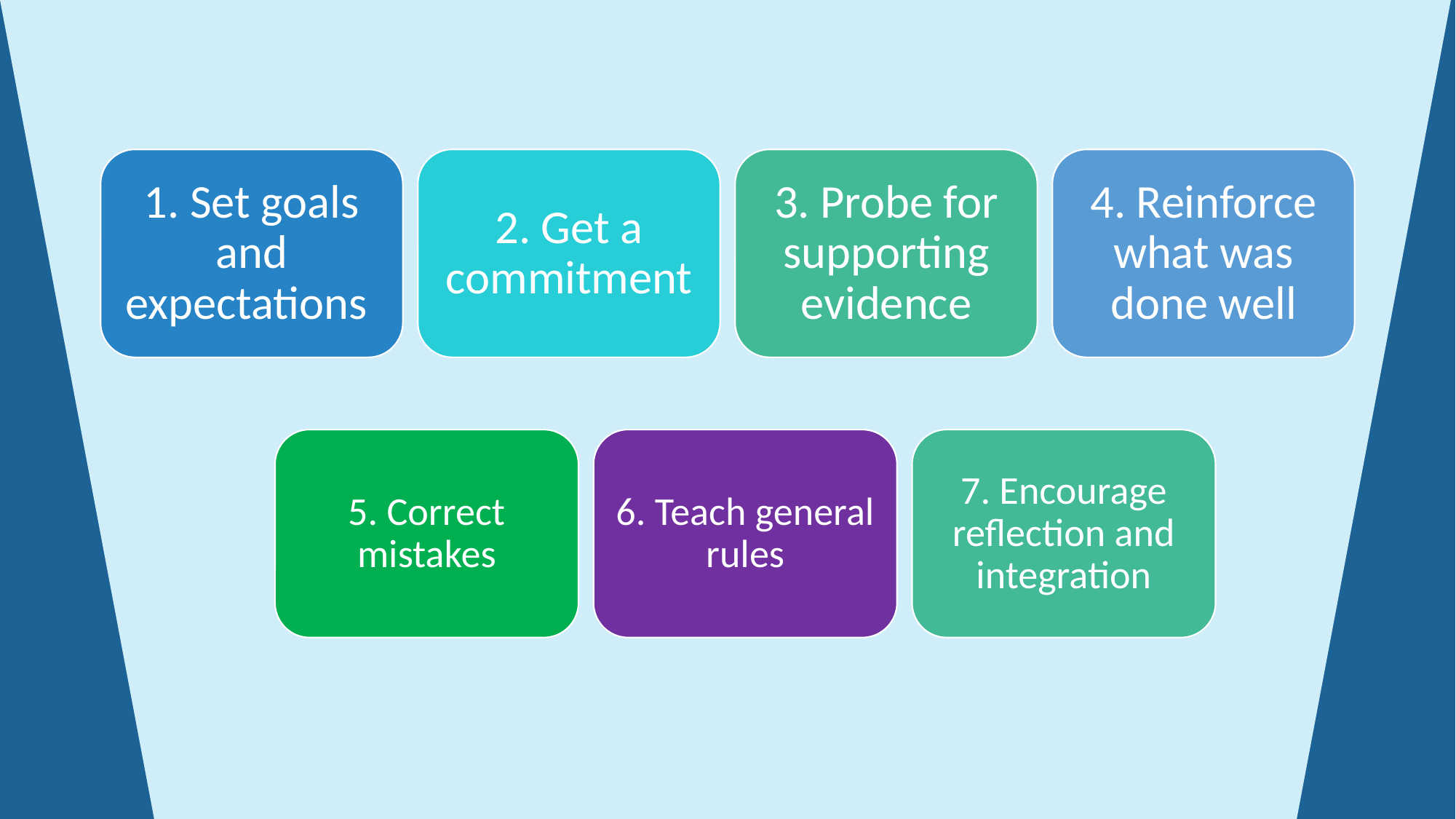

1. Set goals and expectations
2. Get a commitment
3. Probe for supporting evidence
4. Reinforce what was done well
5. Correct mistakes
6. Teach general rules
7. Encourage reflection and integration
