# Supplementary material for: A Novel Virtual Emergency Medicine Residents-as-Teachers (RAT) Curriculum
Source: J Educ Teach Emerg Med. 2021 Jul 15;6(3):C9–C63. doi: 10.21980/J86S71 (PMC10332683; doi:10.21980/J86S71)
Supplement: Supplementary file 4 — Please see associated PowerPoint file [file jetem-6-3-c8-Appendix3d.pptx]

## Slide 1
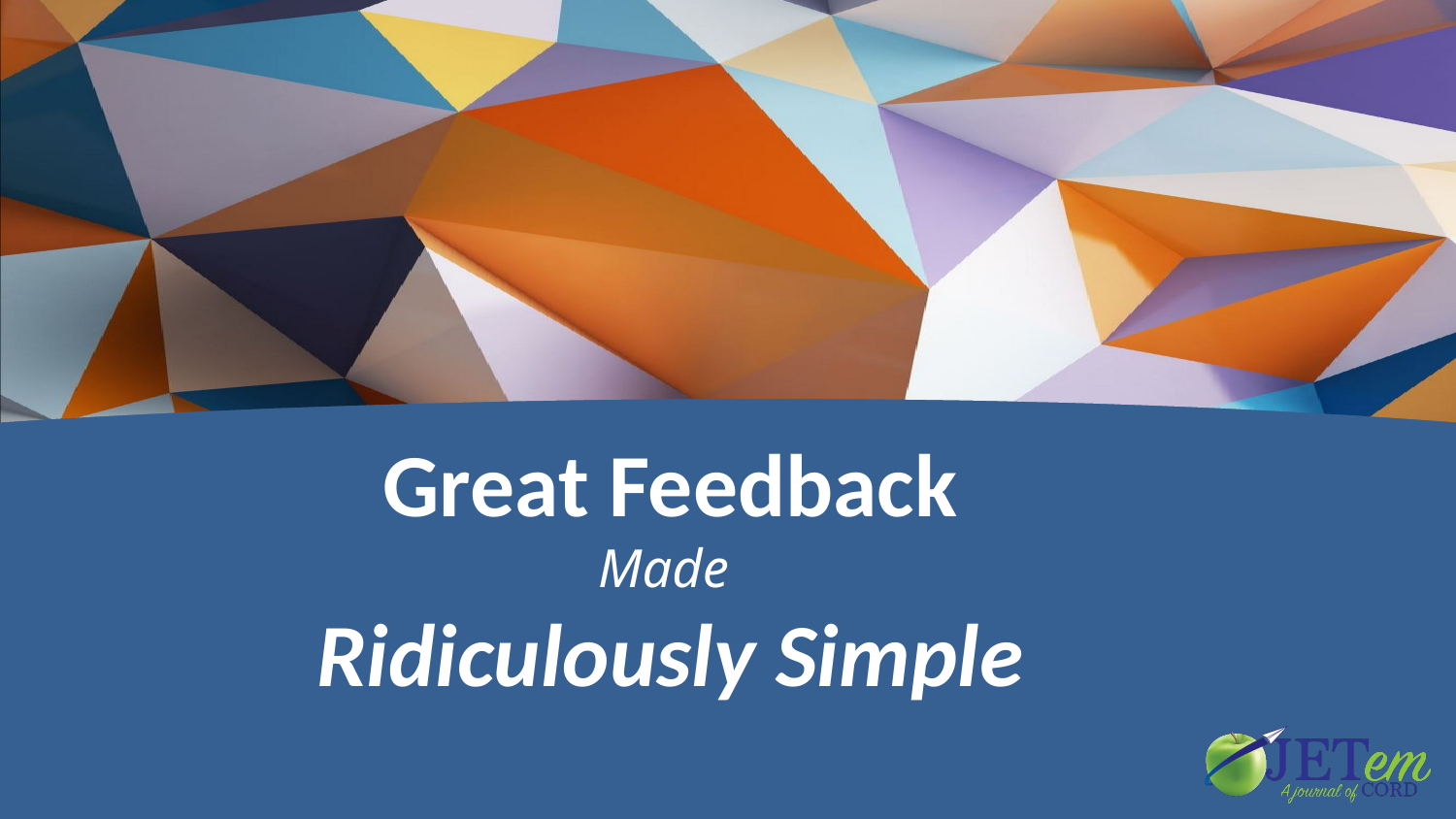

# Great Feedback Made Ridiculously Simple

## Slide 2
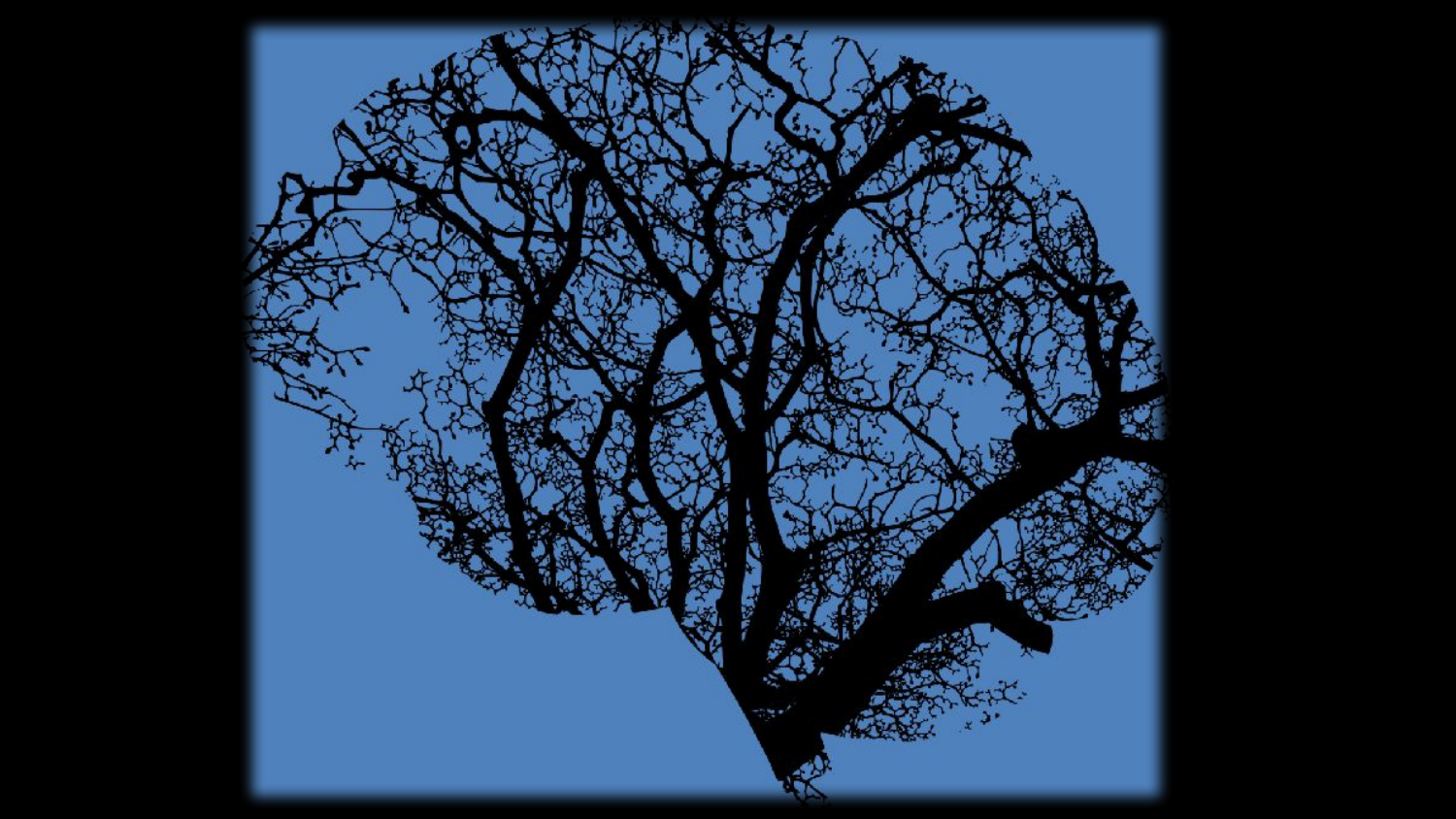

## Slide 3
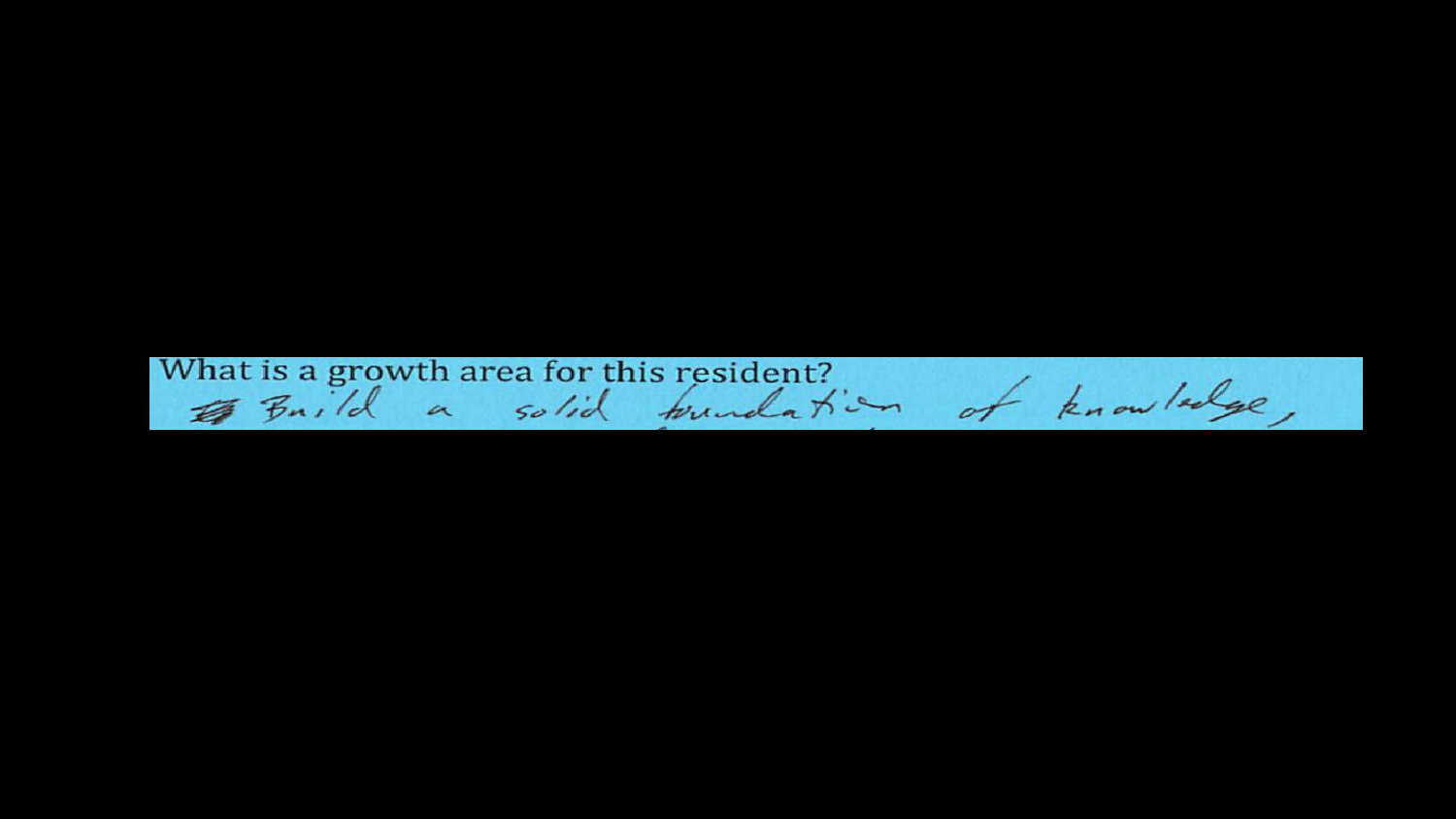

## Slide 4
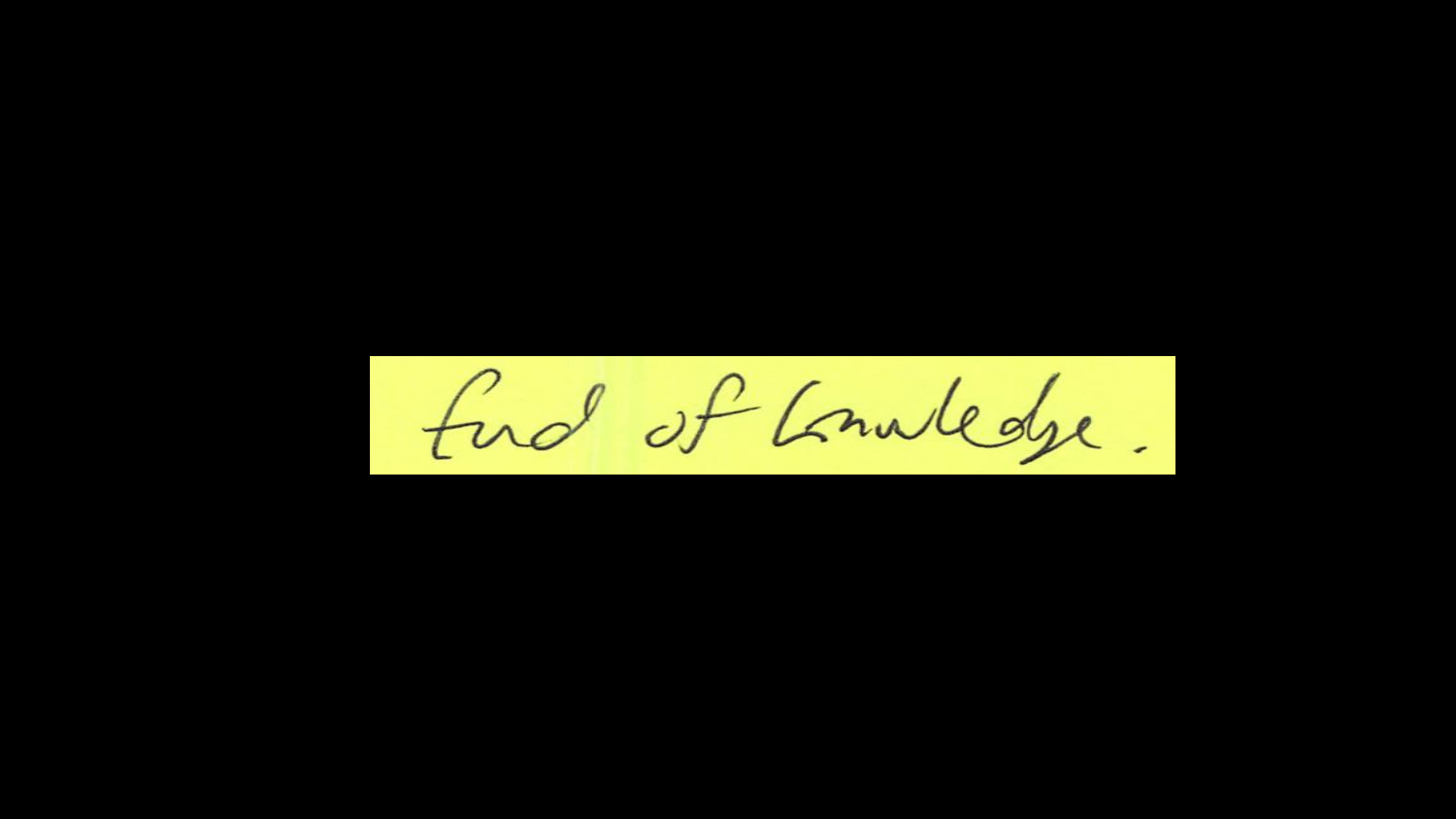

## Slide 5
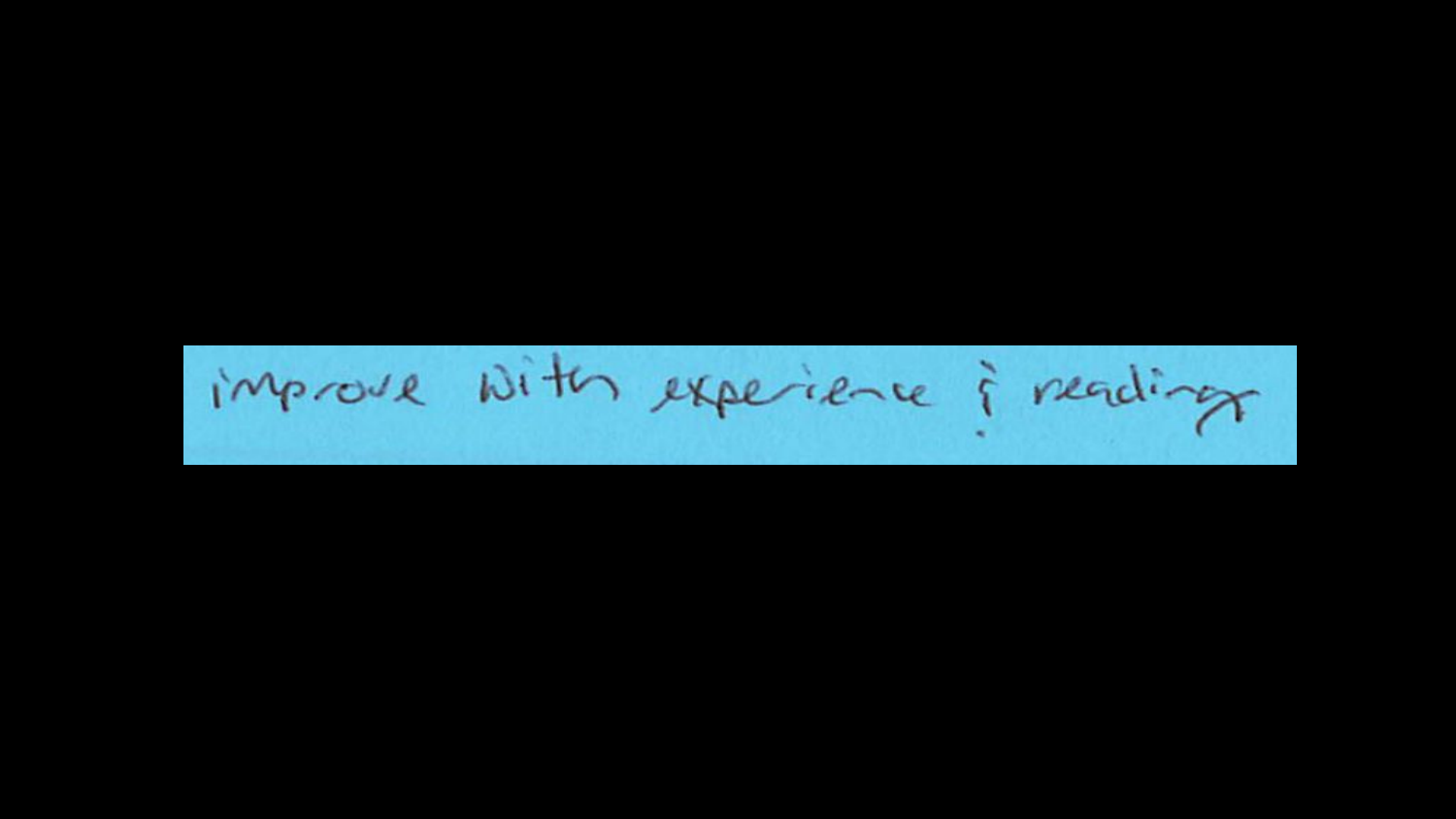

## Slide 6
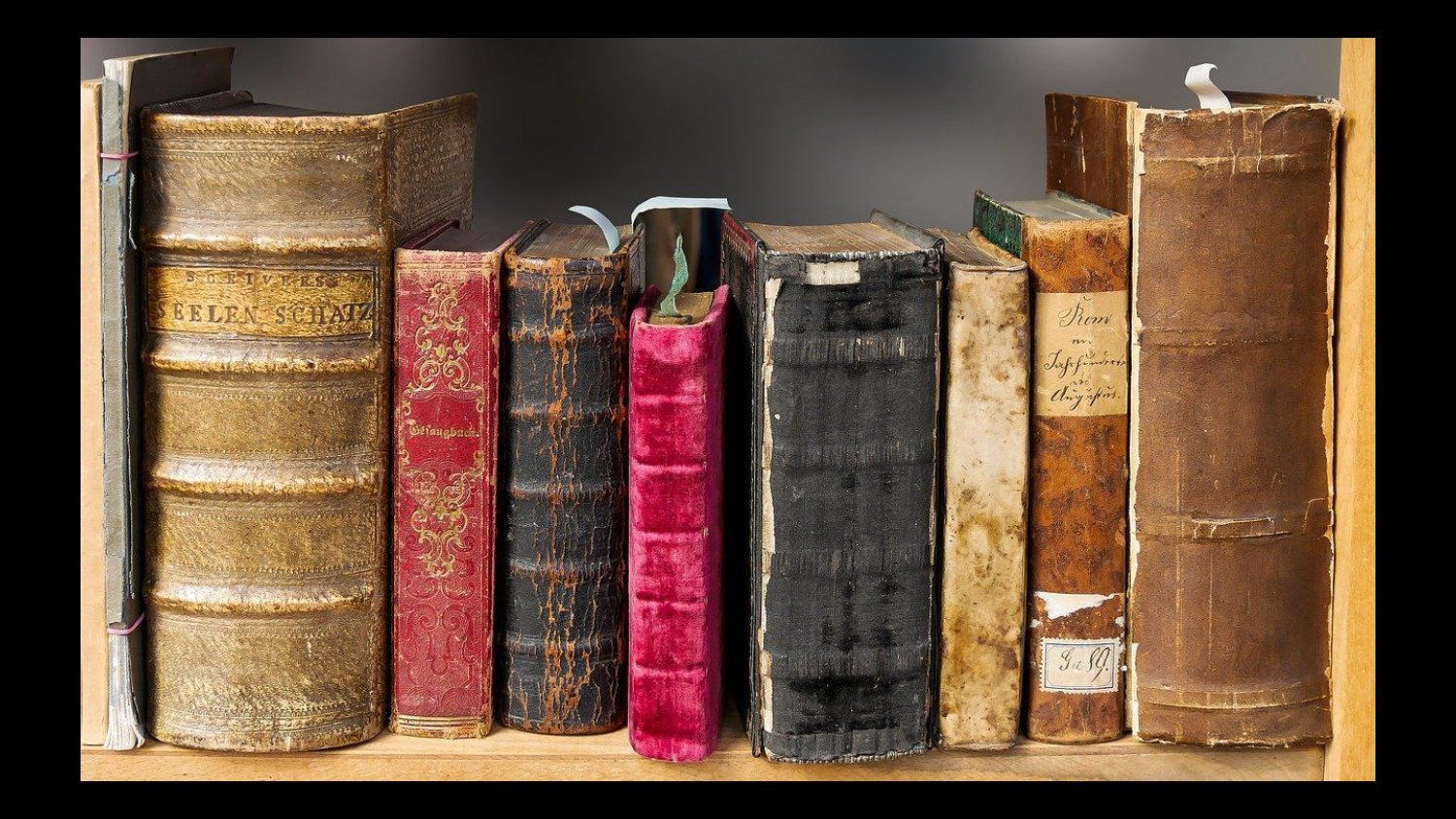

## Slide 7
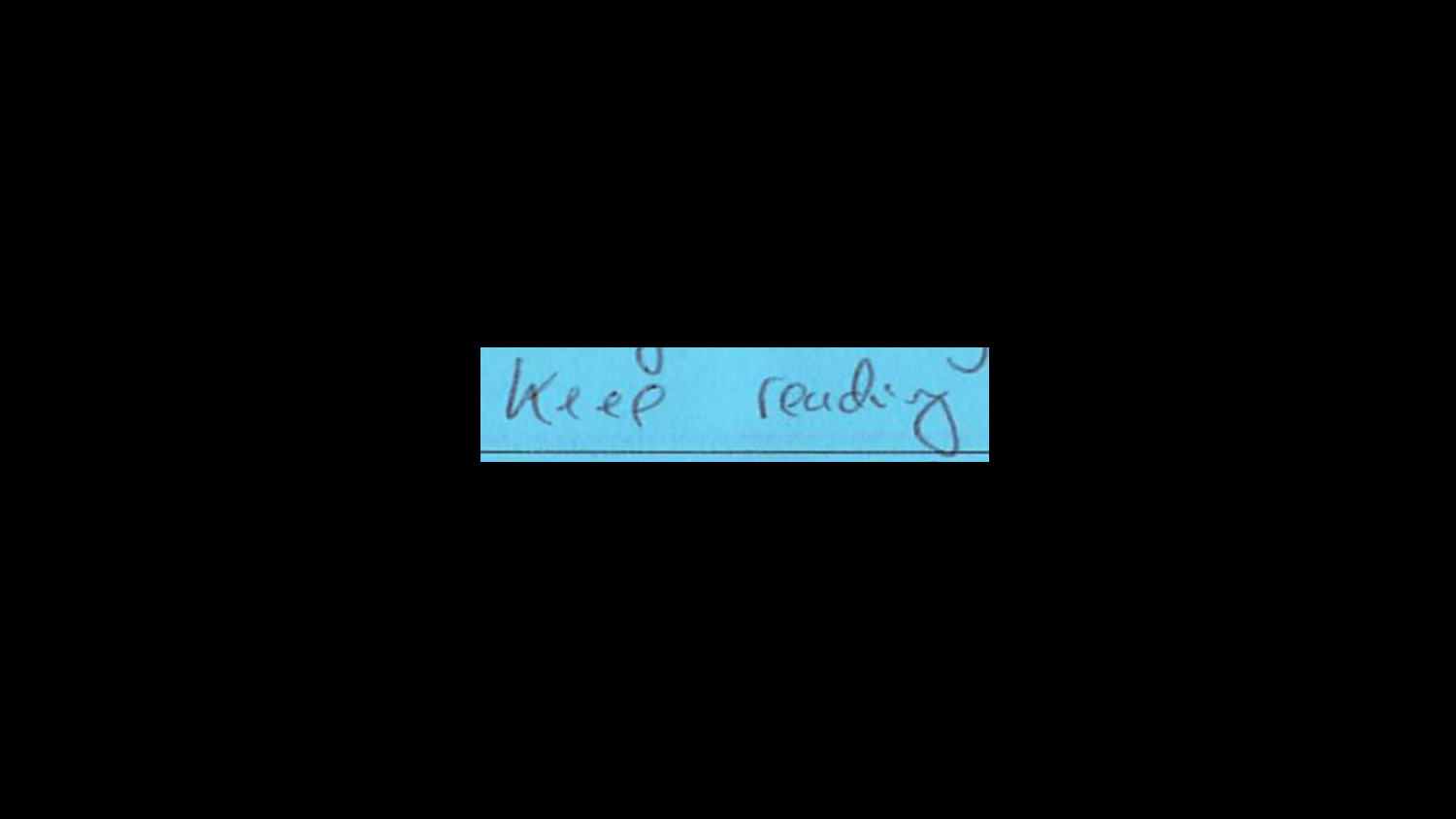

## Slide 8
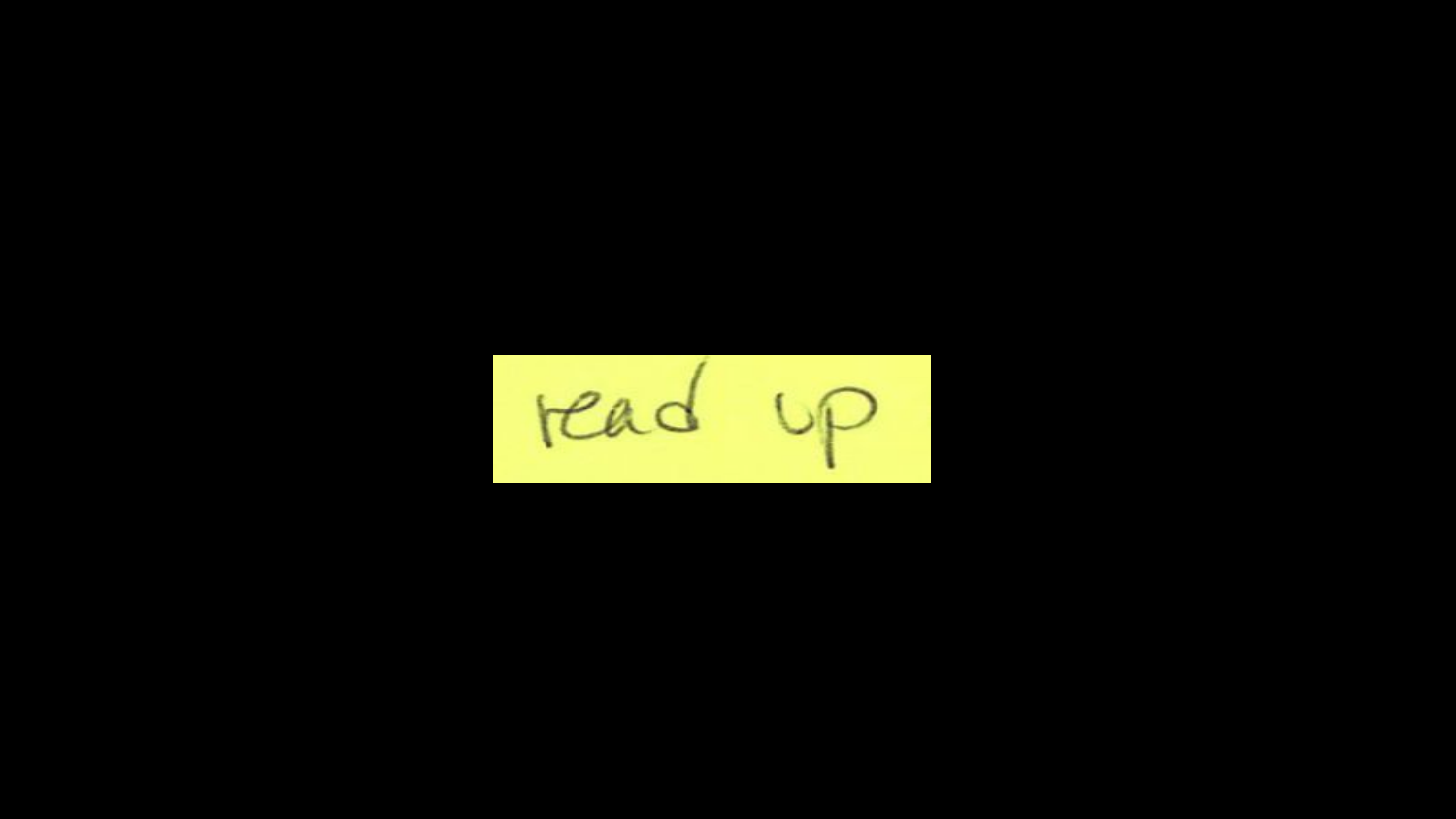

## Slide 9
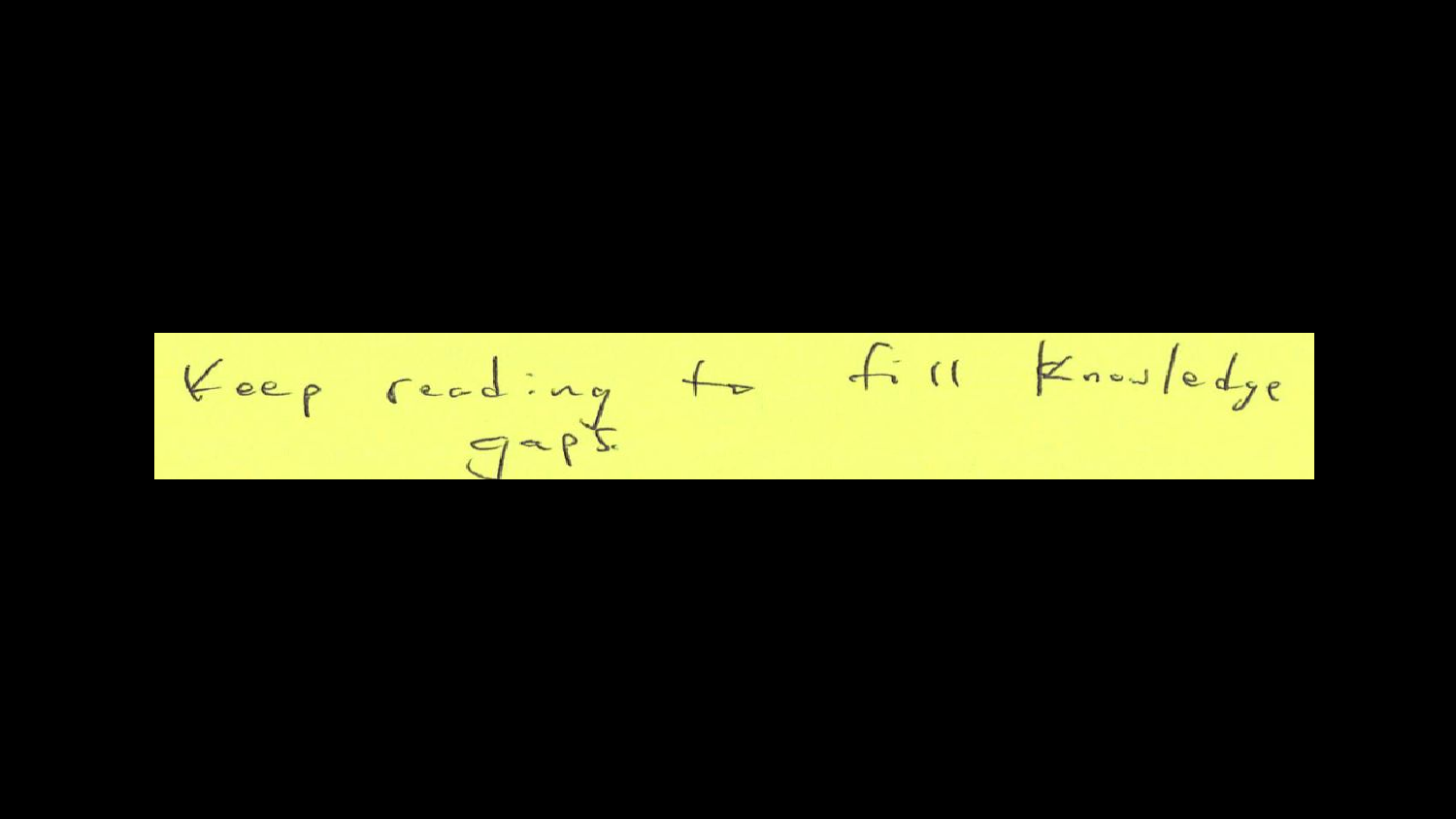

## Slide 10
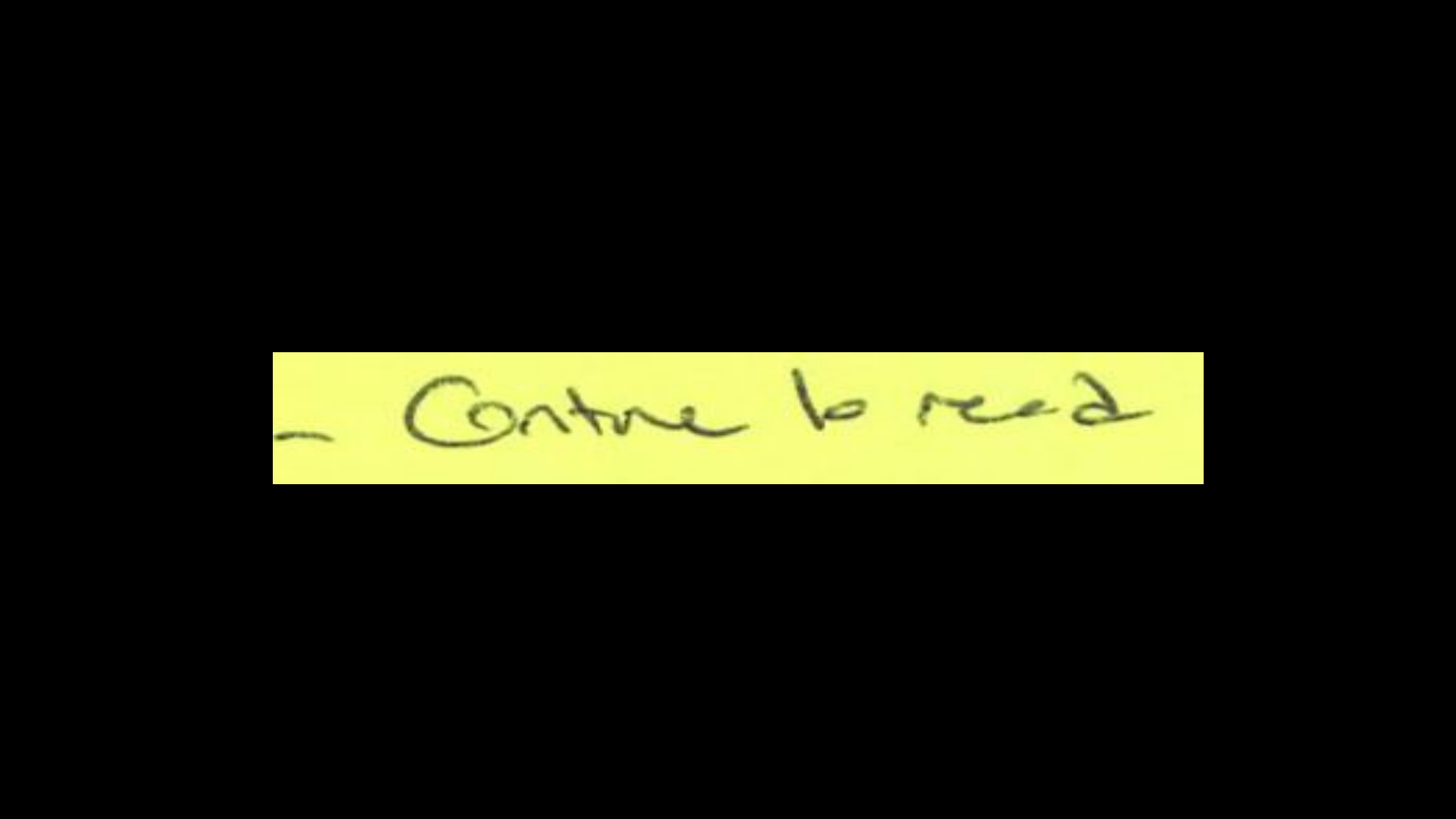

## Slide 11
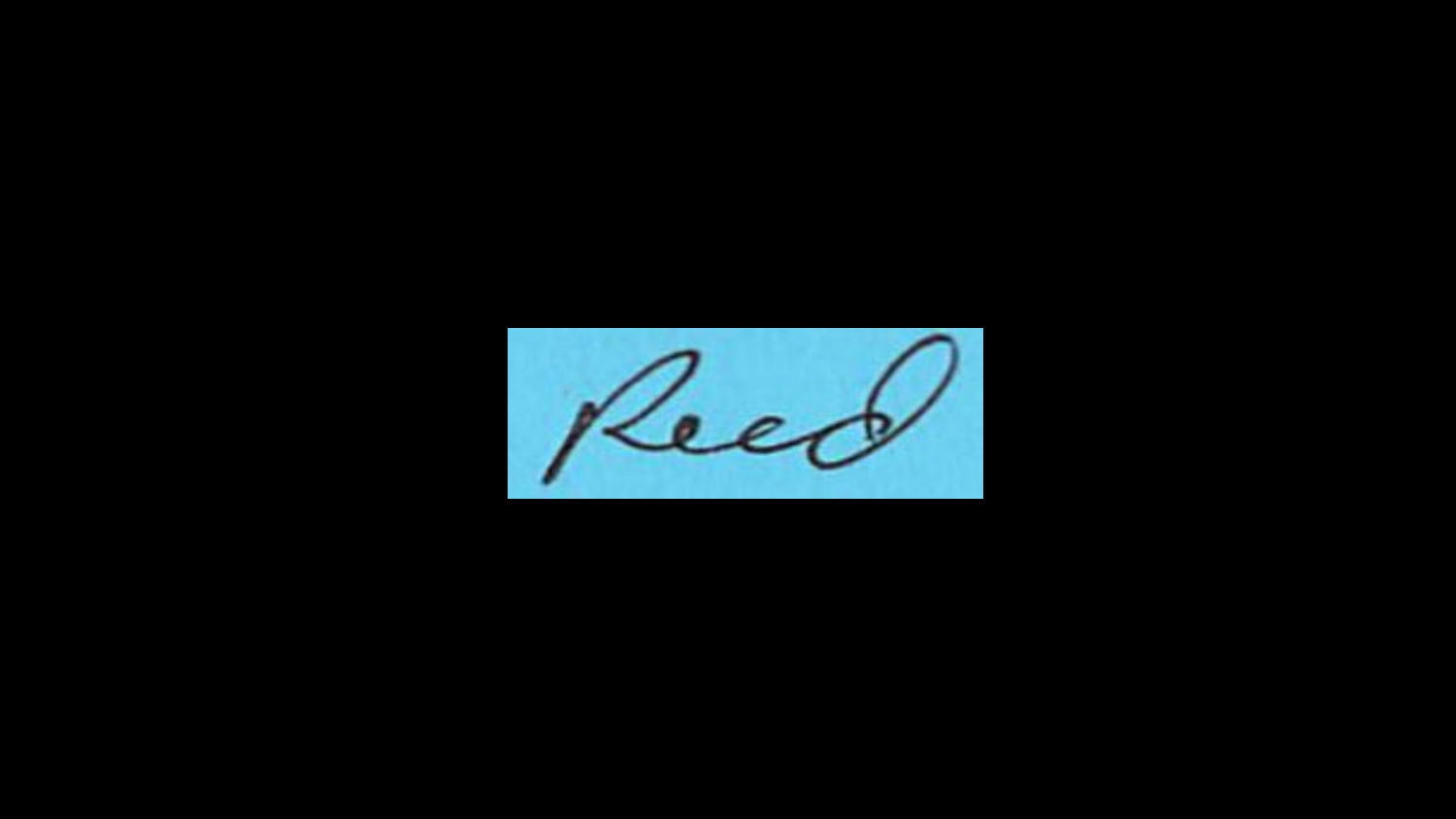

## Slide 12
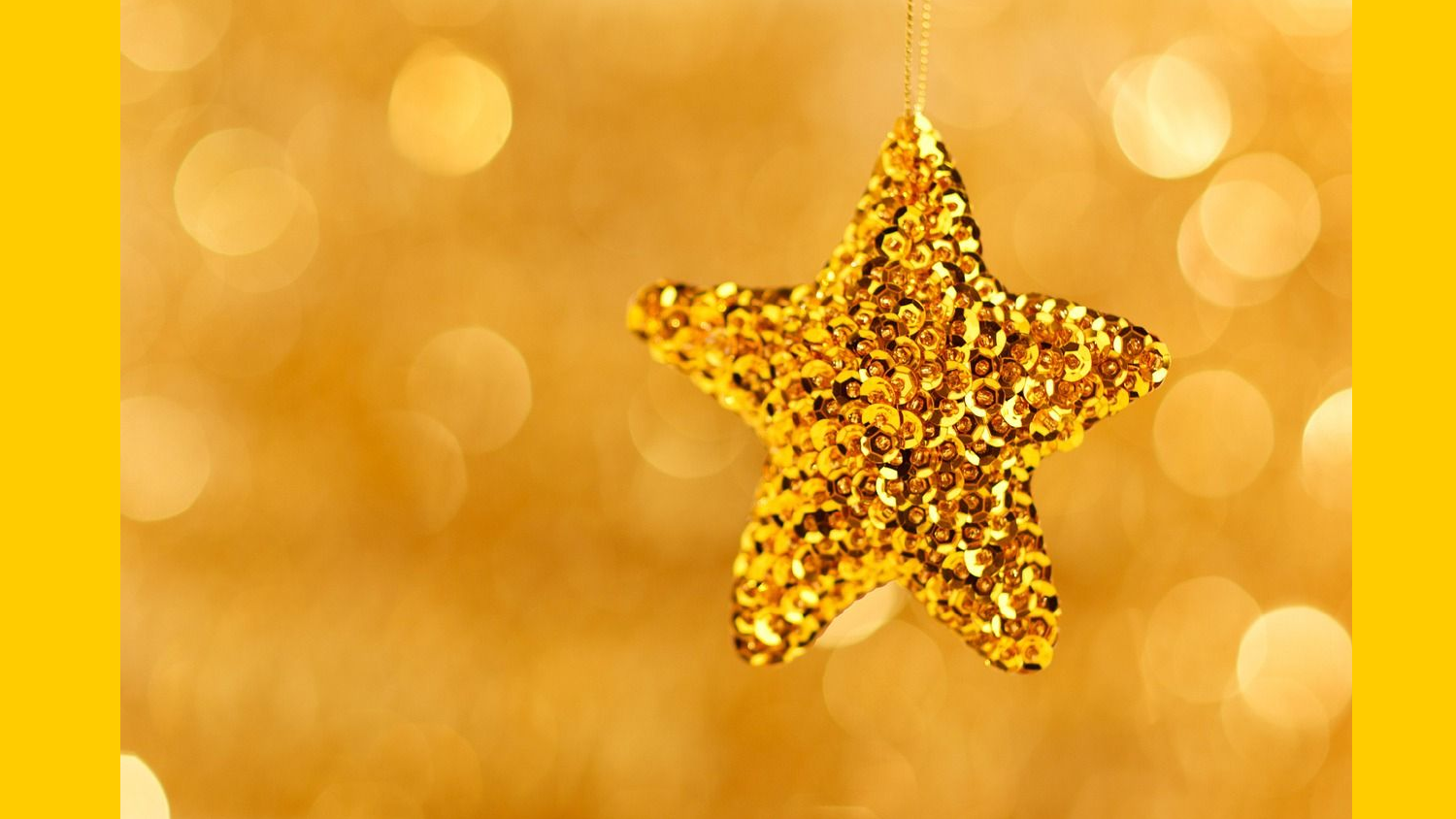

## Slide 13
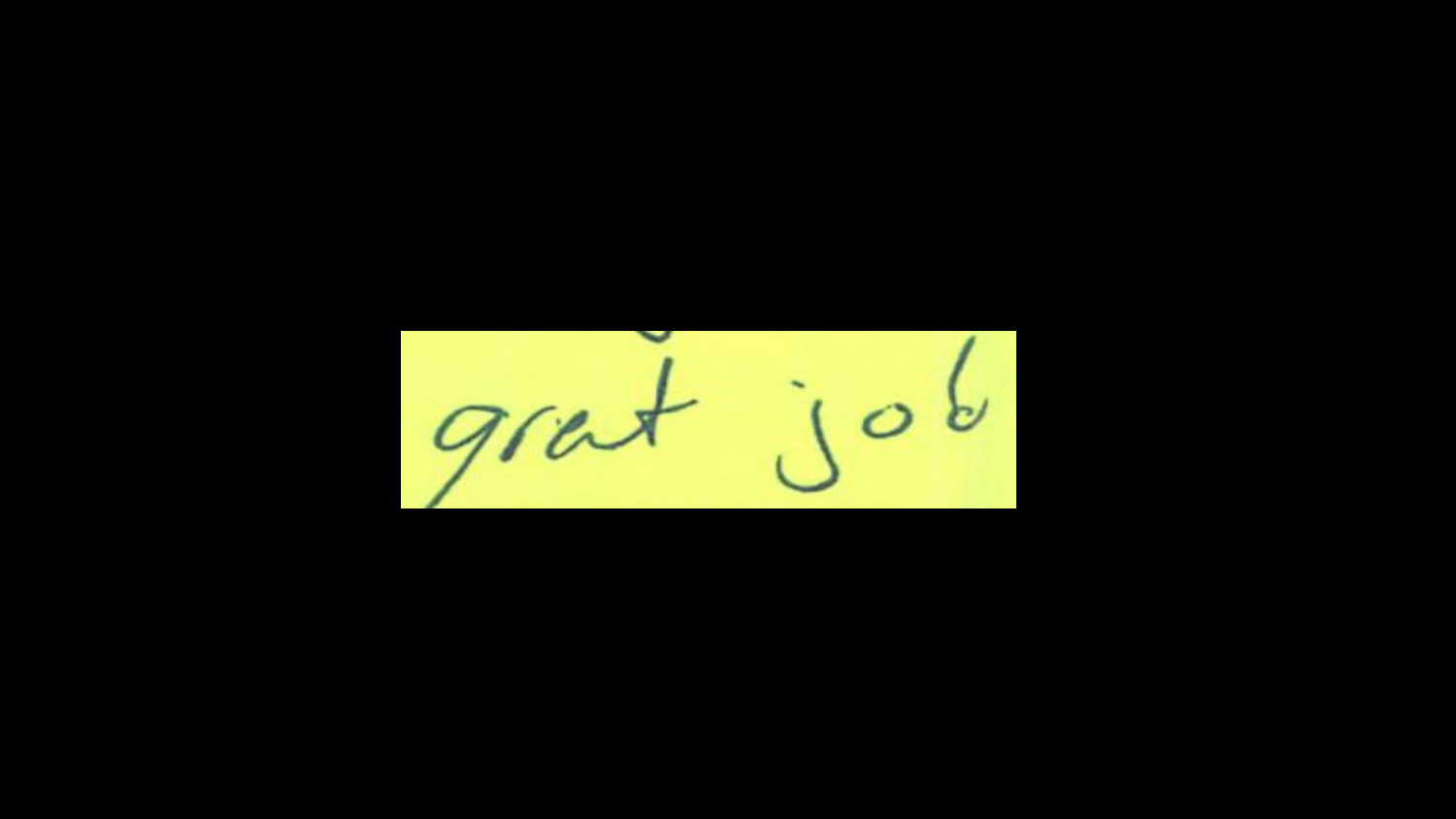

## Slide 14
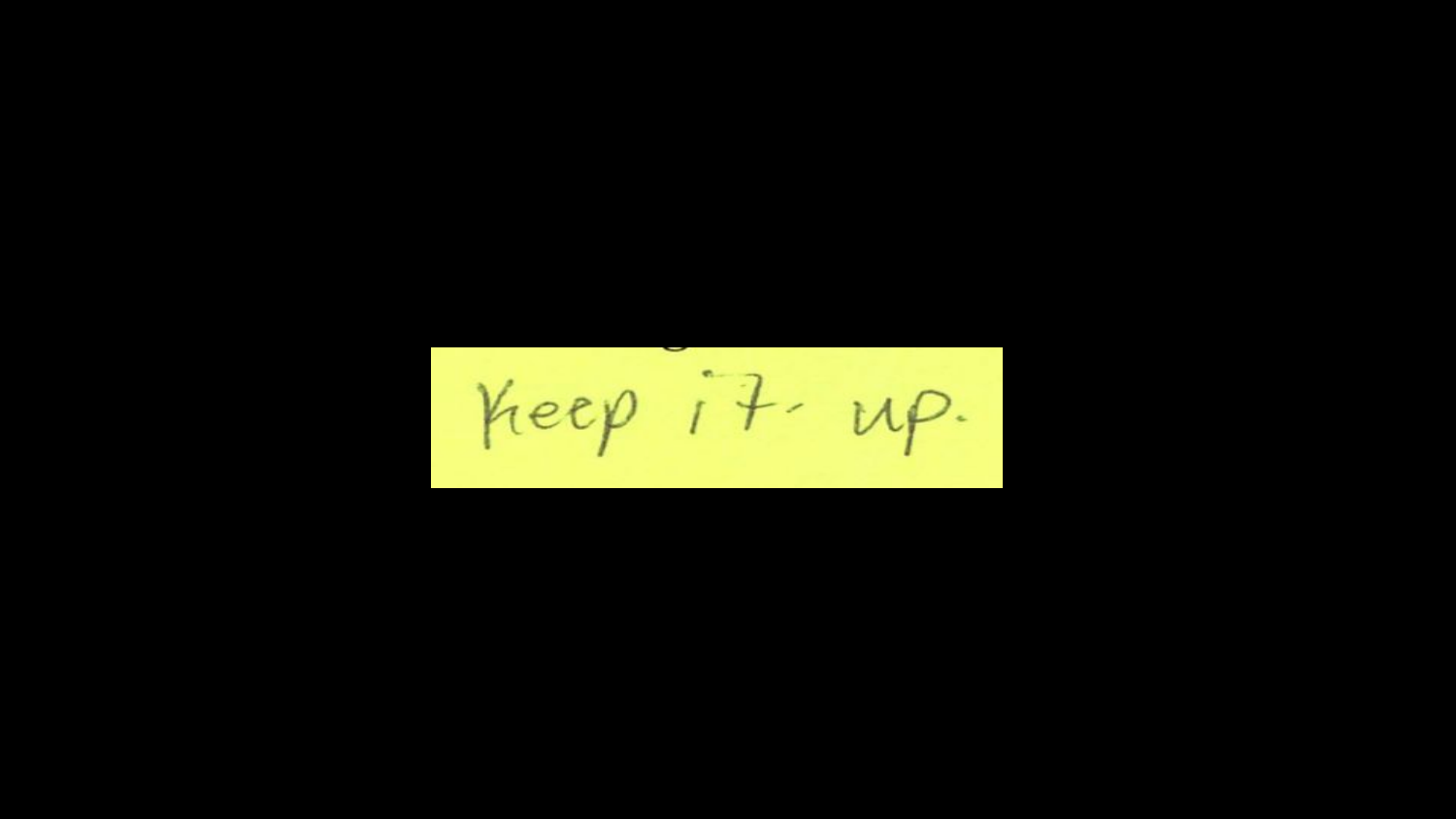

## Slide 15
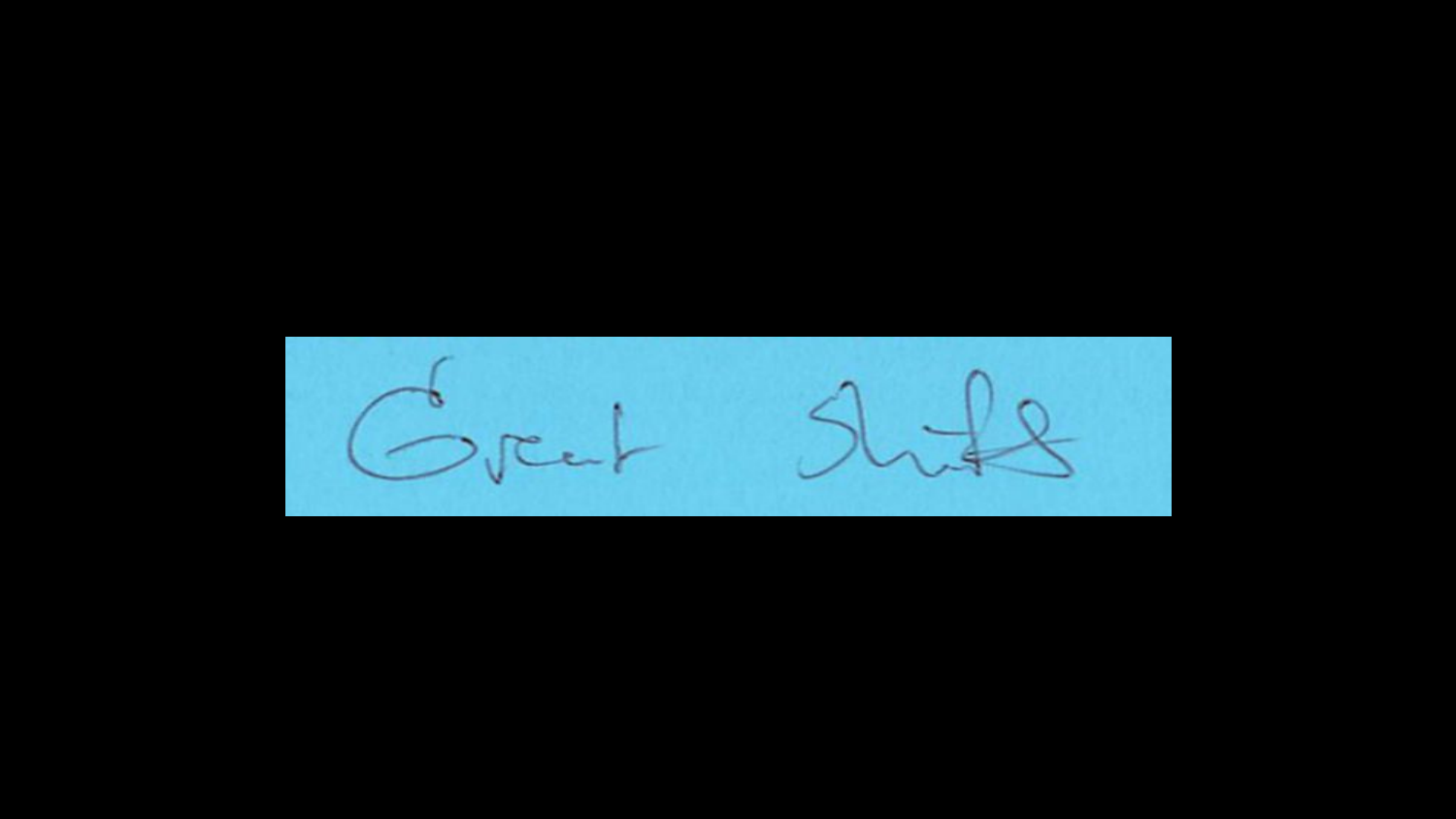

## Slide 16
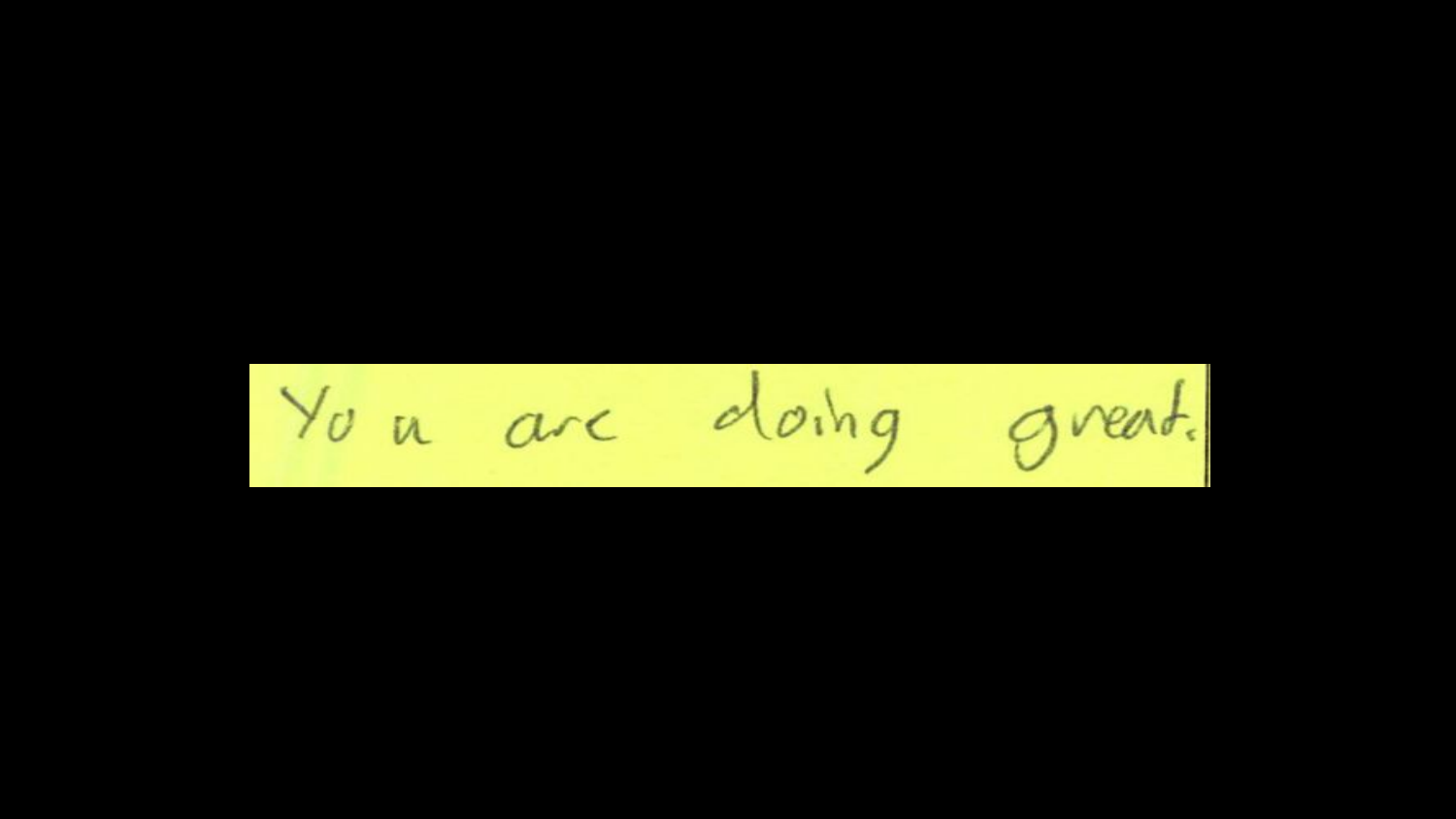

## Slide 17
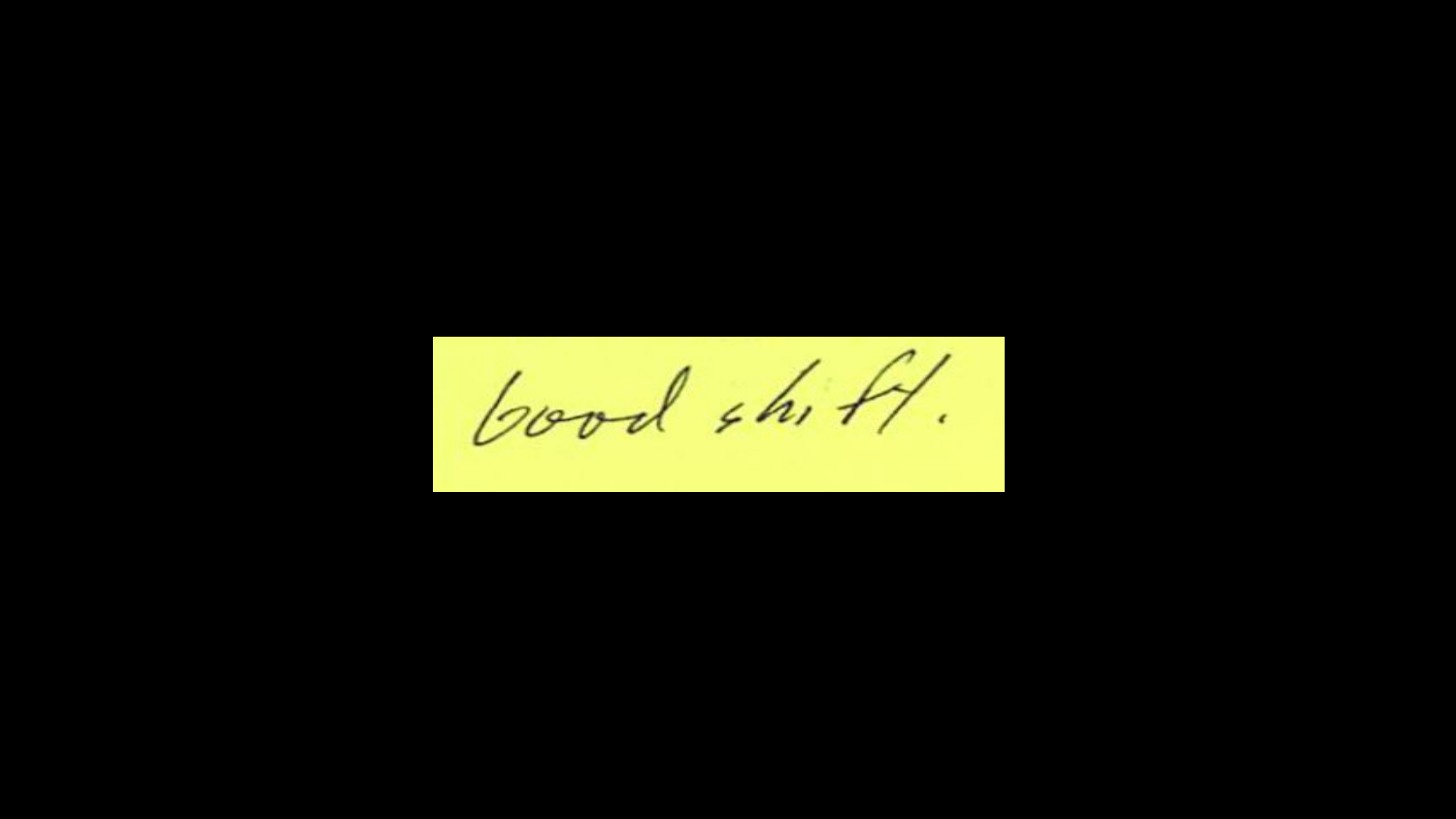

## Slide 18
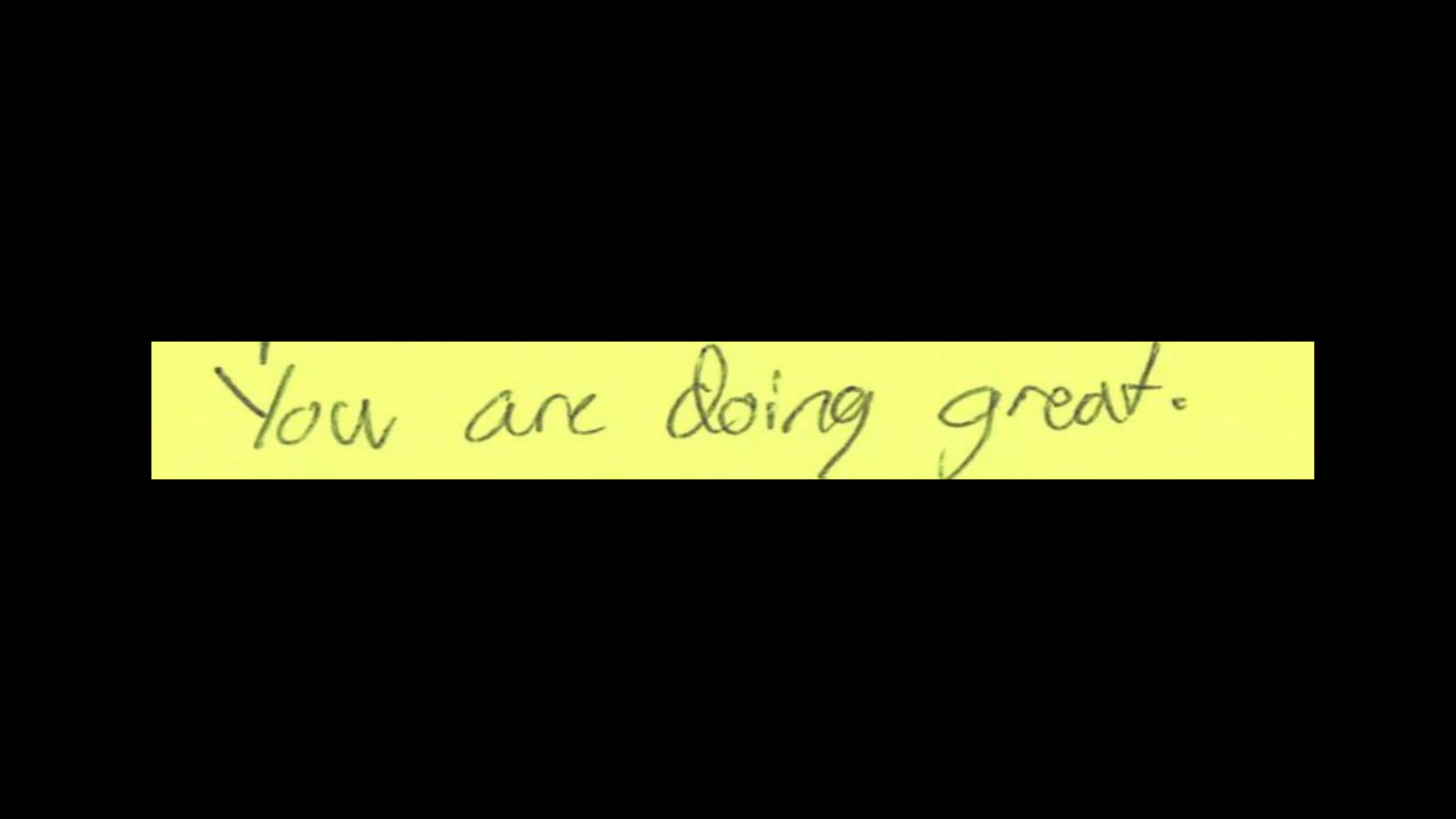

## Slide 19
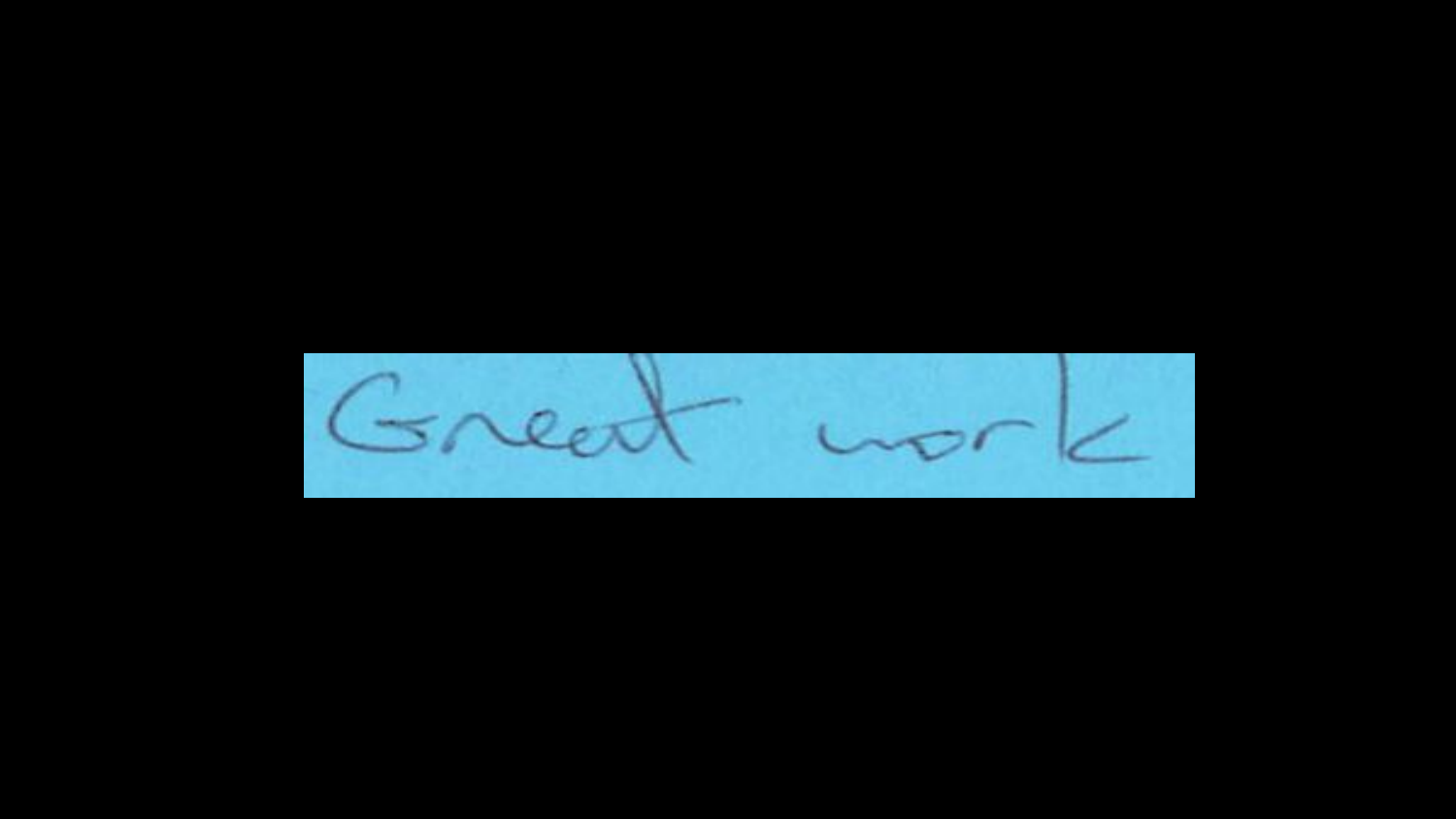

## Slide 20
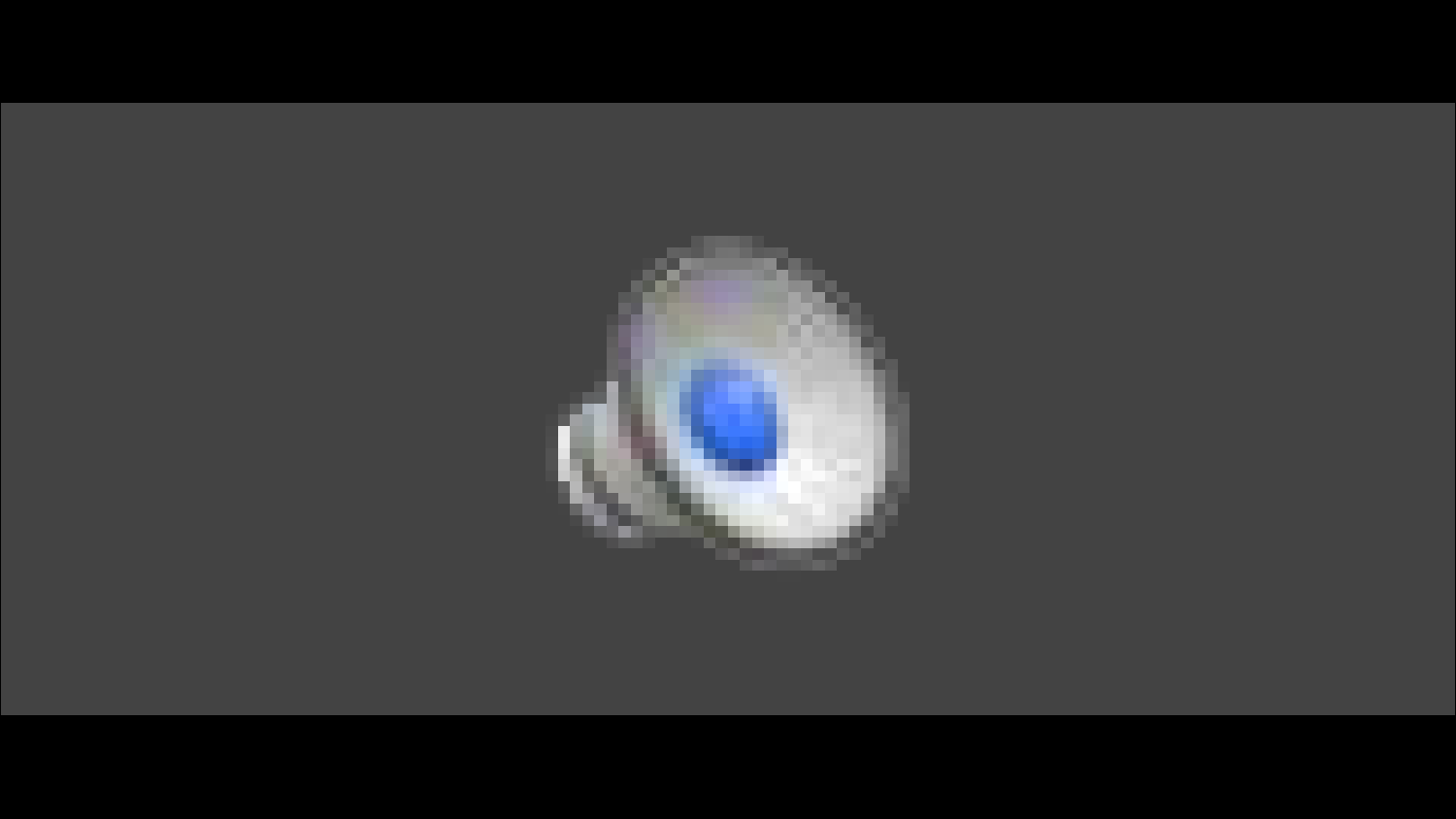

## Slide 21
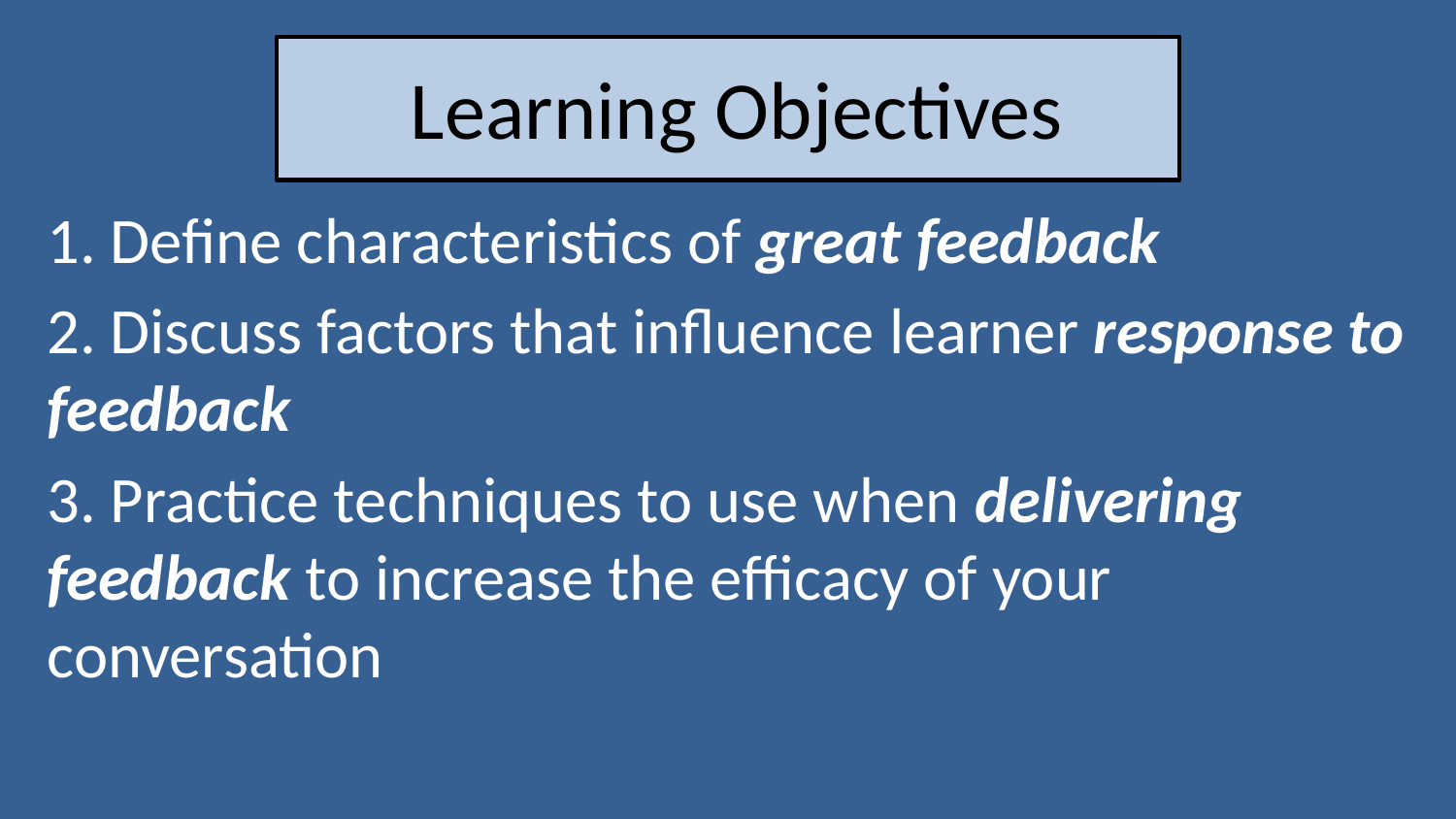

# Learning Objectives
1. Define characteristics of great feedback
2. Discuss factors that influence learner response to feedback
3. Practice techniques to use when delivering feedback to increase the efficacy of your conversation

## Slide 22
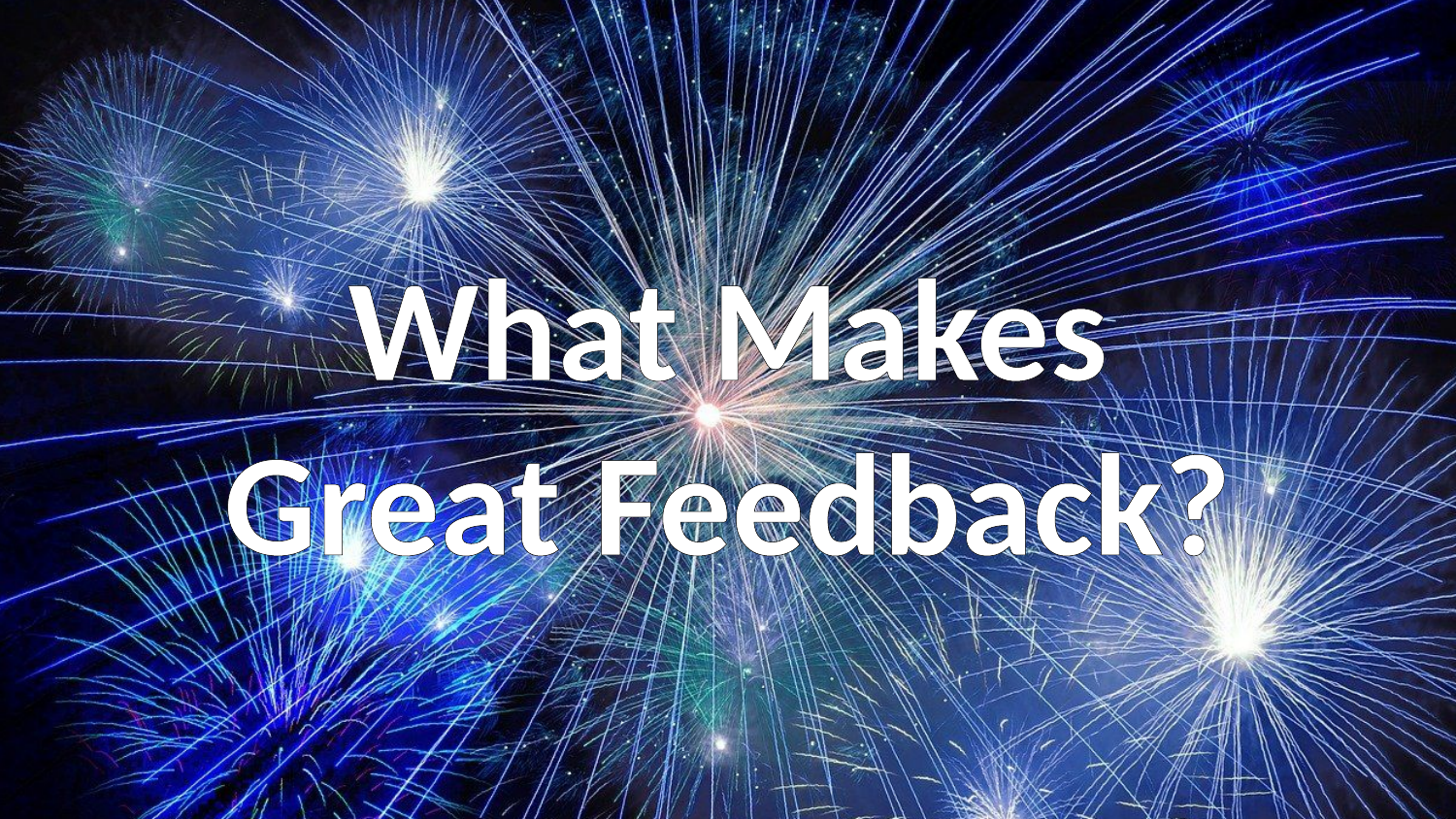

# What Makes Great Feedback?

## Slide 23
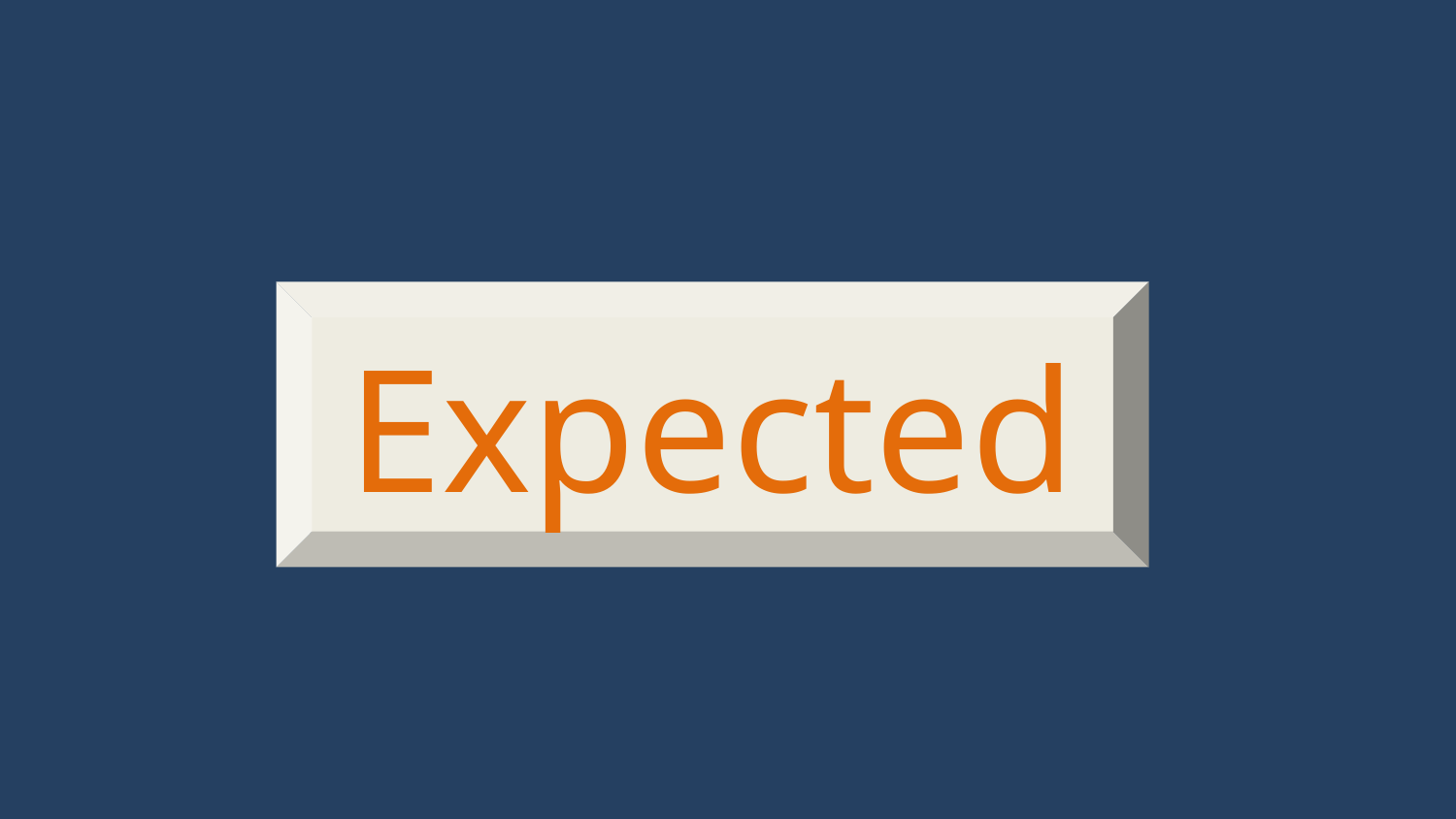

# Expected

## Slide 24
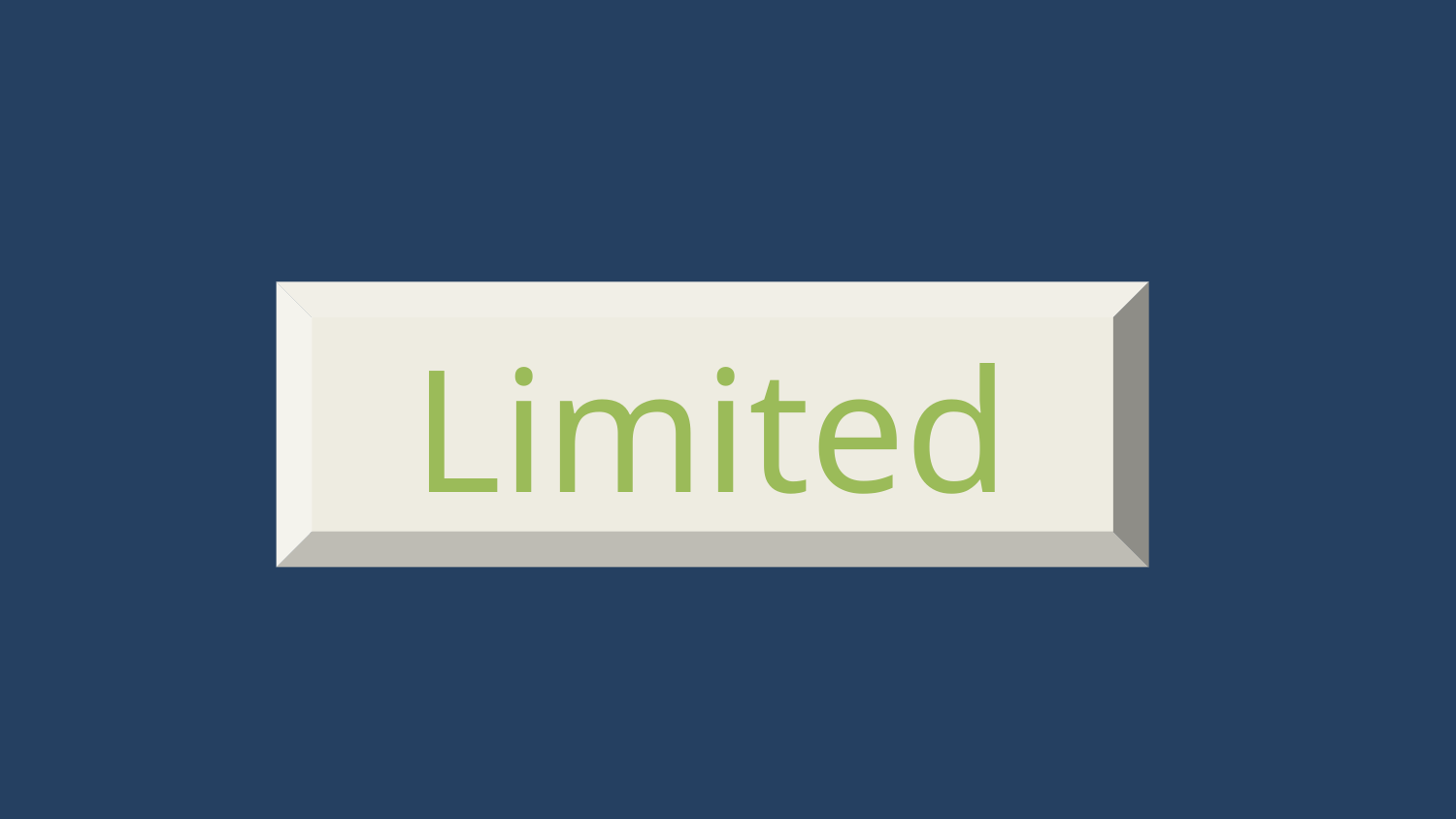

# Limited

## Slide 25
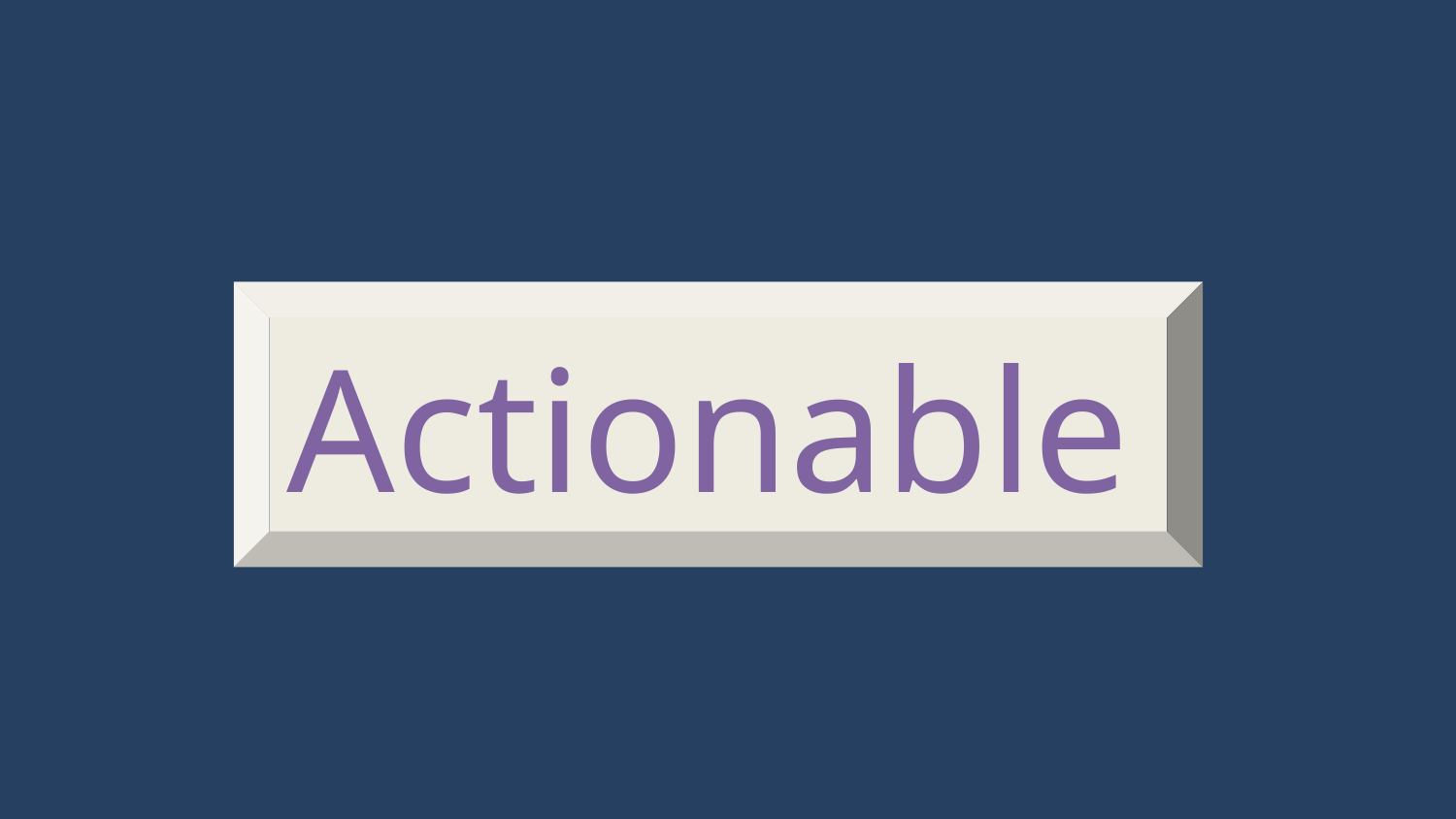

# Actionable

## Slide 26
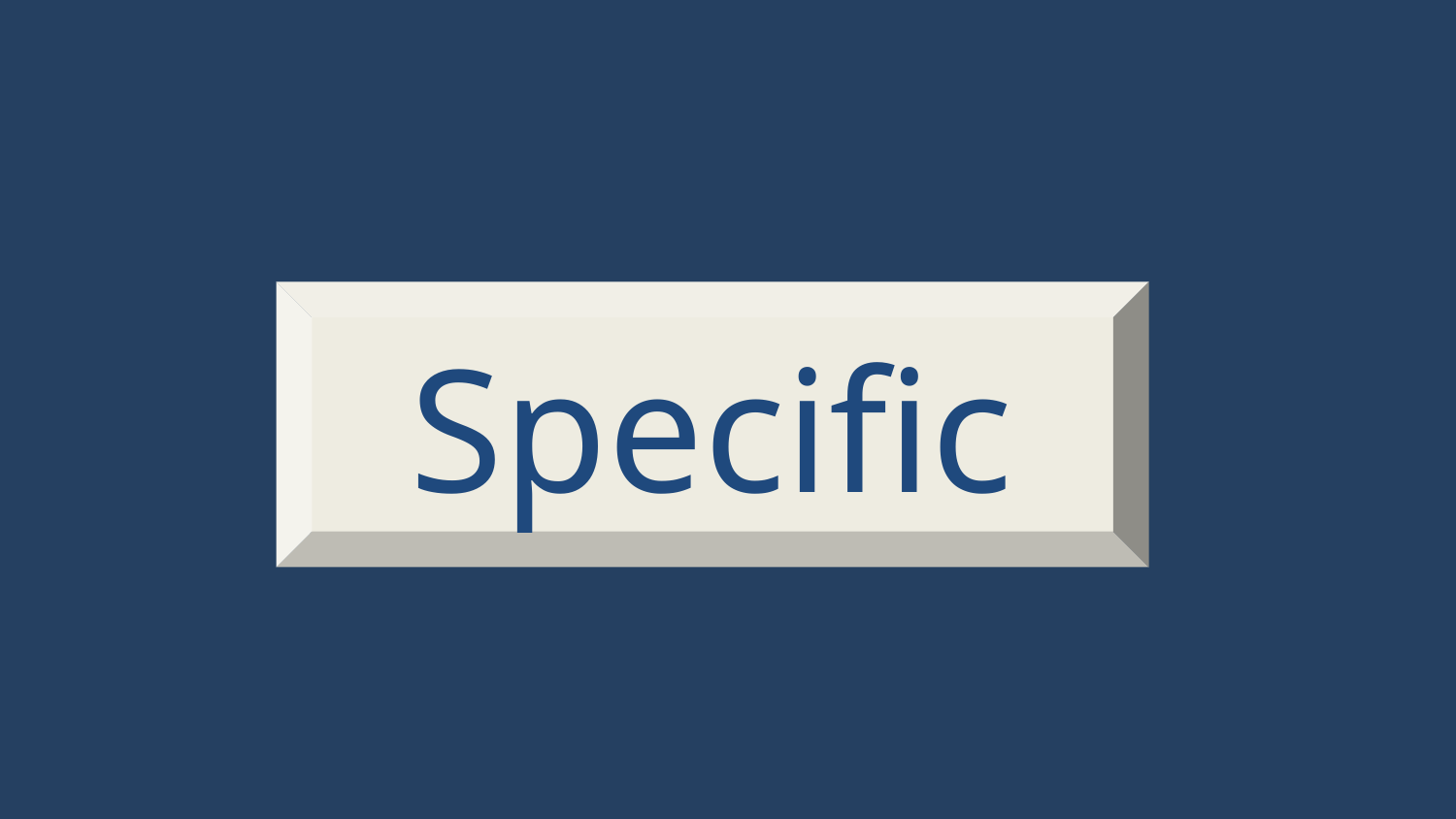

# Specific

## Slide 27
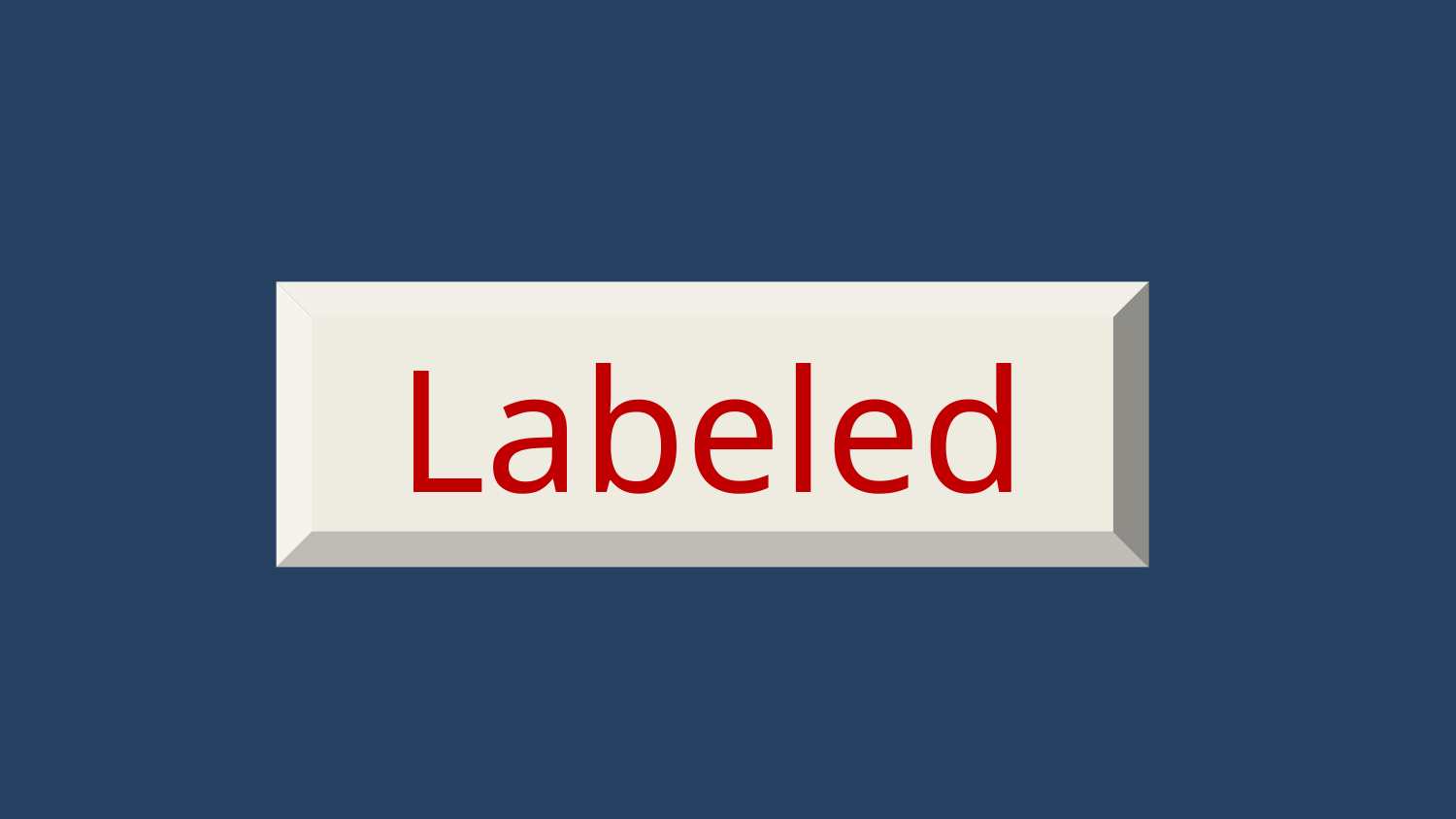

# Labeled

## Slide 28
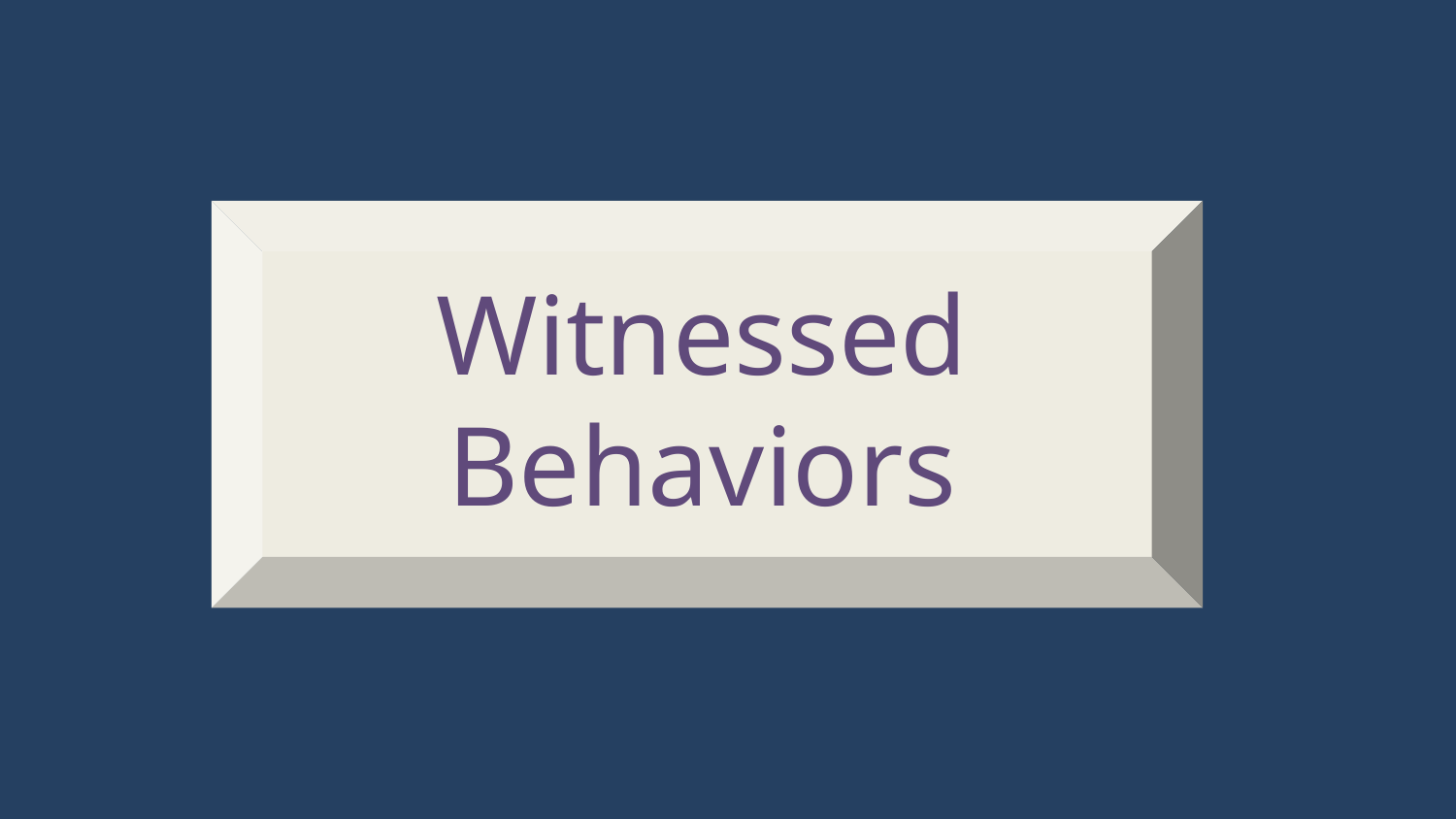

# Witnessed Behaviors

## Slide 29
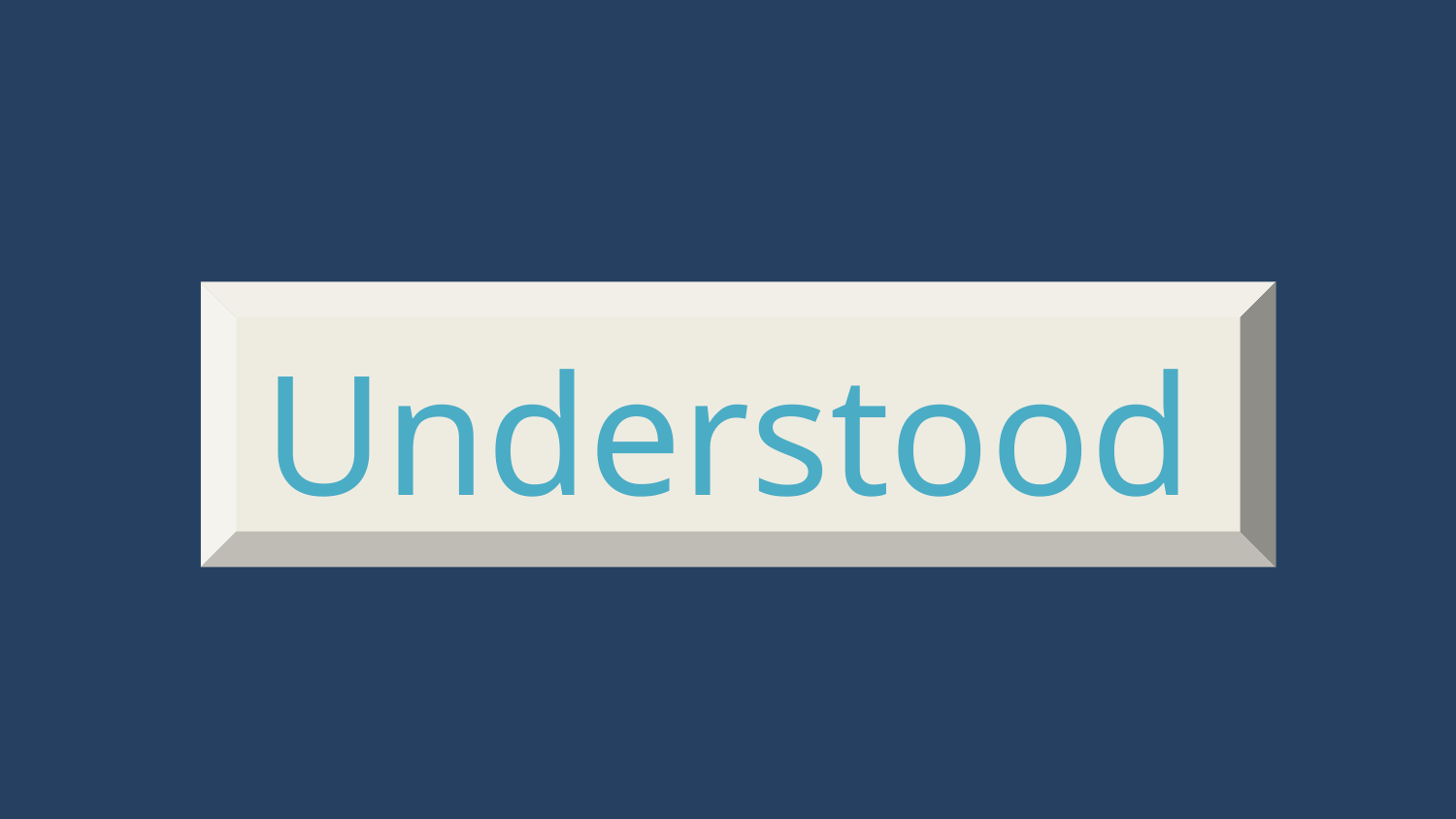

# Understood

## Slide 30
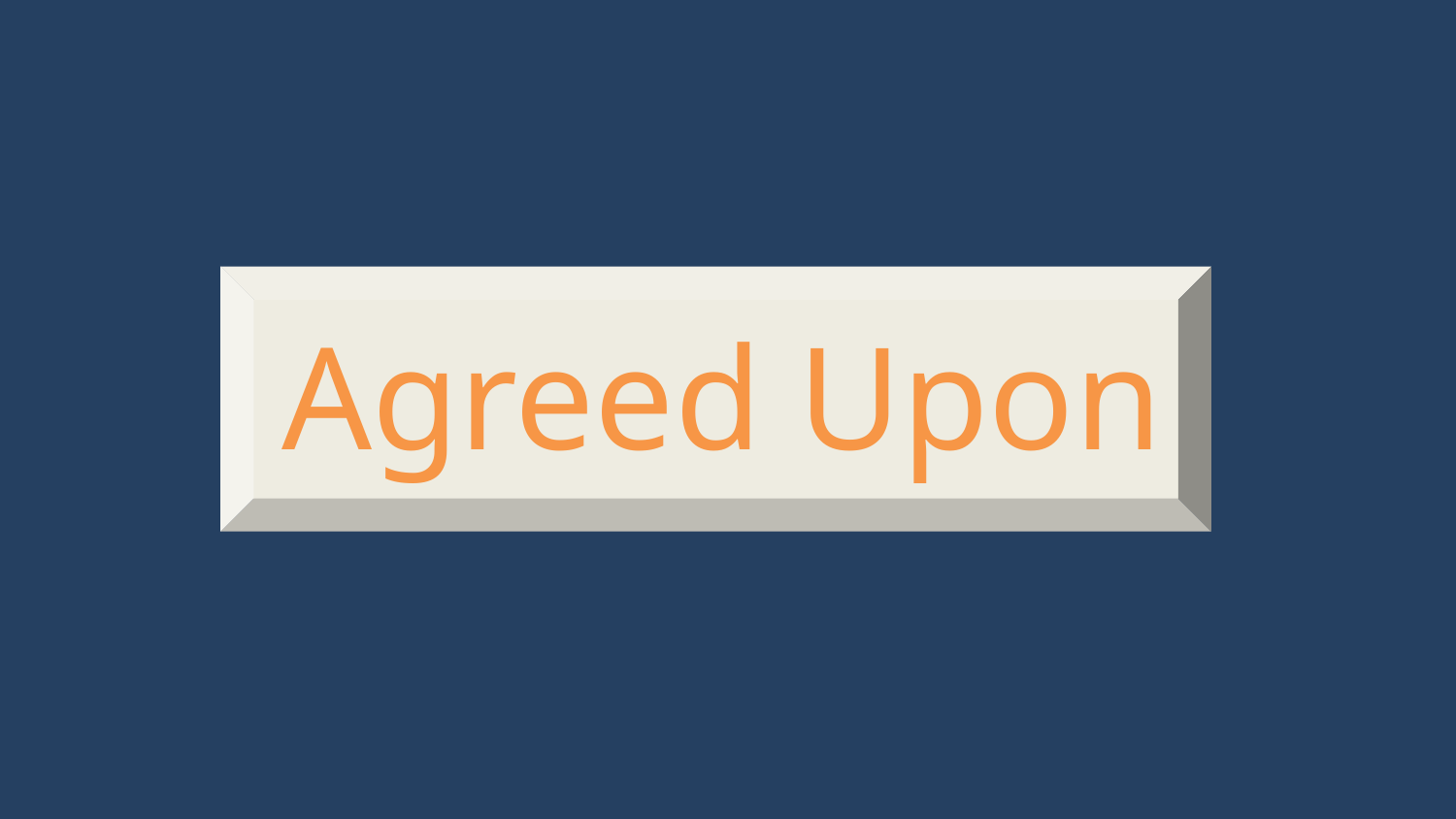

# Agreed Upon

## Slide 31
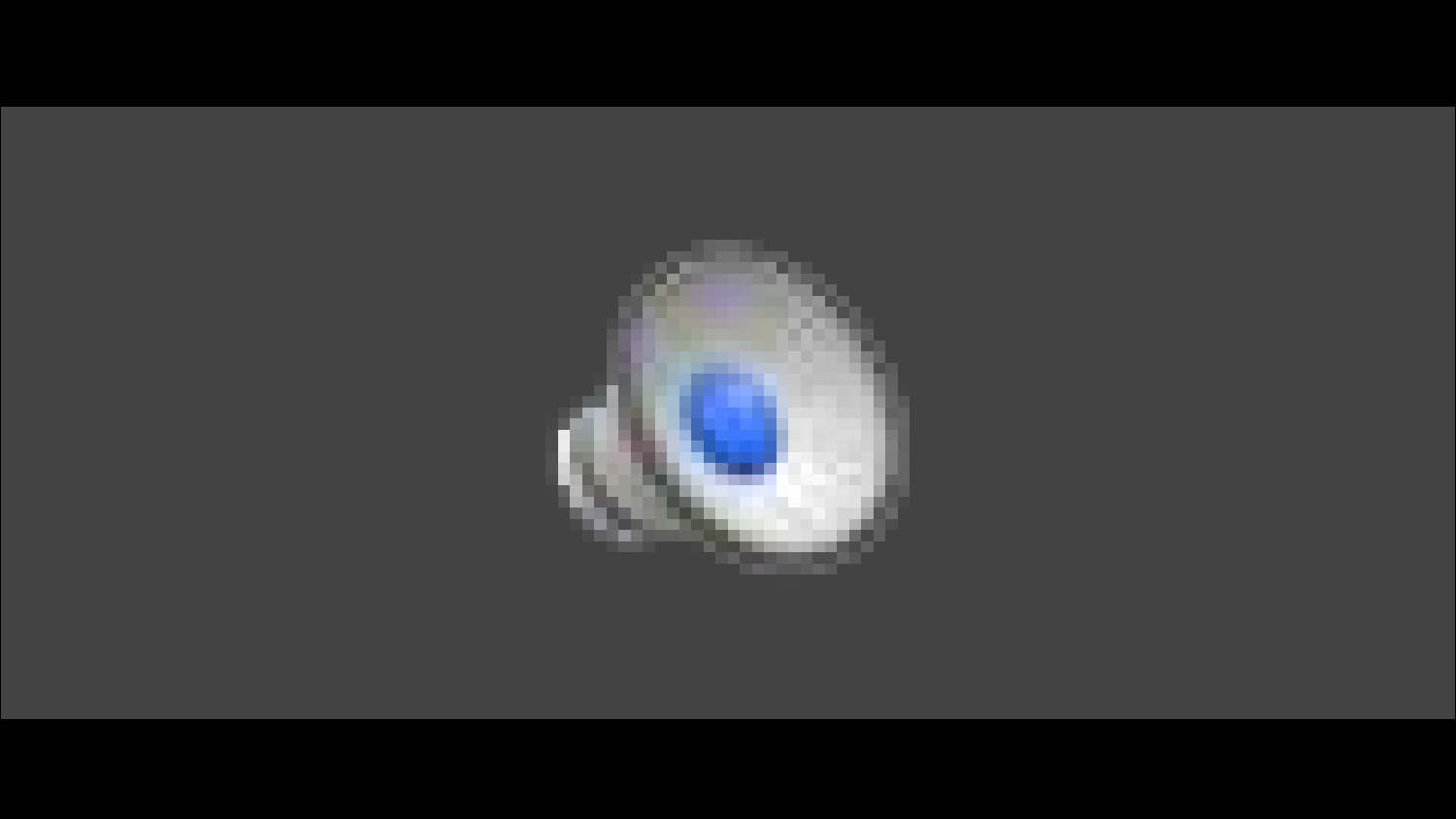

## Slide 32
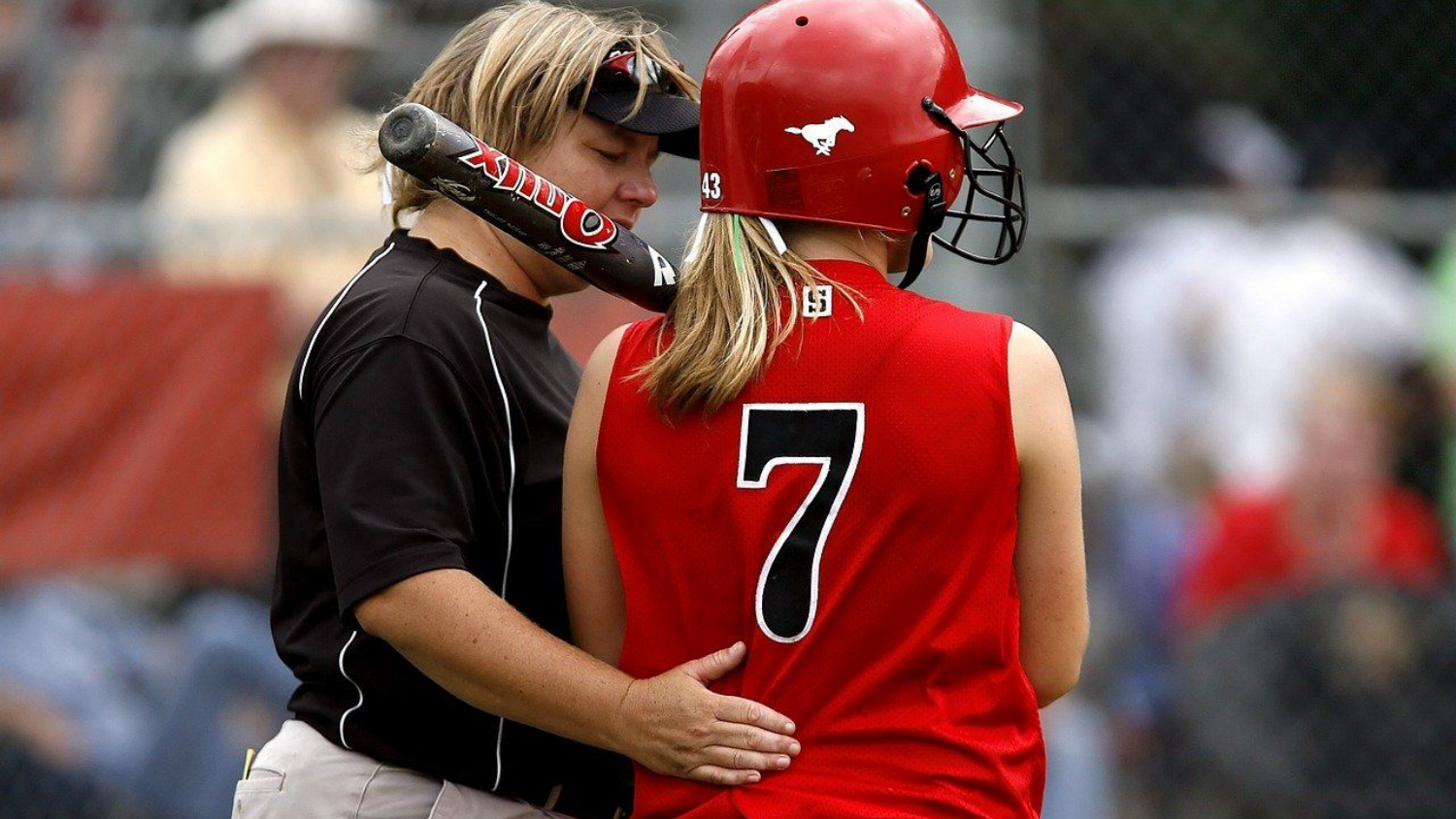

# Who’s This?

## Slide 33
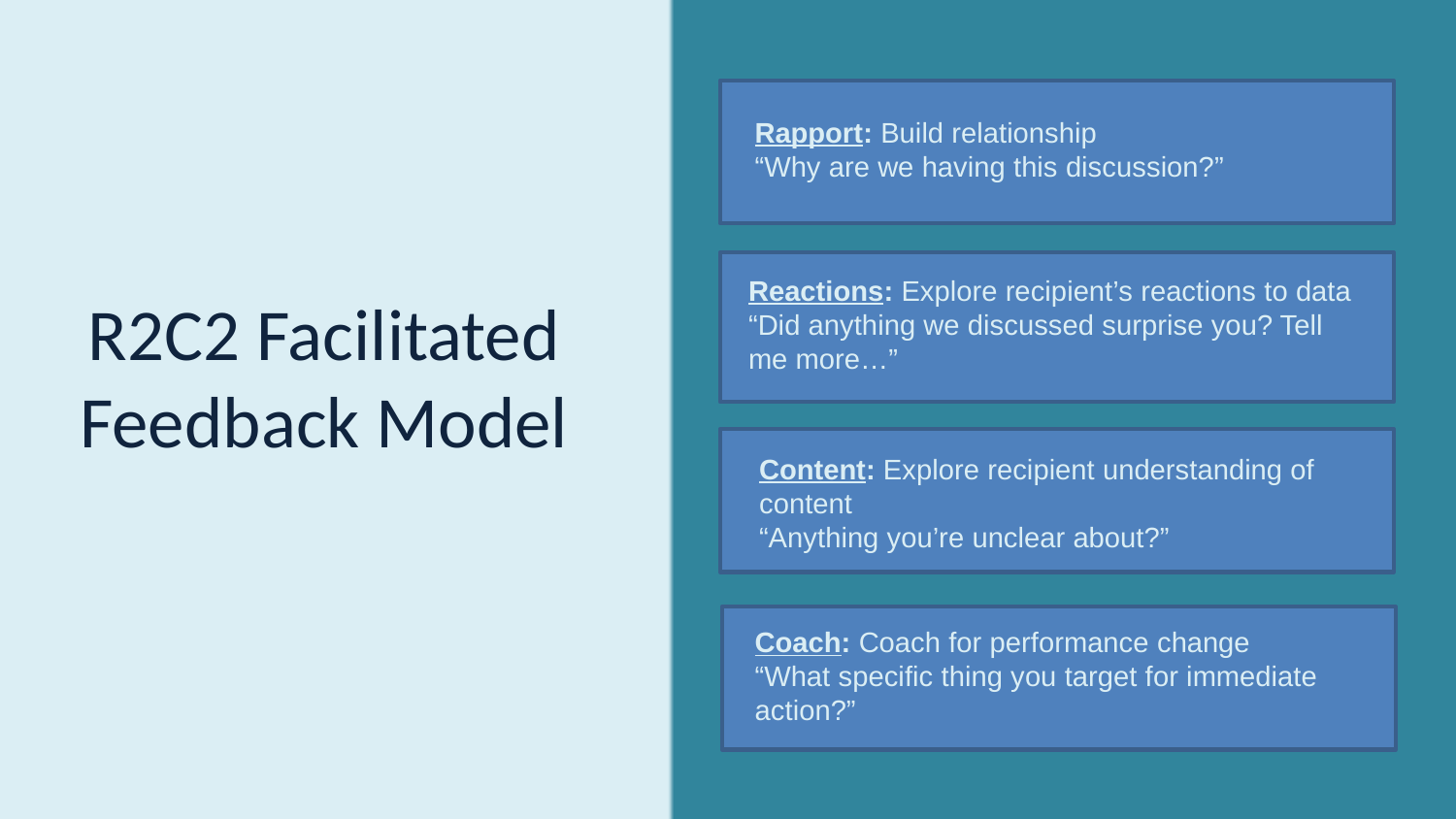

# R2C2 Facilitated Feedback Model
Rapport: Build relationship
“Why are we having this discussion?”
Reactions: Explore recipient’s reactions to data
“Did anything we discussed surprise you? Tell me more…”
Content: Explore recipient understanding of content
“Anything you’re unclear about?”
Coach: Coach for performance change
“What specific thing you target for immediate action?”

## Slide 34
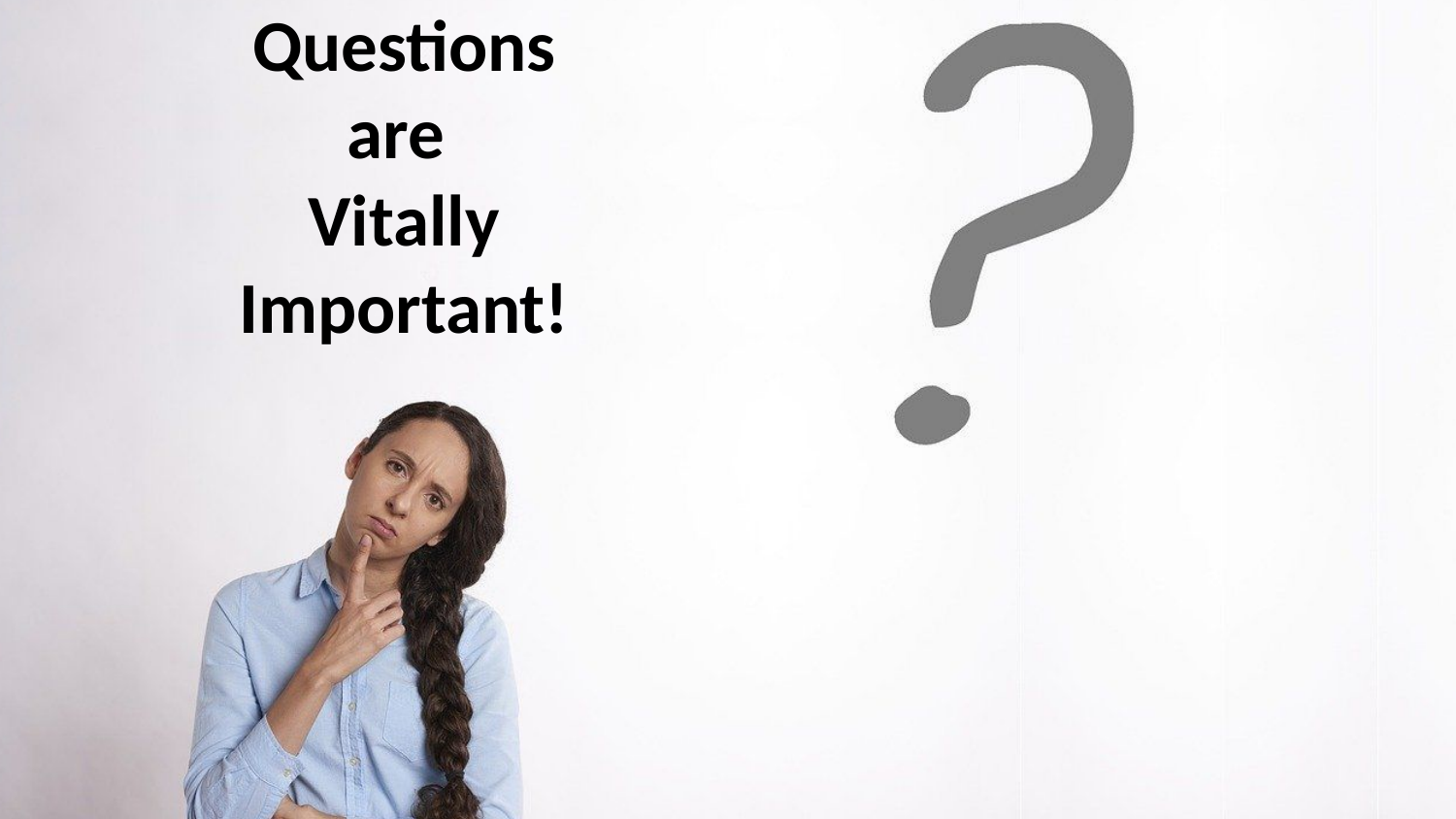

# Questions are Vitally Important!

## Slide 35
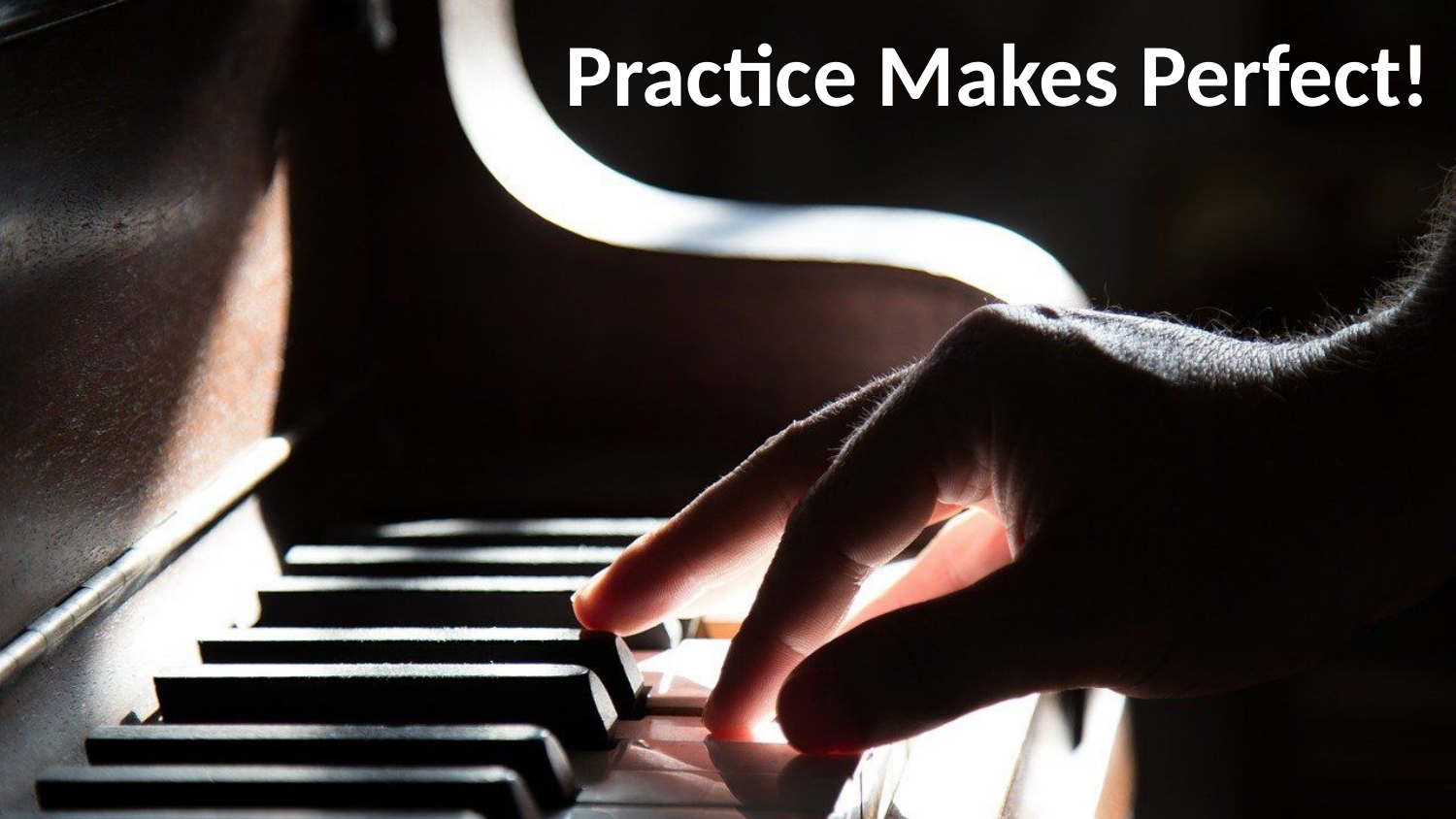

# Practice Makes Perfect!

## Slide 36
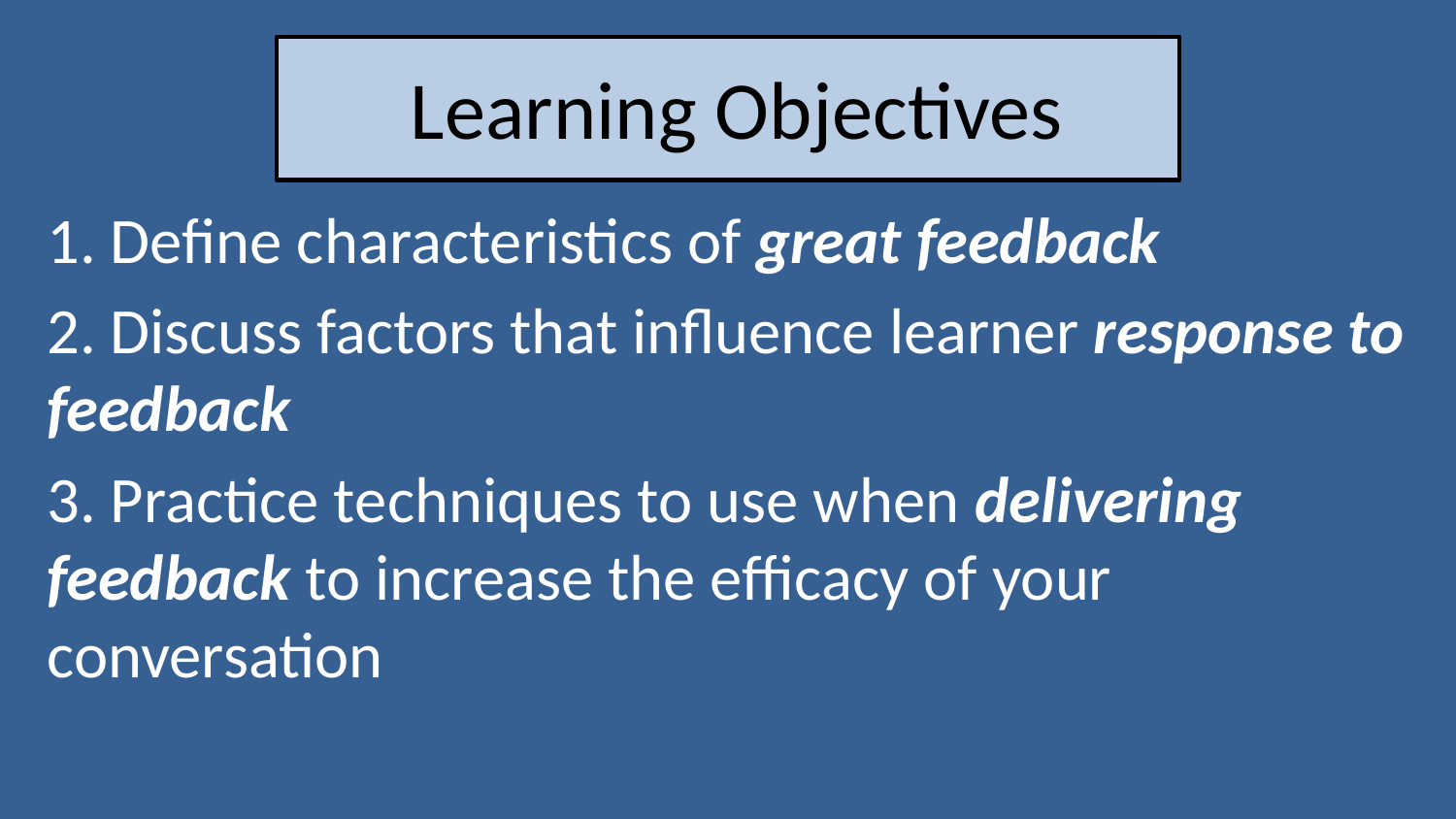

# Learning Objectives
1. Define characteristics of great feedback
2. Discuss factors that influence learner response to feedback
3. Practice techniques to use when delivering feedback to increase the efficacy of your conversation

## Slide 37
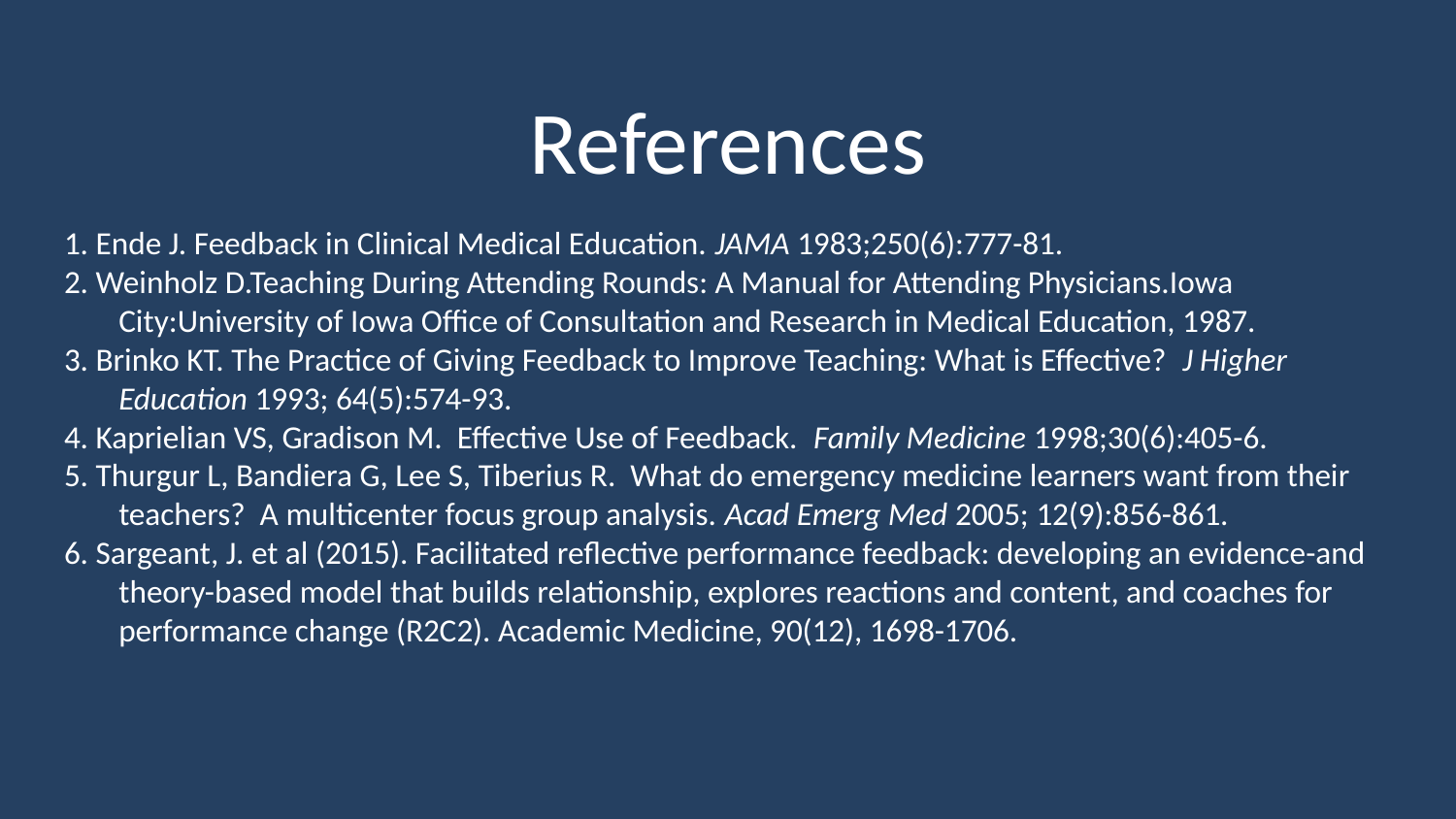

# References
1. Ende J. Feedback in Clinical Medical Education. JAMA 1983;250(6):777-81.
2. Weinholz D.Teaching During Attending Rounds: A Manual for Attending Physicians.Iowa City:University of Iowa Office of Consultation and Research in Medical Education, 1987.
3. Brinko KT. The Practice of Giving Feedback to Improve Teaching: What is Effective? J Higher Education 1993; 64(5):574-93.
4. Kaprielian VS, Gradison M. Effective Use of Feedback. Family Medicine 1998;30(6):405-6.
5. Thurgur L, Bandiera G, Lee S, Tiberius R. What do emergency medicine learners want from their teachers? A multicenter focus group analysis. Acad Emerg Med 2005; 12(9):856-861.
6. Sargeant, J. et al (2015). Facilitated reflective performance feedback: developing an evidence-and theory-based model that builds relationship, explores reactions and content, and coaches for performance change (R2C2). Academic Medicine, 90(12), 1698-1706.
