# Supplementary material for: A Novel Virtual Emergency Medicine Residents-as-Teachers (RAT) Curriculum
Source: J Educ Teach Emerg Med. 2021 Jul 15;6(3):C9–C63. doi: 10.21980/J86S71 (PMC10332683; doi:10.21980/J86S71)
Supplement: Supplementary file 5 — Please see associated PowerPoint file [file jetem-6-3-c8-appendix4c.pptx]

## Slide 1
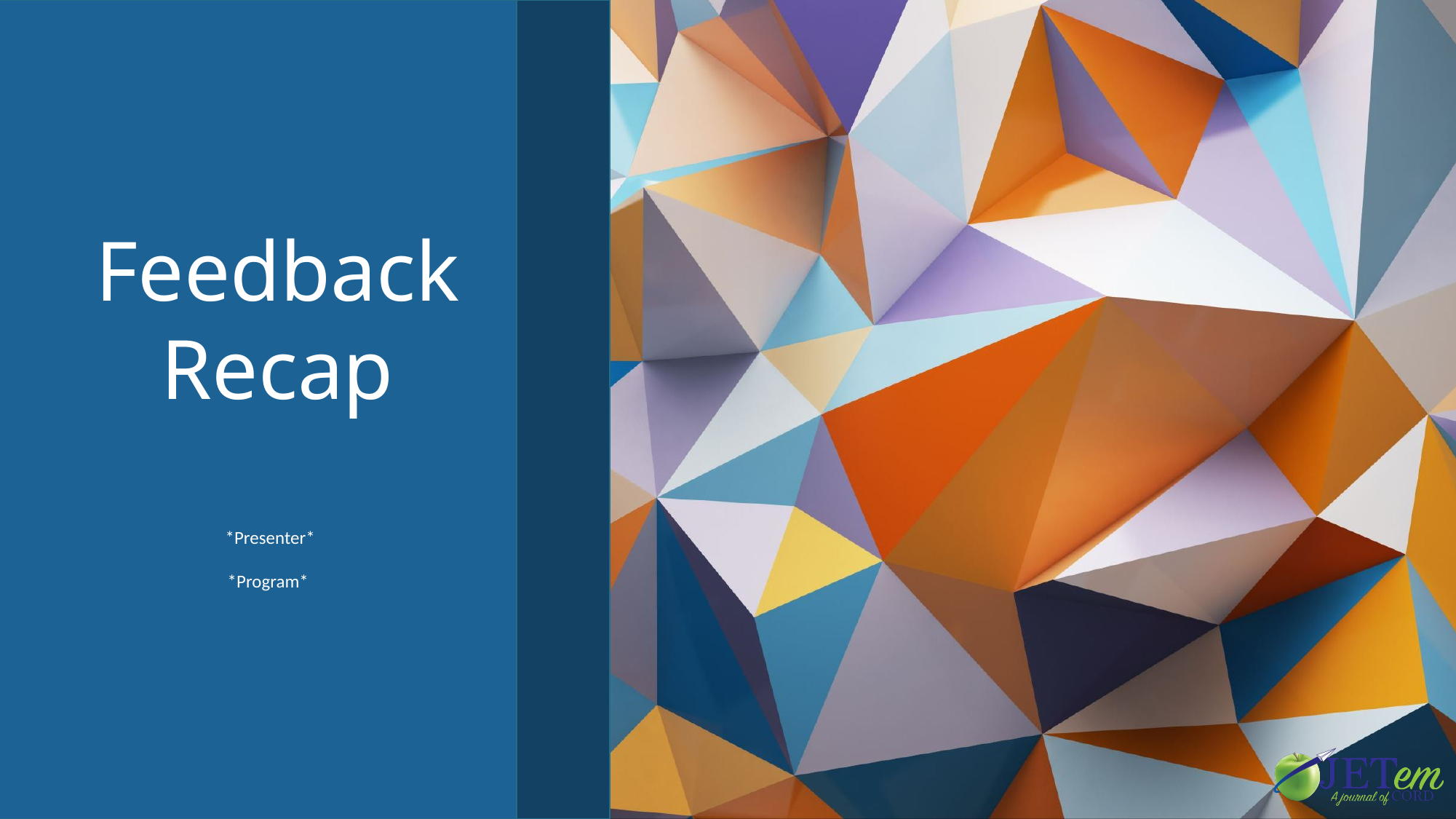

Feedback Recap
*Presenter*
*Program*

## Slide 2
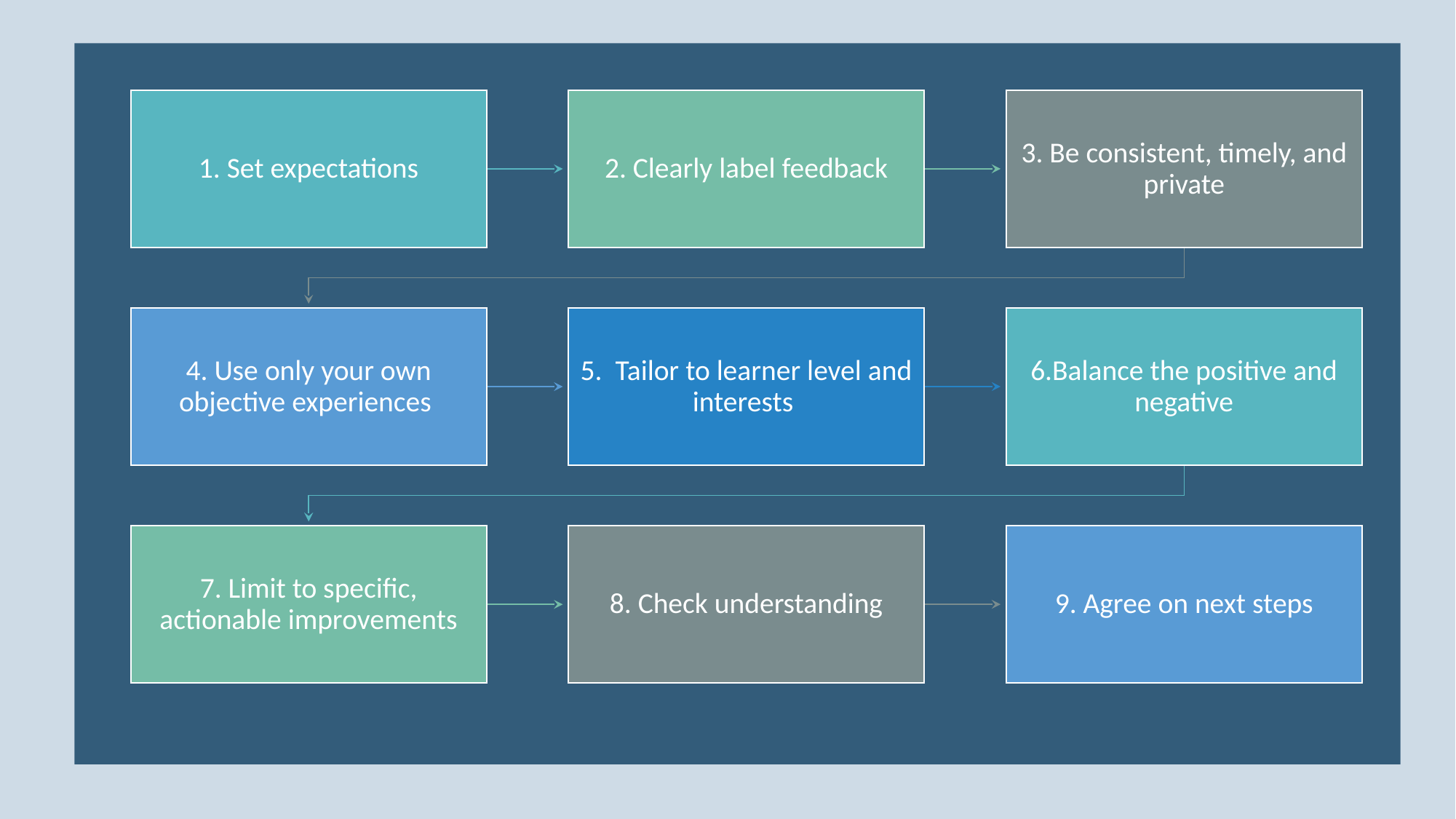

1. Set expectations
2. Clearly label feedback
3. Be consistent, timely, and private
4. Use only your own objective experiences
5. Tailor to learner level and interests
6.Balance the positive and negative
7. Limit to specific, actionable improvements
8. Check understanding
9. Agree on next steps
