# Supplementary material for: A Novel Virtual Emergency Medicine Residents-as-Teachers (RAT) Curriculum
Source: J Educ Teach Emerg Med. 2021 Jul 15;6(3):C9–C63. doi: 10.21980/J86S71 (PMC10332683; doi:10.21980/J86S71)
Supplement: Supplementary file 6 — Please see associated PowerPoint file [file jetem-6-3-c8-Appendix4d.pptx]

## Slide 1
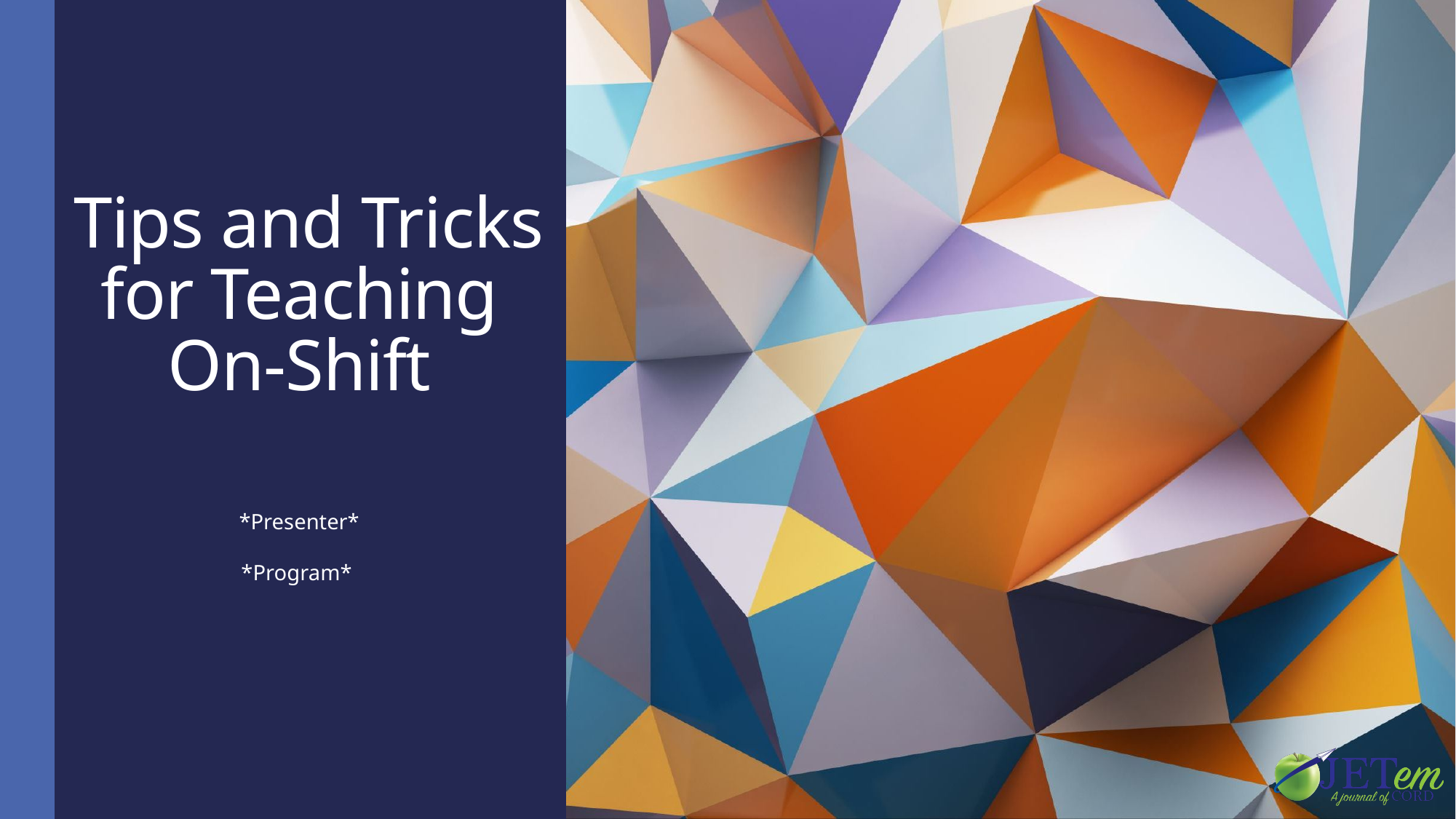

# Tips and Tricks for Teaching On-Shift
*Presenter*
*Program*

## Slide 2
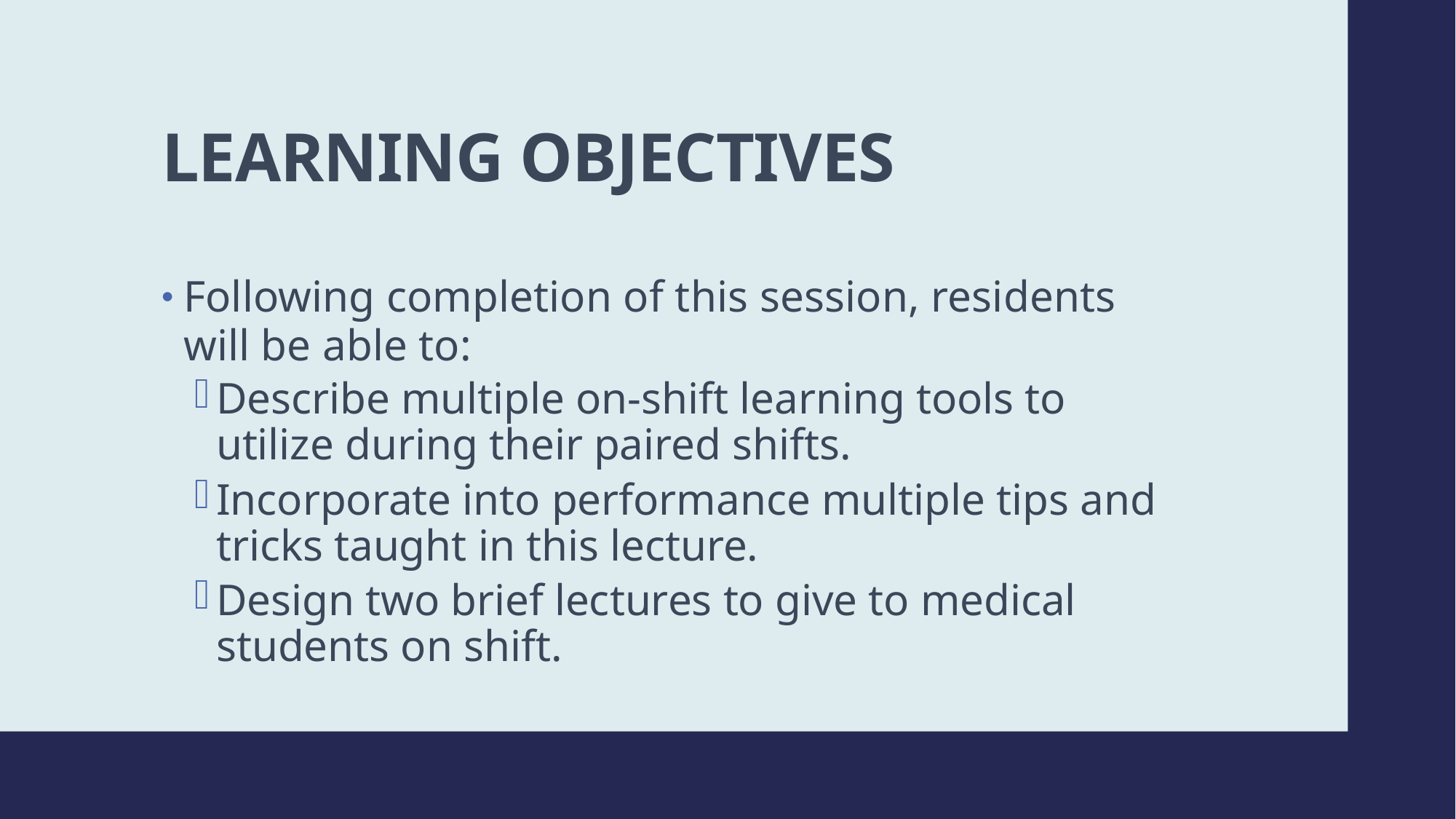

# LEARNING OBJECTIVES
Following completion of this session, residents will be able to:
Describe multiple on-shift learning tools to utilize during their paired shifts.
Incorporate into performance multiple tips and tricks taught in this lecture.
Design two brief lectures to give to medical students on shift.

## Slide 3
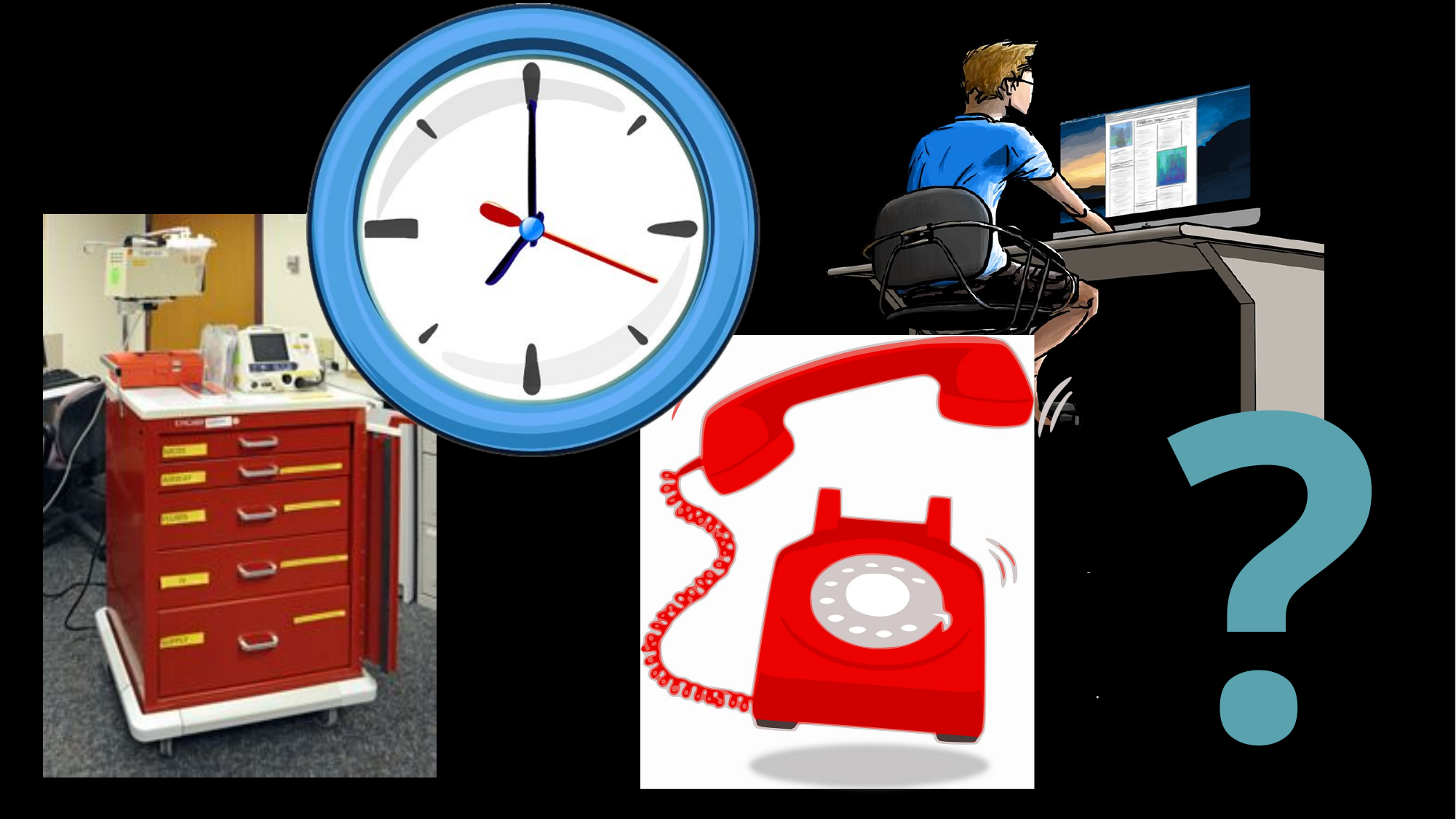

?

## Slide 4
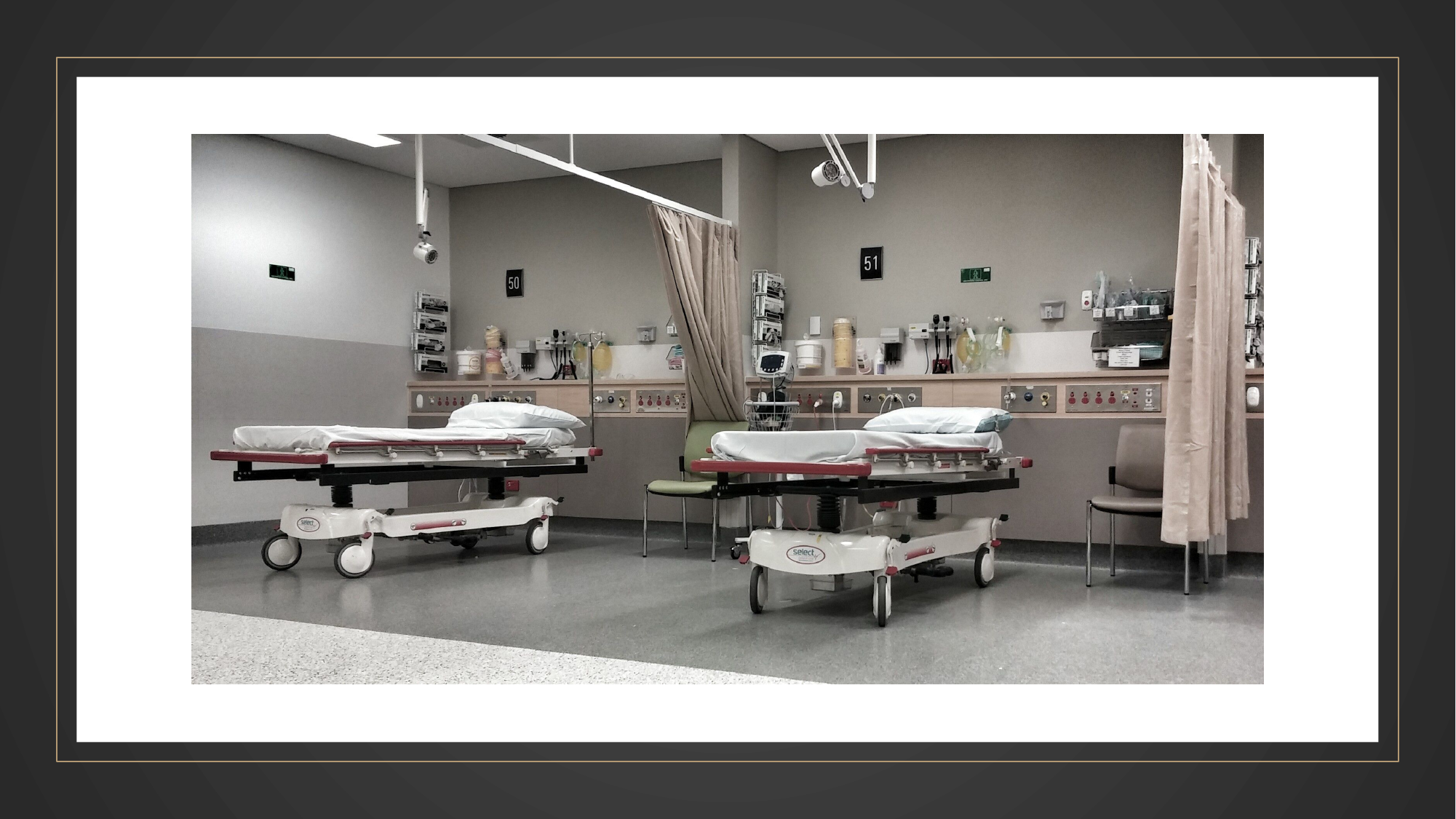

## Slide 5
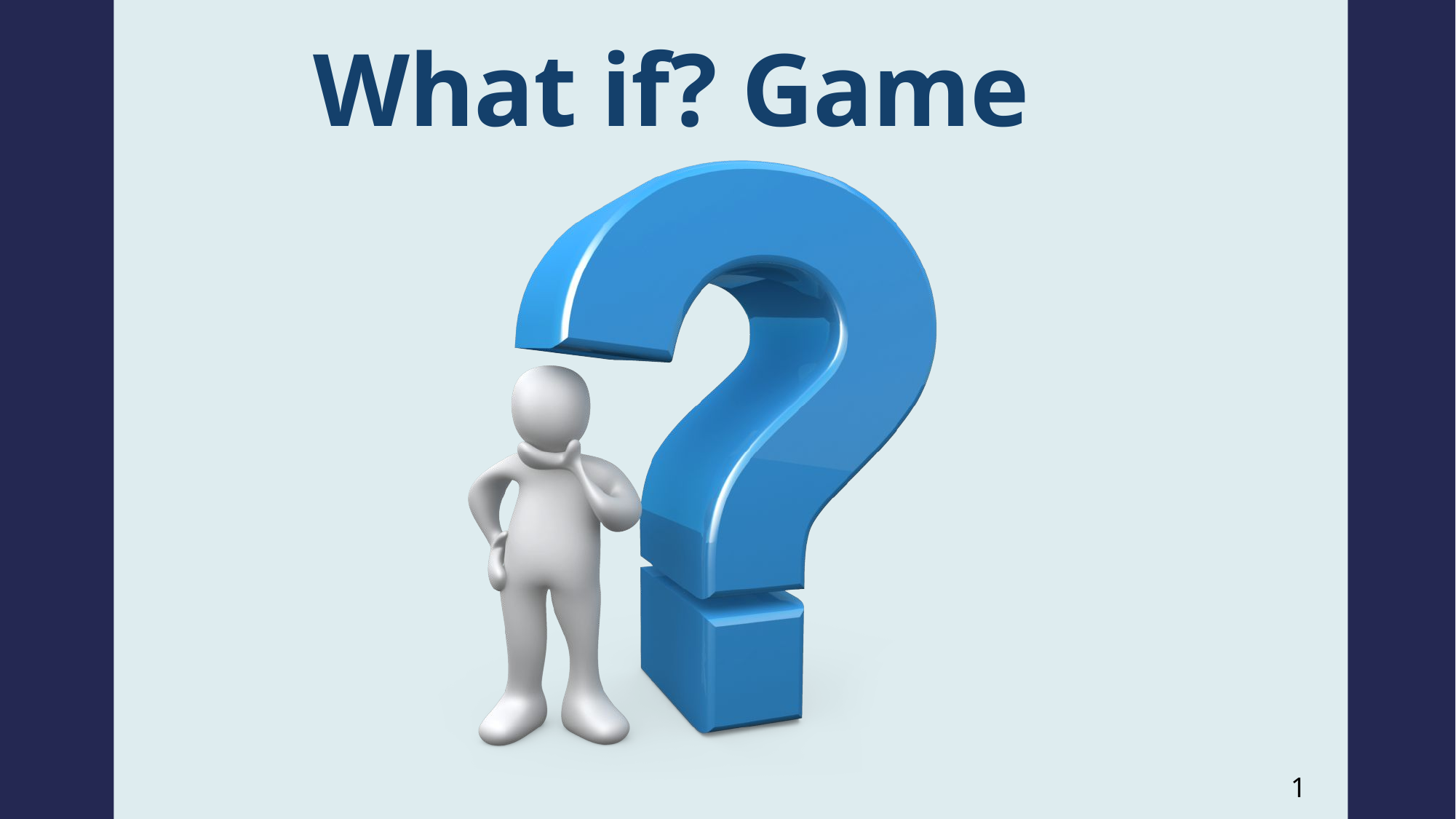

# What if? Game
1

## Slide 6
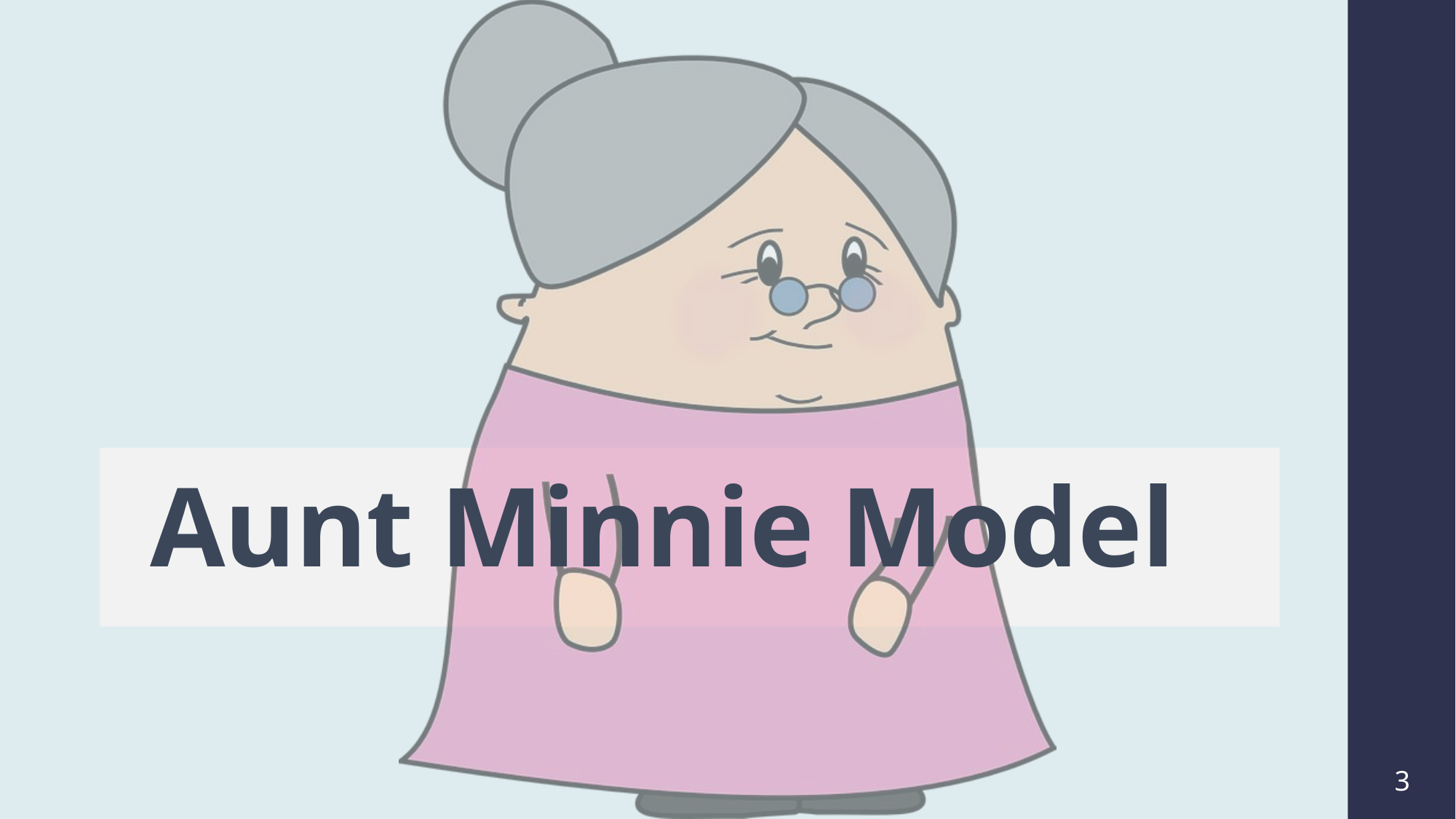

# Aunt Minnie Model
3

## Slide 7
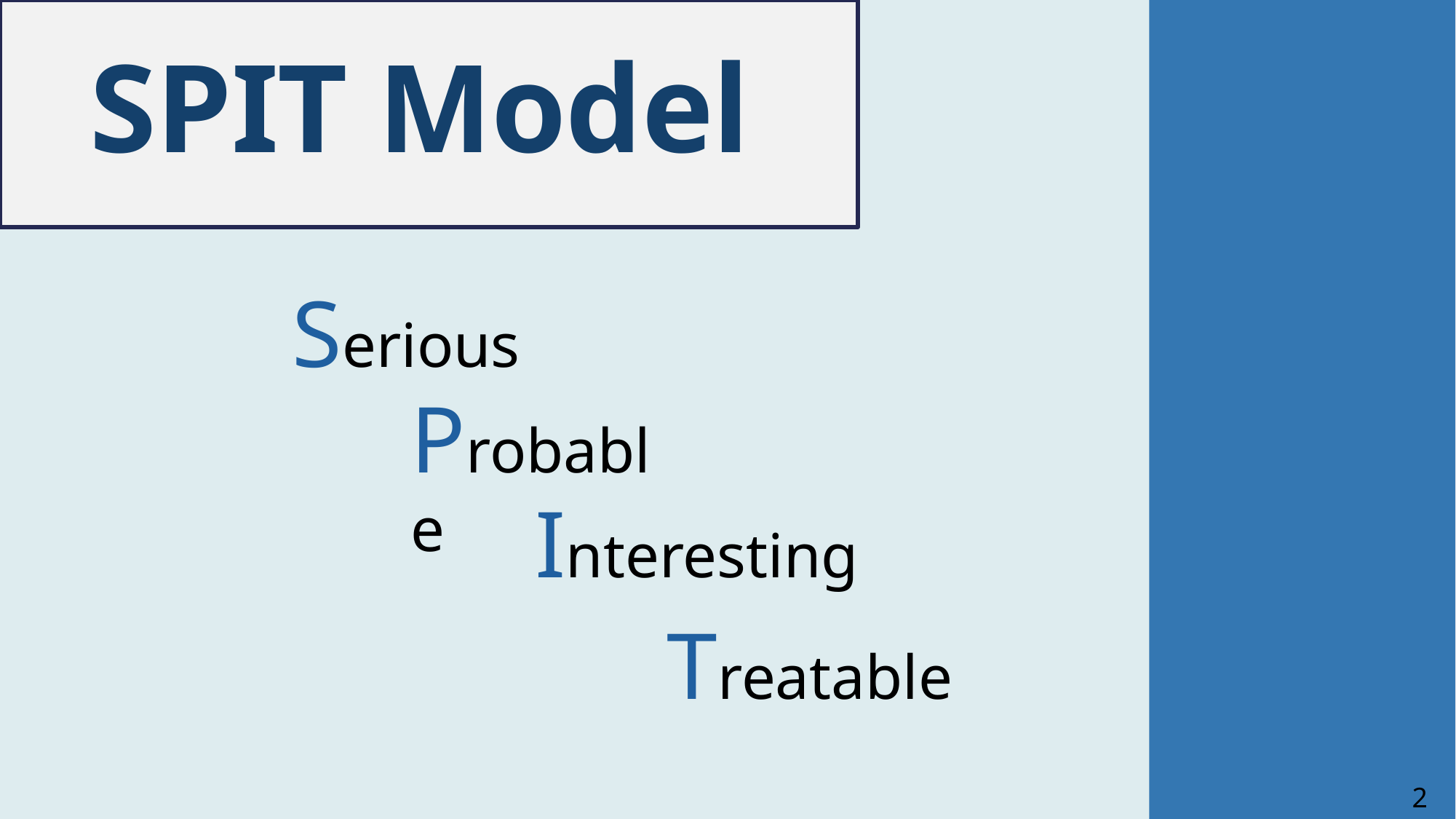

# SPIT Model
Serious
Probable
Interesting
Treatable
2

## Slide 8
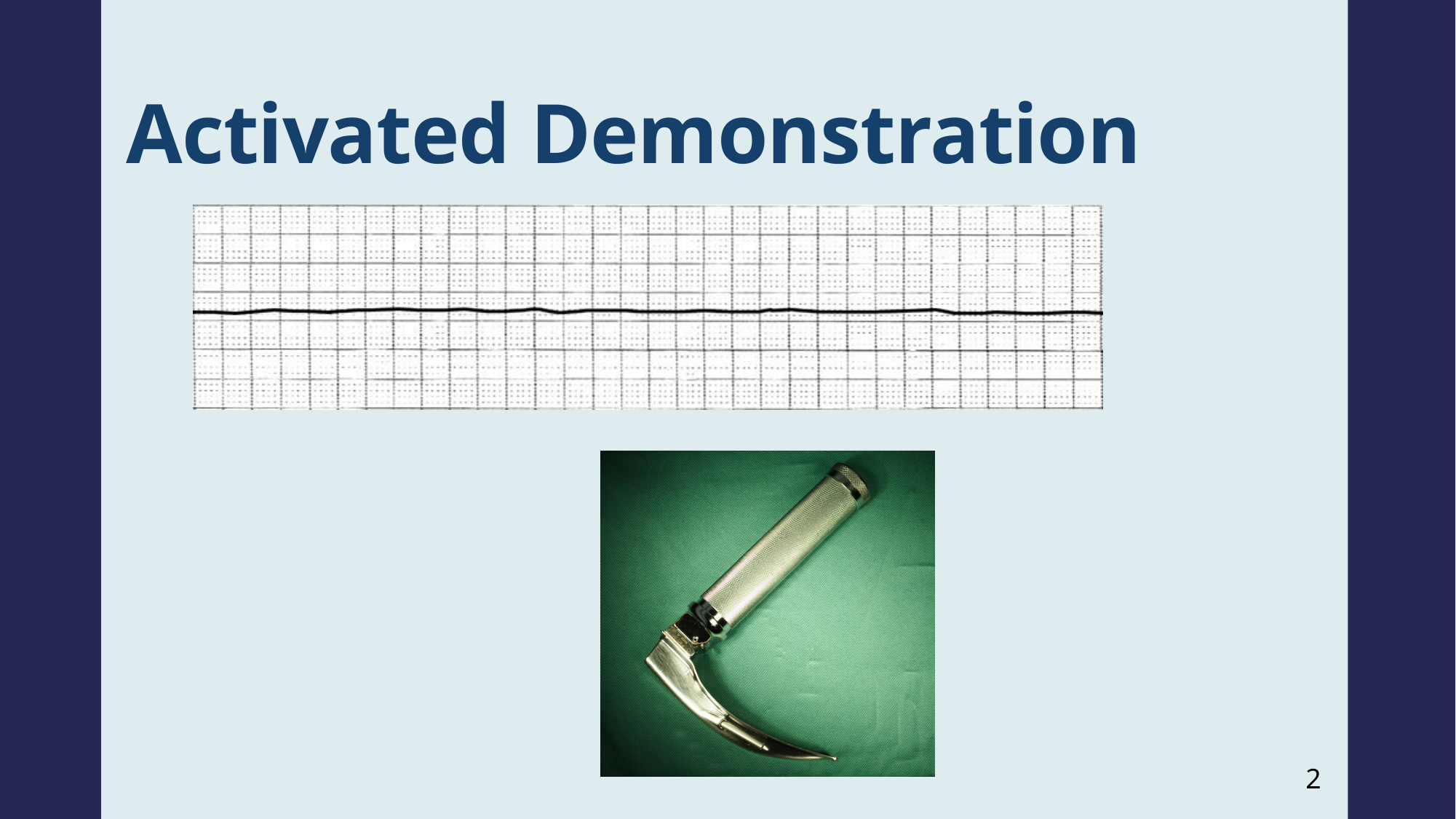

# Activated Demonstration
2

## Slide 9
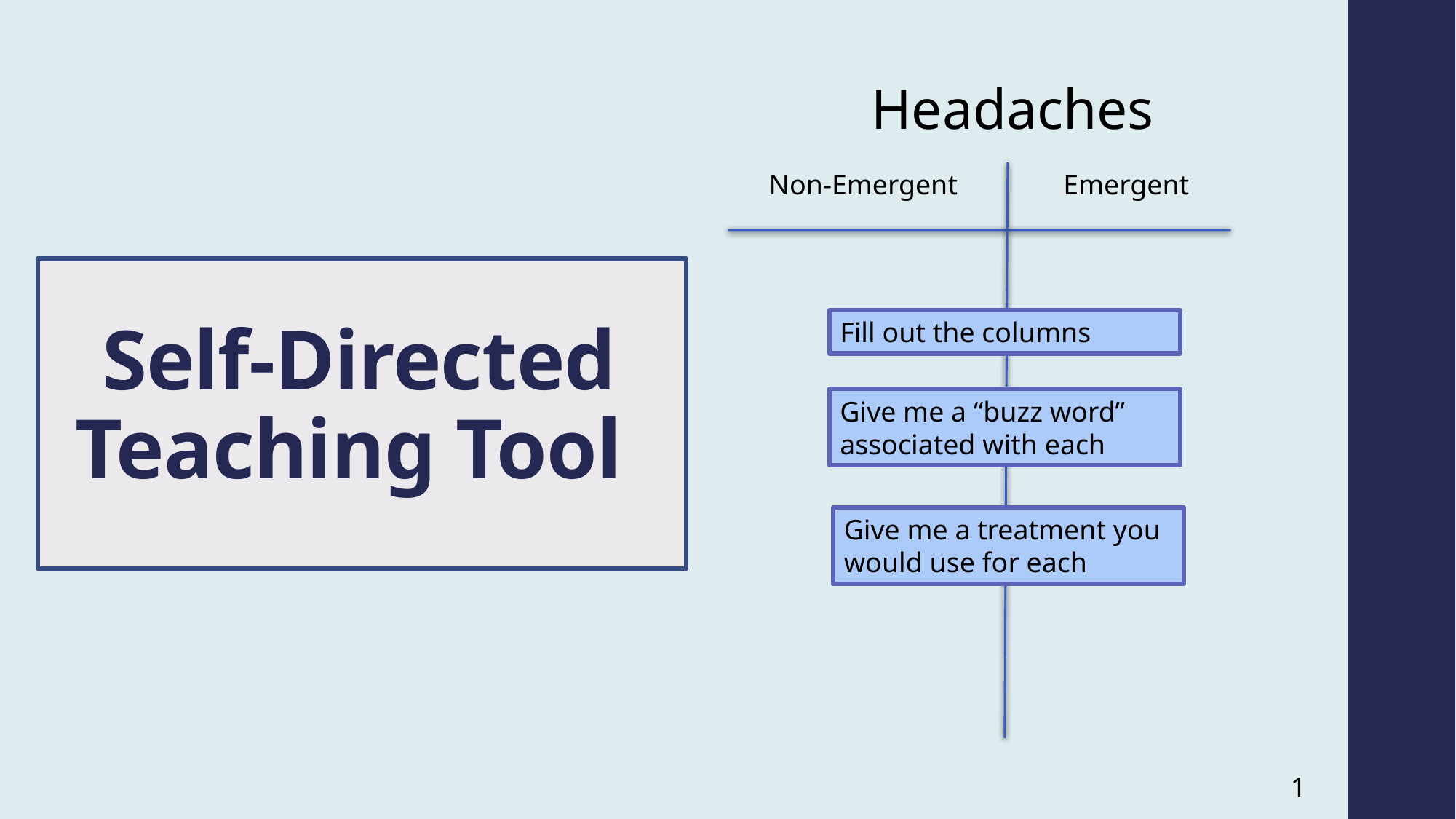

Headaches
Non-Emergent
Emergent
Fill out the columns
# Self-Directed Teaching Tool
Give me a “buzz word” associated with each
Give me a treatment you would use for each
1

## Slide 10
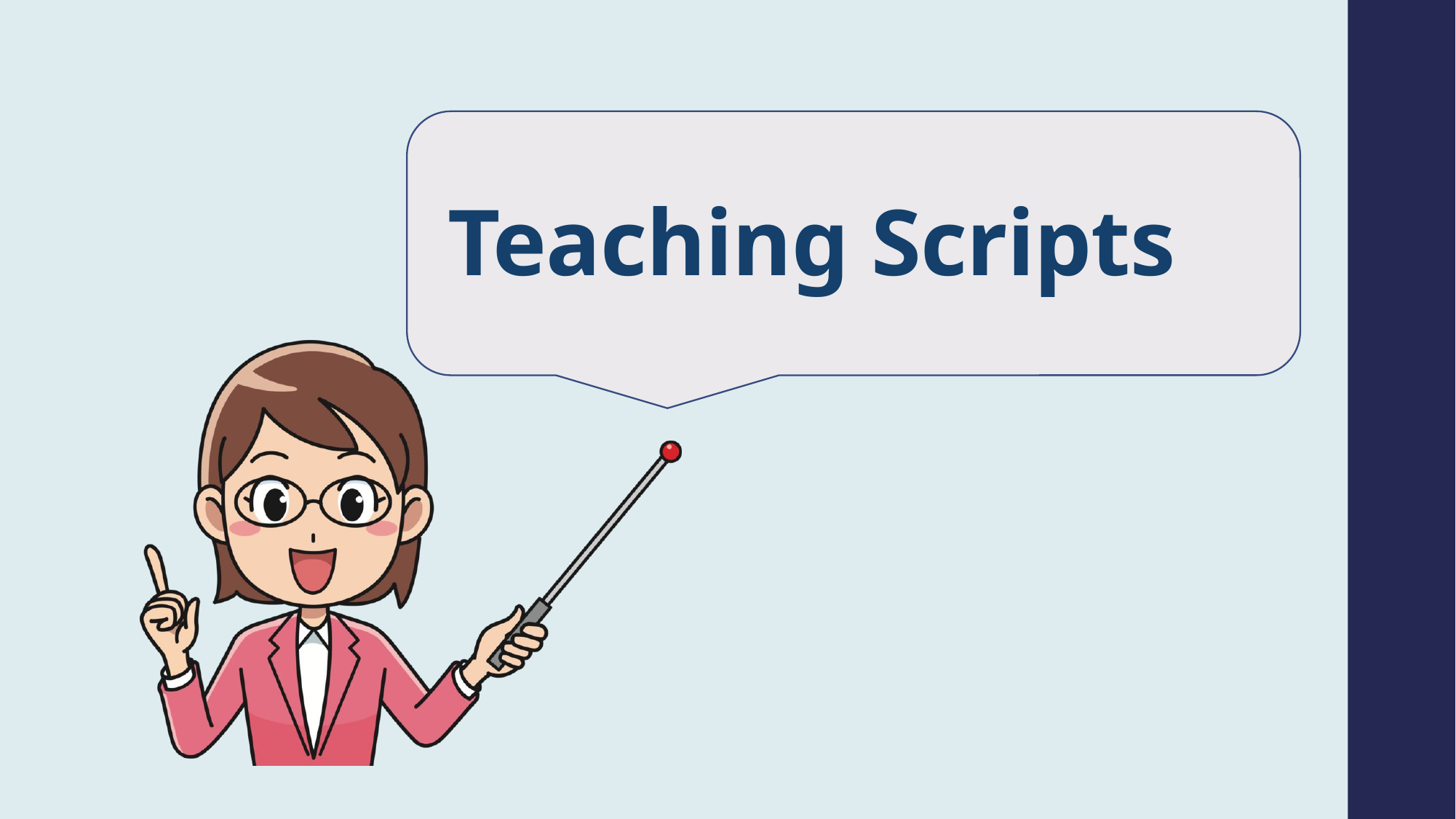

# Teaching Scripts

## Slide 11
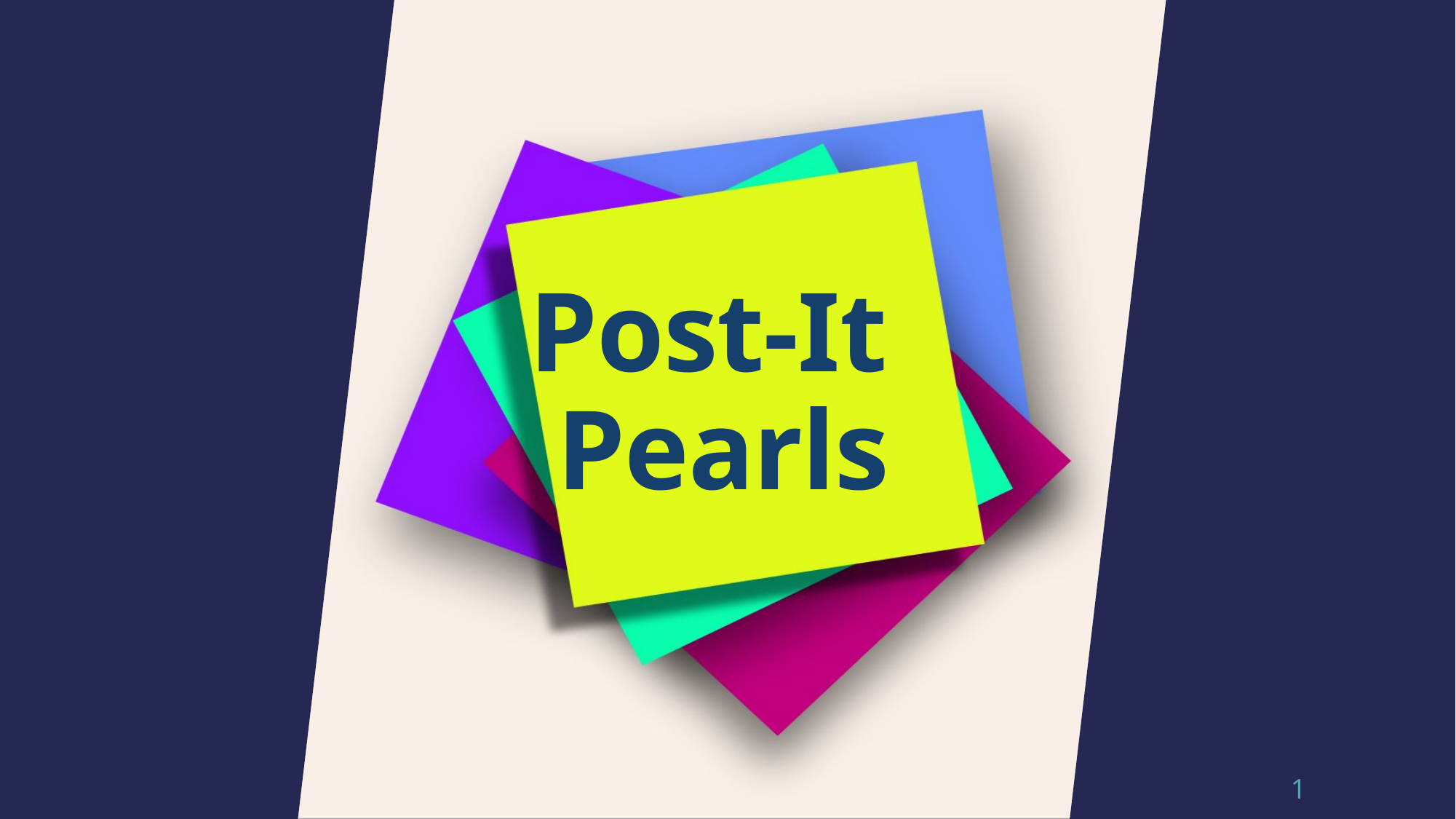

# Post-It Pearls
1
1

## Slide 12
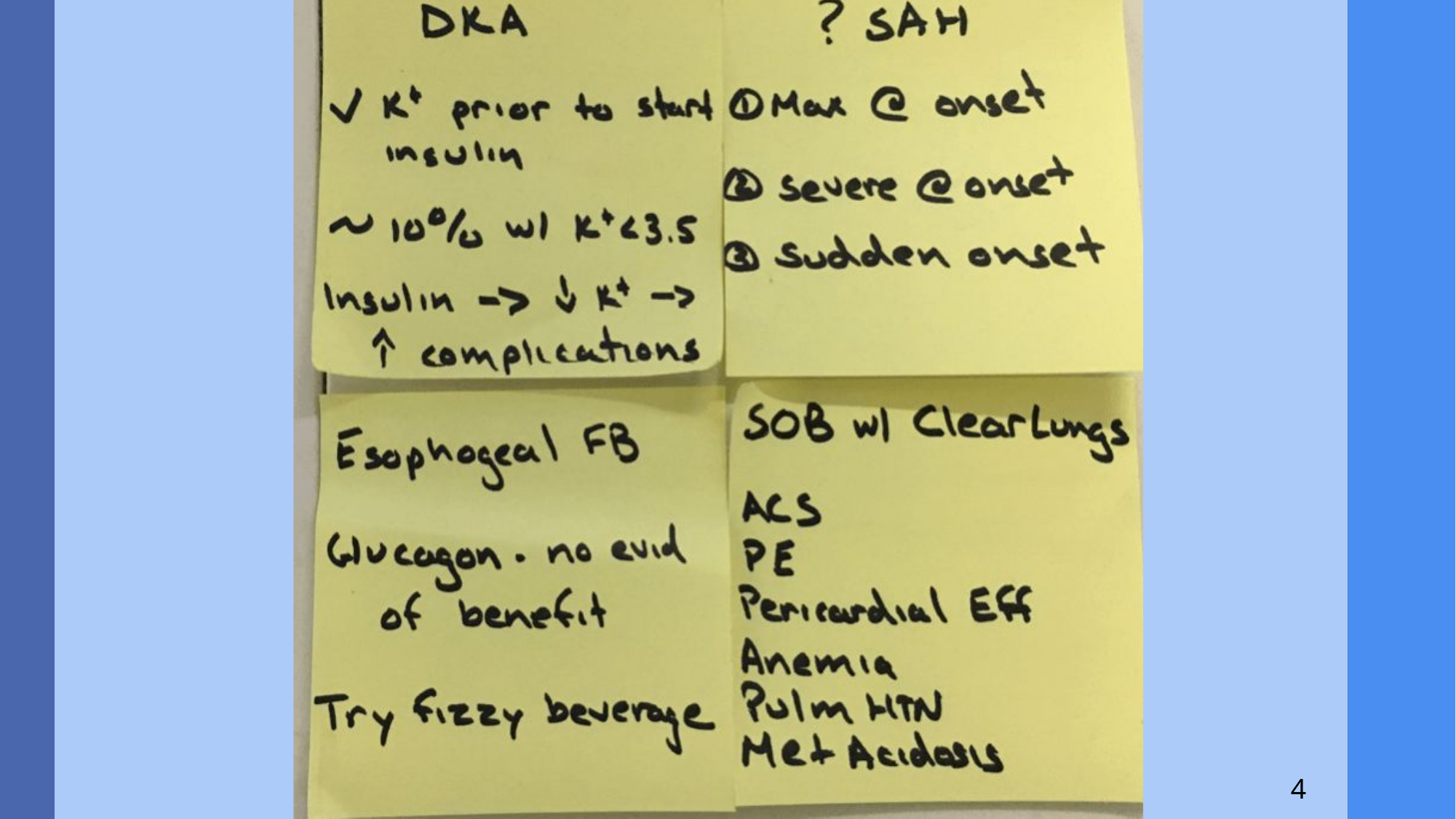

4

## Slide 13
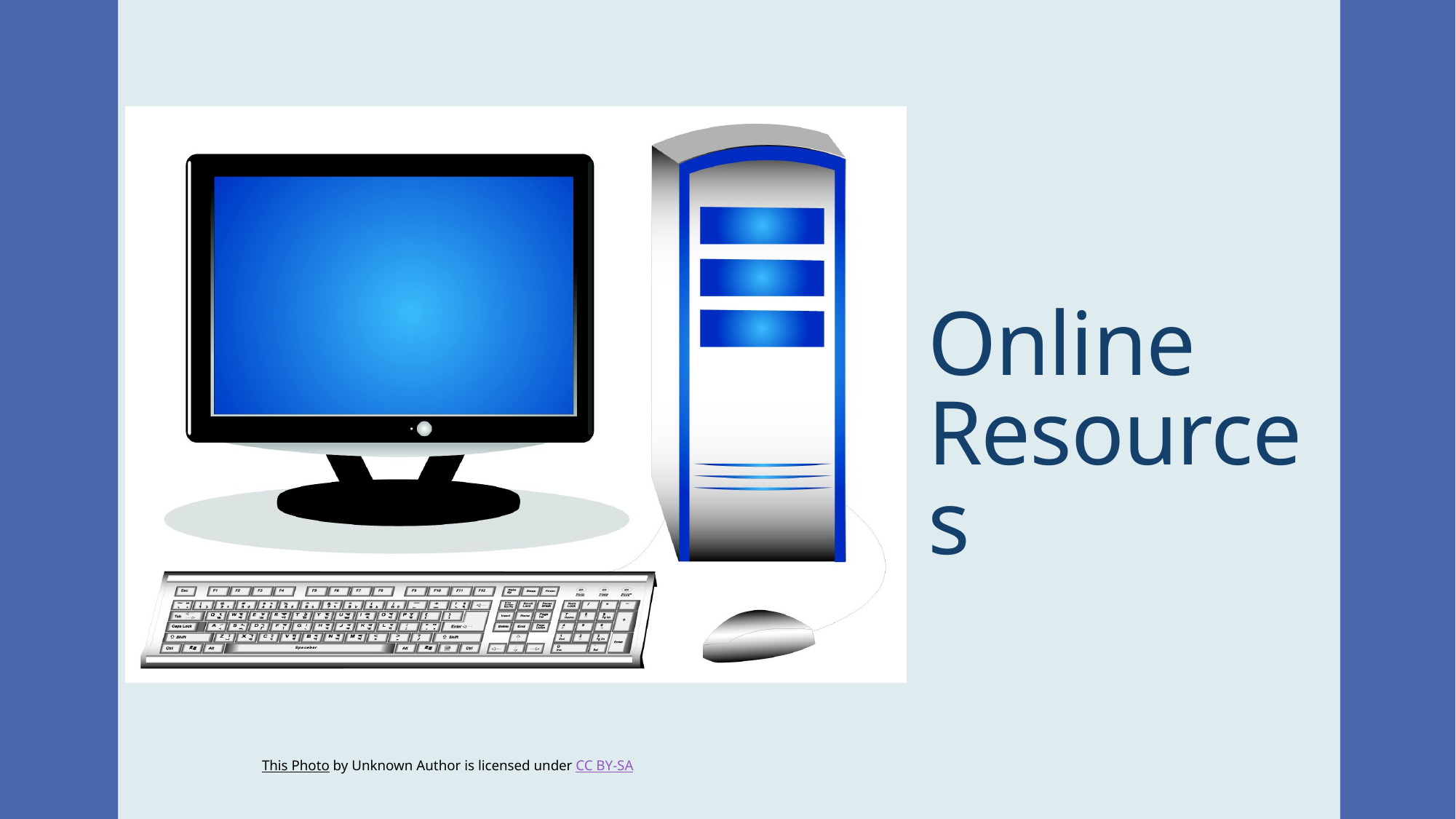

# Online Resources
This Photo by Unknown Author is licensed under CC BY-SA

## Slide 14
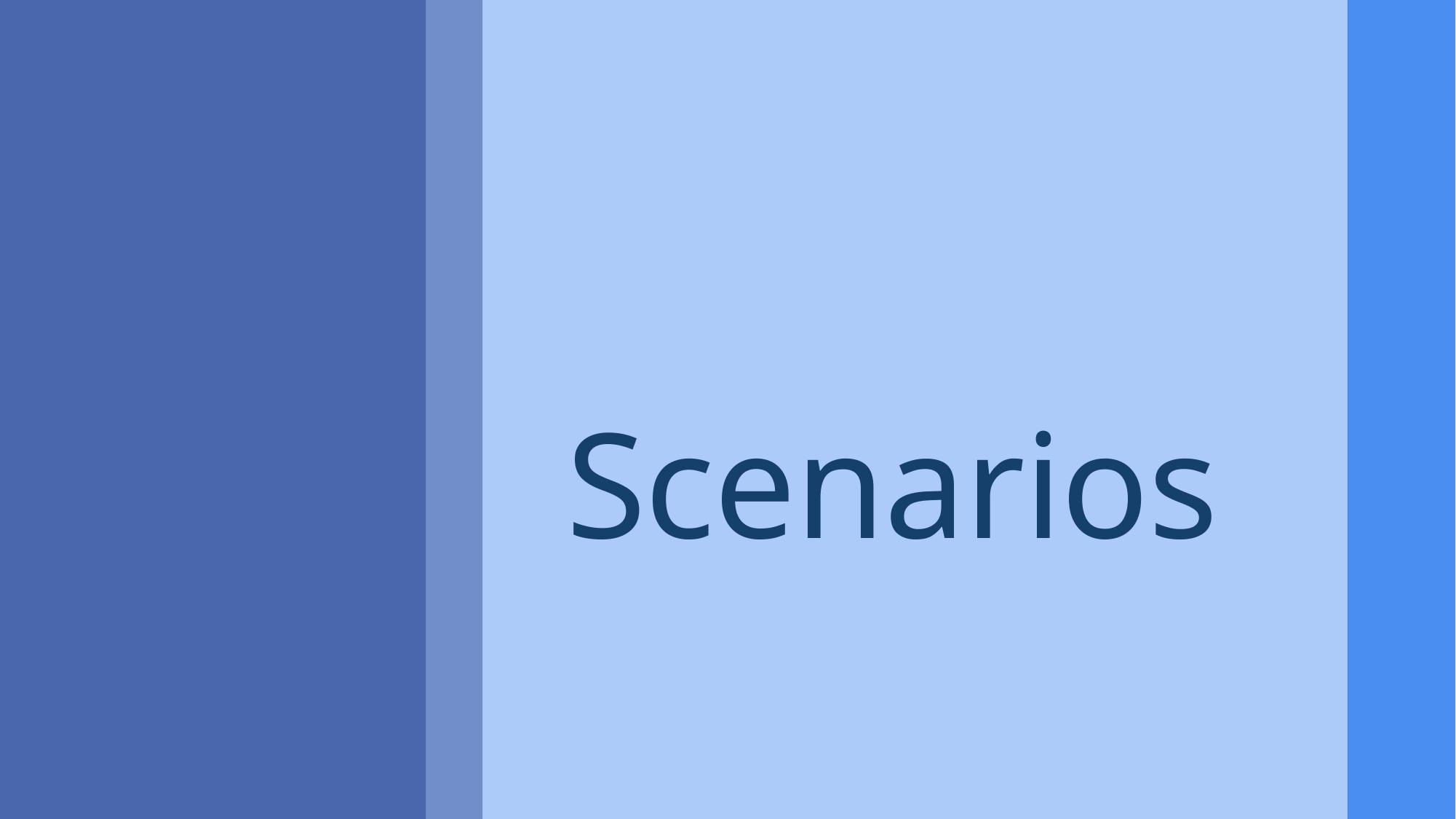

# Scenarios

## Slide 15
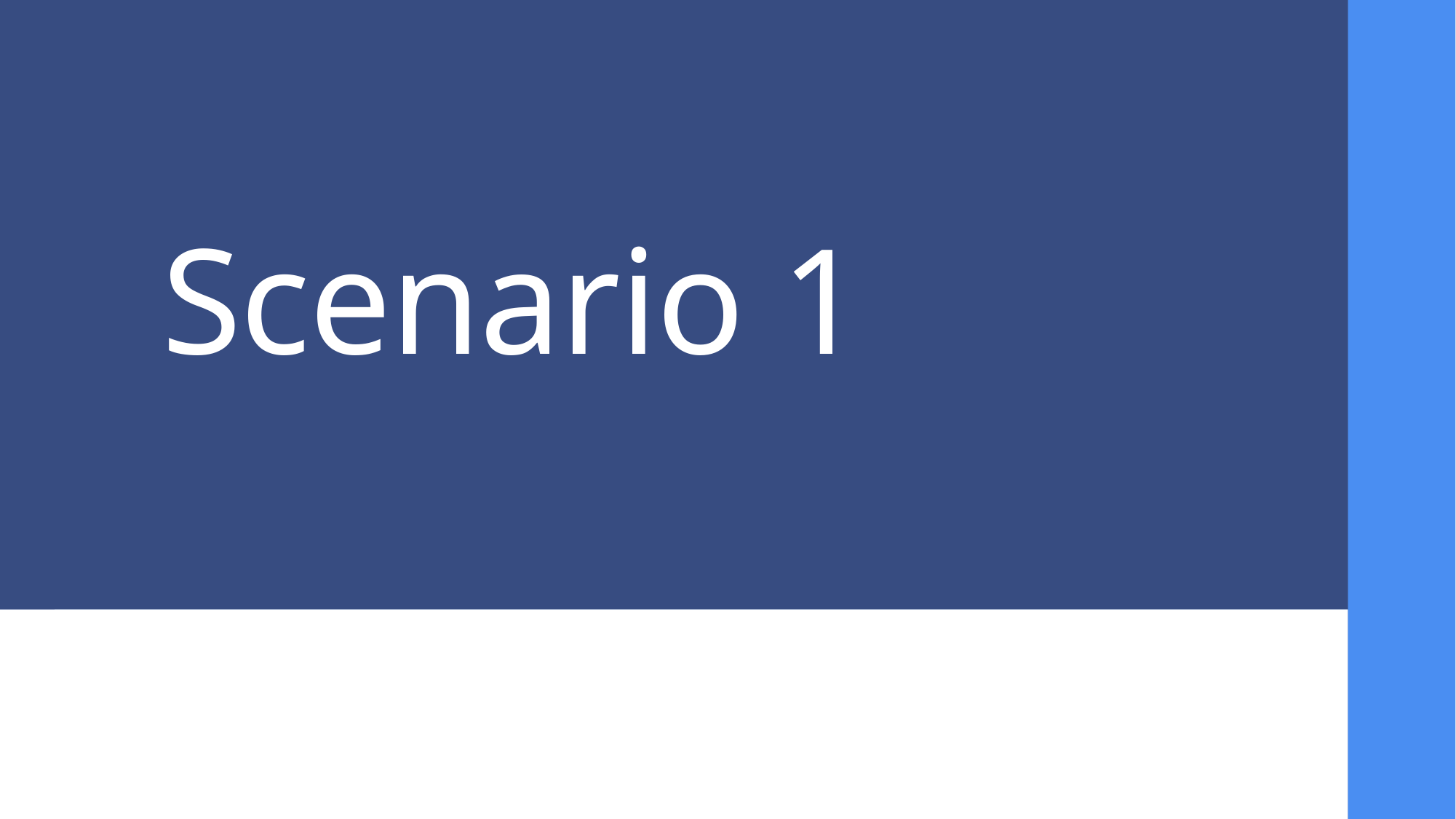

# Scenario 1

## Slide 16
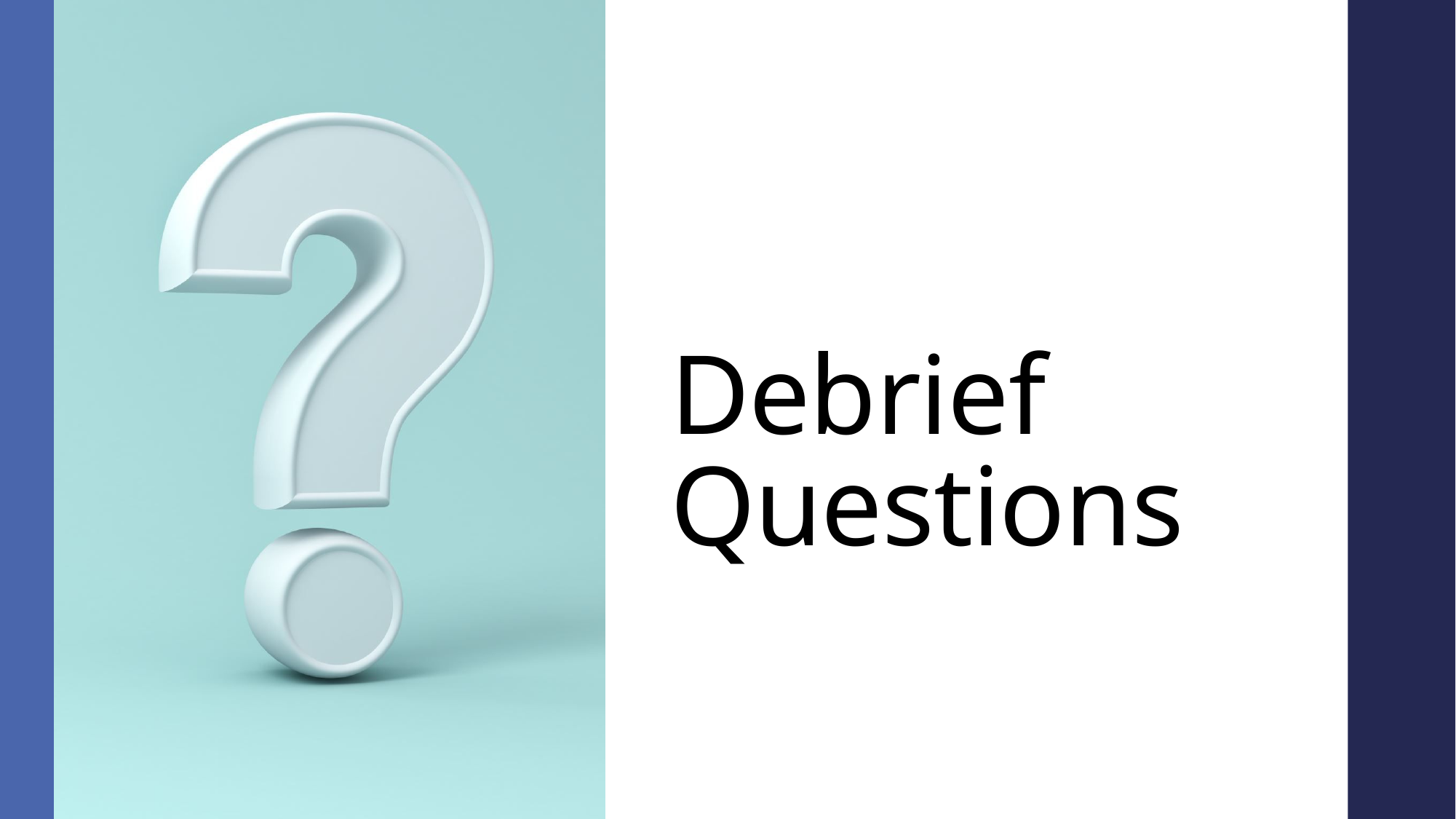

# Debrief Questions

## Slide 17
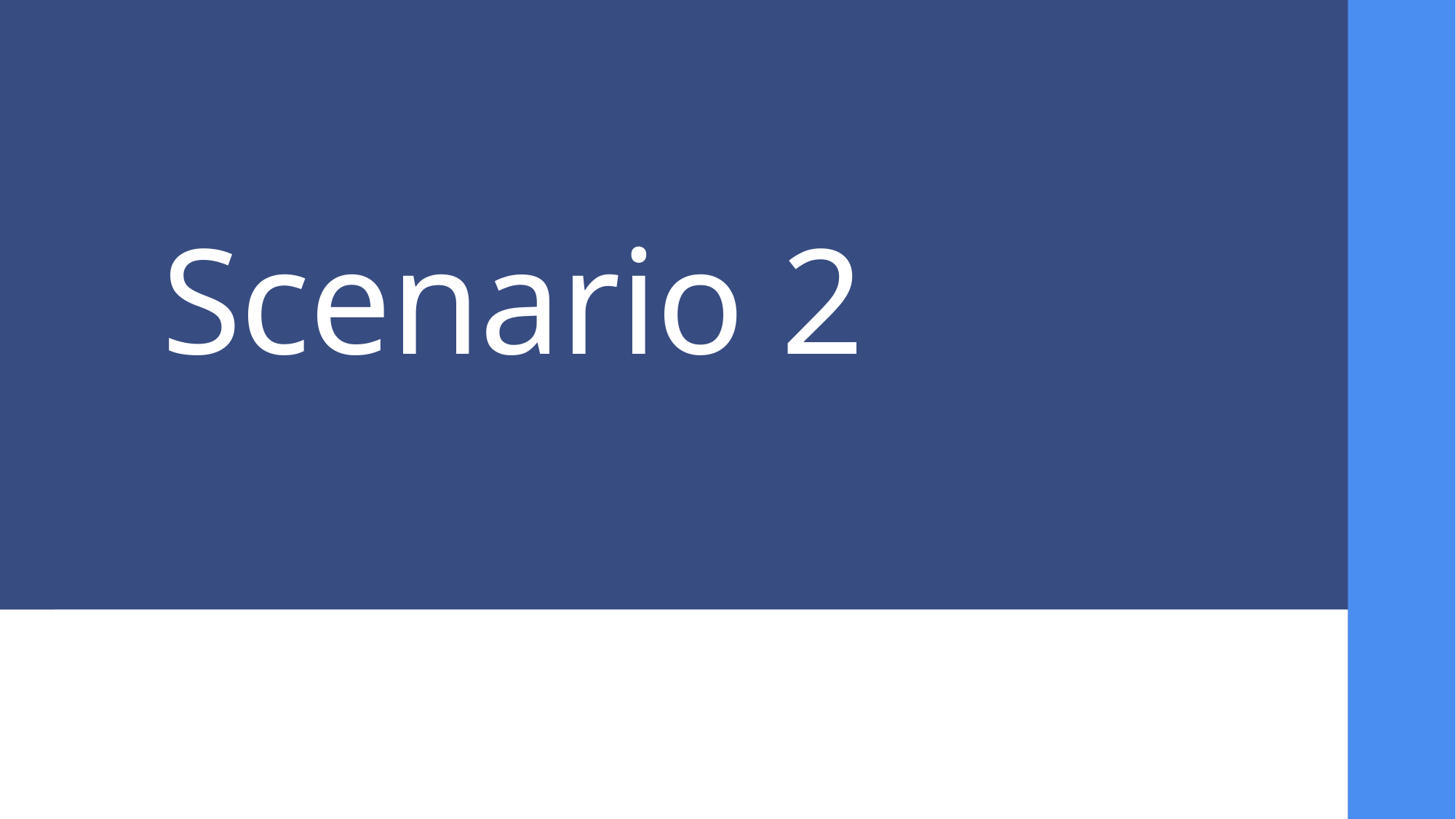

# Scenario 2

## Slide 18
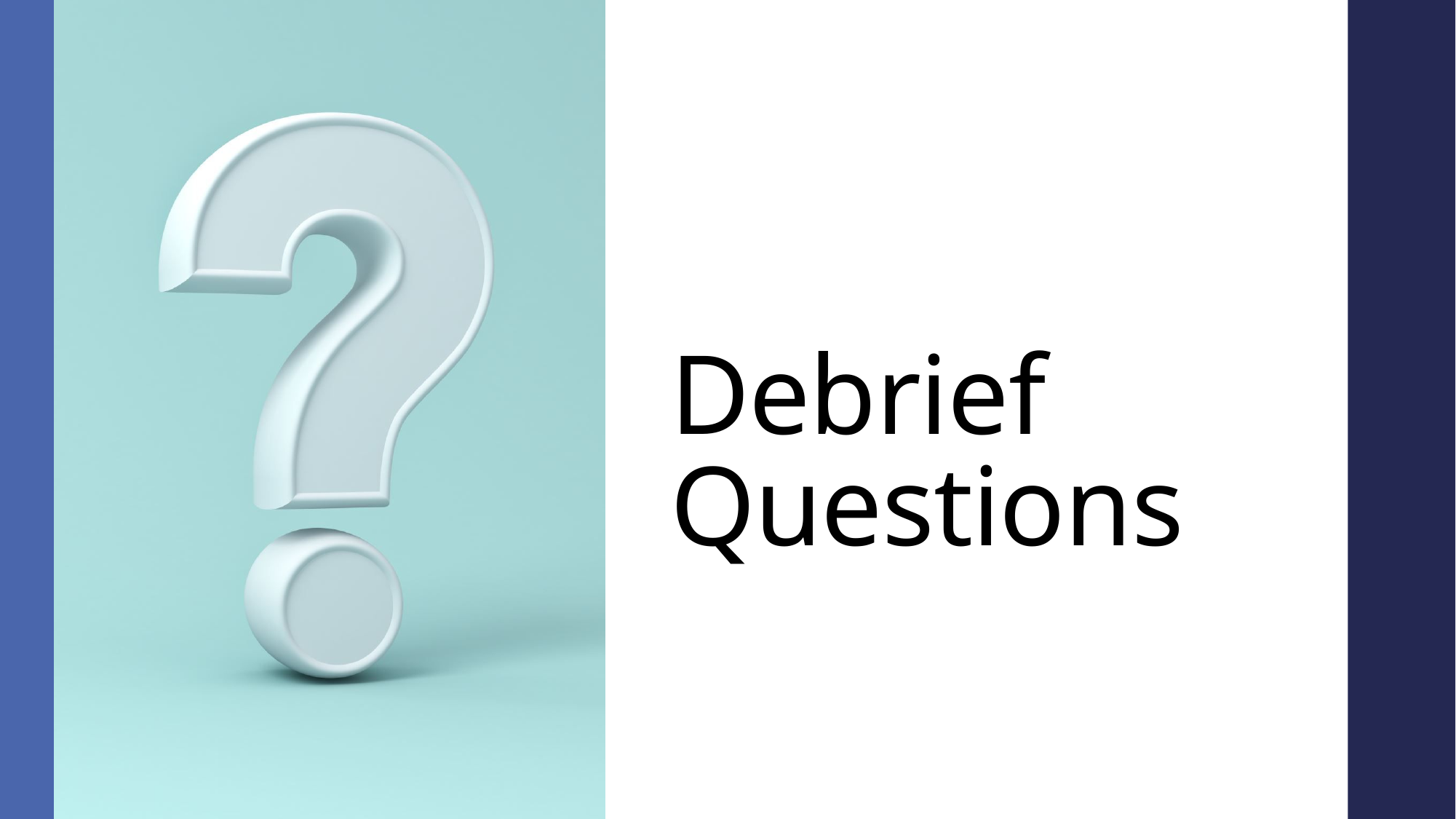

# Debrief Questions

## Slide 19
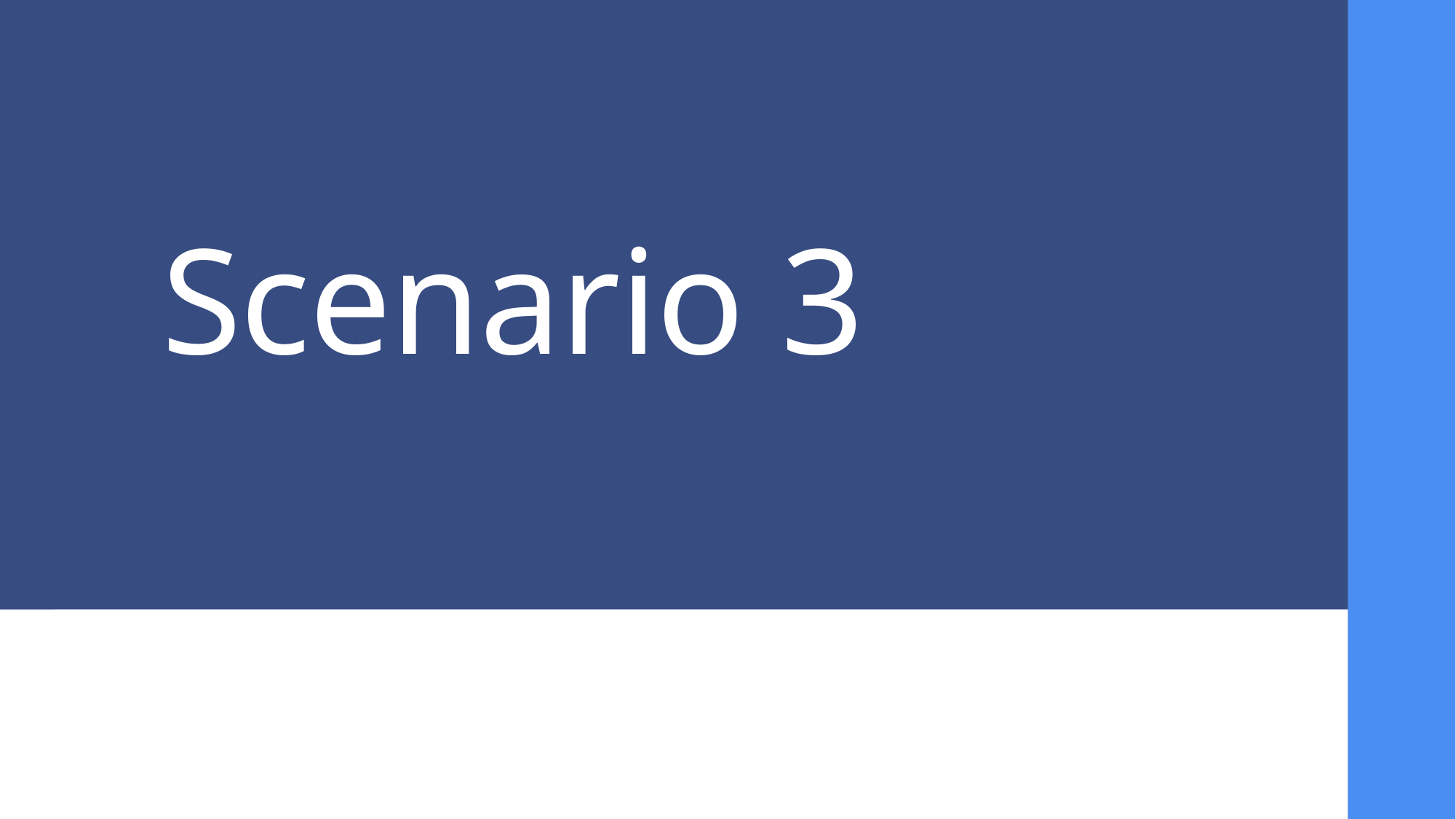

# Scenario 3

## Slide 20
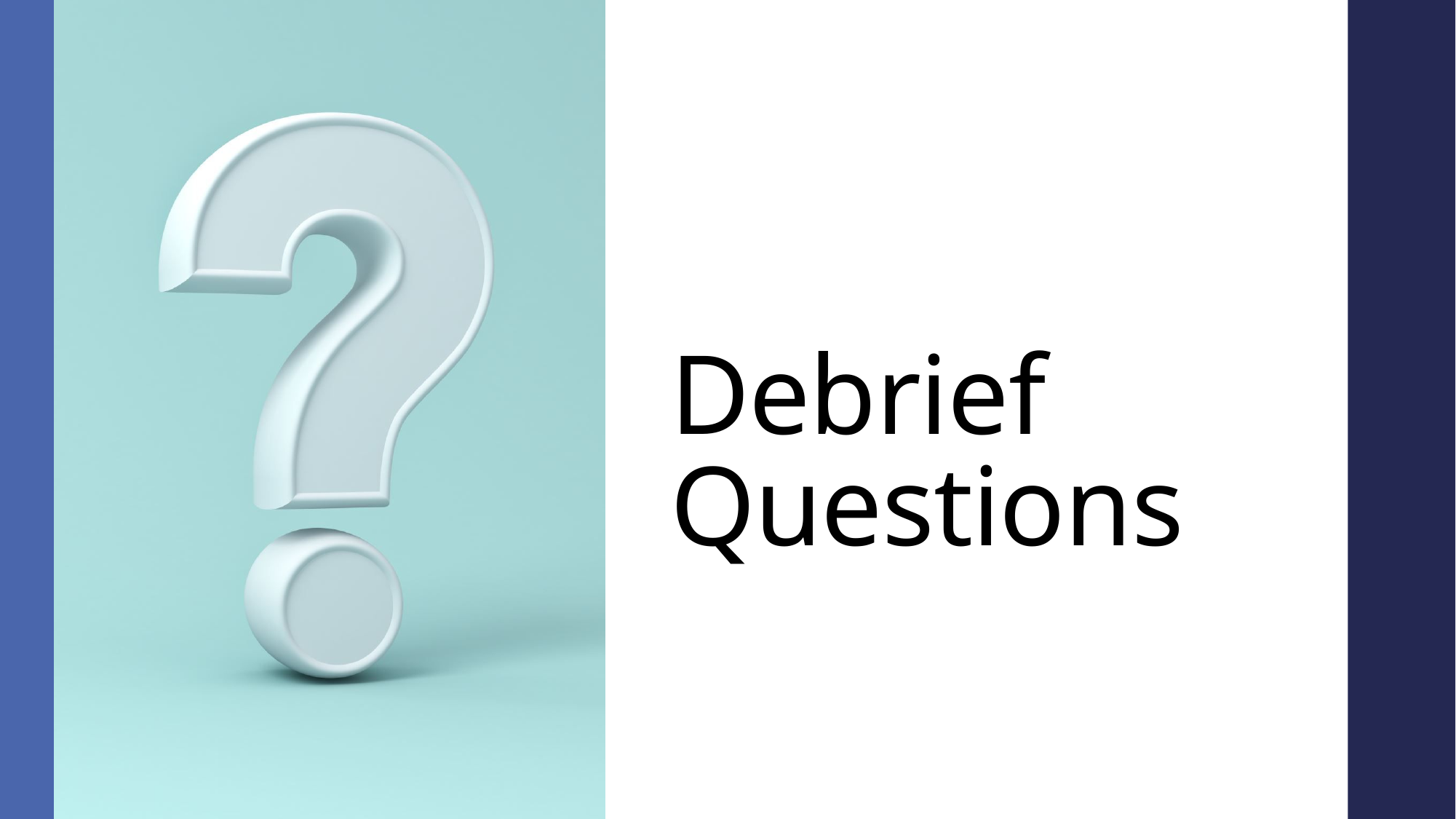

# Debrief Questions

## Slide 21
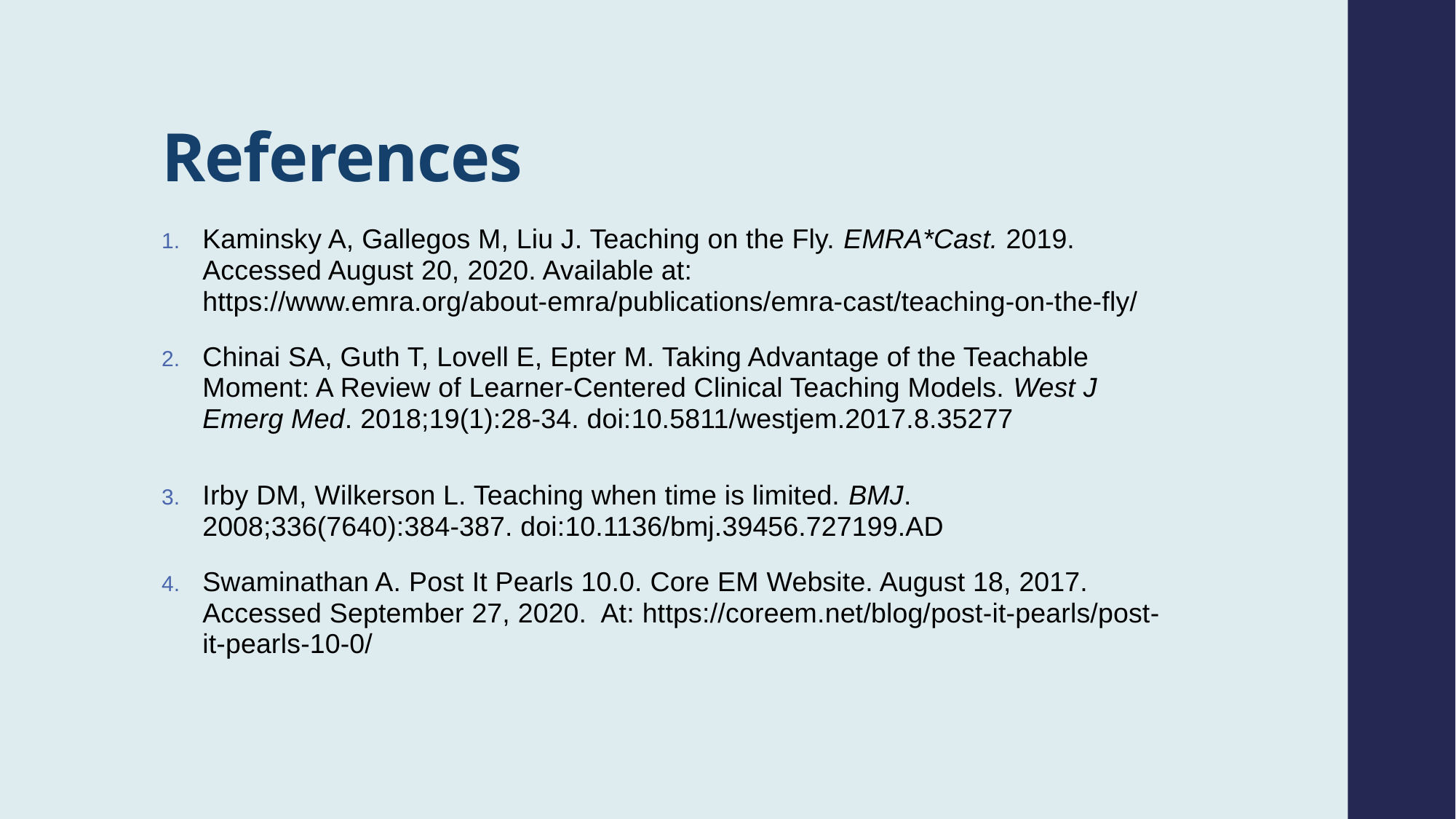

# References
Kaminsky A, Gallegos M, Liu J. Teaching on the Fly. EMRA*Cast. 2019. Accessed August 20, 2020. Available at: https://www.emra.org/about-emra/publications/emra-cast/teaching-on-the-fly/
Chinai SA, Guth T, Lovell E, Epter M. Taking Advantage of the Teachable Moment: A Review of Learner-Centered Clinical Teaching Models. West J Emerg Med. 2018;19(1):28-34. doi:10.5811/westjem.2017.8.35277
Irby DM, Wilkerson L. Teaching when time is limited. BMJ. 2008;336(7640):384-387. doi:10.1136/bmj.39456.727199.AD
Swaminathan A. Post It Pearls 10.0. Core EM Website. August 18, 2017. Accessed September 27, 2020. At: https://coreem.net/blog/post-it-pearls/post-it-pearls-10-0/
